# Sex-specific differences in brain activity dynamics of youth with a family history of substance use disorder

**DOI:** 10.1038/s44220-025-00523-2

**Published:** 2025-11-21

**Authors:** Louisa Schilling, S. Parker Singleton, Ceren Tozlu, Marie Hédo, Qingyu Zhao, Kilian M. Pohl, Keith Jamison, Amy Kuceyeski

**Affiliations:** 1 Department of Radiology, Weill Cornell Medicine, New York, NY USA; 2 Computational Biology, Cornell University, Ithaca, NY USA; 3 Department of Psychiatry & Behavioral Sciences, Stanford University School of Medicine, Stanford, CA USA

**Keywords:** Computational neuroscience, Development of the nervous system

## Abstract

An individual’s risk of substance use disorder (SUD) is shaped by potent biosocial factors. Family history is one of the strongest predictors, yet its impact on the brain before substance exposure remains unclear. Here we apply network control theory to estimate transition energies (TEs)—the input required for the brain to shift between activity patterns—in youth from the Adolescent Brain Cognitive Development Study. Family history of SUD was associated with altered TE, expressed as sex-divergent effects across brain scales alongside elevated TE in specific regions in both sexes. Females with a family history showed higher TE in the default mode network, whereas males showed lower TE in dorsal and ventral attention networks. These findings demonstrate sex-specific influences of family history on brain dynamics and underscore the importance of considering sex as a biological variable when studying adolescent neurodevelopment and mechanisms of SUD risk.

## Abstract (web summary)

This study investigates how family history influences brain dynamics related to substance use disorder, utilizing network control theory to reveal sex-specific alterations in transition energies across brain regions, highlighting the need for sex as a biological variable in neurodevelopment studies.

## Main

Substance use disorder (SUD) has devastating consequences, including familial and financial instability, poor health outcomes and, far too often, death^1^. It remains unclear why only a subset of individuals develop SUD, despite the commonplace use of substances^2^. Dual-systems theories propose that SUD stems from heightened bottom–up reward sensitivity and underdeveloped top–down inhibitory control, an imbalance that peaks in adolescence when prefrontal maturation lags behind that of subcortical reward systems^3–6^. Adolescents with a family history (FH) of SUD (FH+) may experience a premorbid exaggeration of this imbalance due to both genetic and environmental factors^7–10^. Compared to youth without FH (FH−), FH+ youth are at greater risk for SUD and show behavioral and neurocognitive alterations, even before substance use^11–13^.

Sex assigned at birth (hereafter ‘sex’) also shapes SUD vulnerability^14–16^. We use the terms ‘female’ and ‘male’ to refer to sex, but note that sex does not equate to gender—which has distinct neural correlates and influences on SUD risk^14,17,18^—and differences reflect group-level tendencies with substantial overlap^19^. Evidence suggests sex-modulated reinforcement sensitivity: females are more influenced by negative reinforcement (for example, alleviating distress), whereas males are more influenced by positive reinforcement (for example, drug reward)^16^. These mechanisms may underlie observed patterns, with females escalating more rapidly due to heightened withdrawal and craving, and males initiating earlier and developing SUD at higher rates^14,16,20,21^. Similar patterns are evident in adolescence, where internalizing pathways are more common in females and externalizing pathways in males^22–25^. Neuroimaging studies mirror these behavioral patterns, with men showing greater reward-related impulsivity and women showing heightened negative emotionality in task-based functional MRI (fMRI) paradigms across both adults^16,26^ and adolescents^27,28^. Emerging evidence further suggests that FH of SUD may amplify these baseline sex differences in responses to reward and stress, as indicated by both behavioral^29–31^ and neuroimaging studies^32–38^, although evidence remains limited.

Neuroimaging findings in FH+ youth reveal alterations in mesocorticolimbic regions (for example, prefrontal cortex, striatum and amygdala) that parallel those observed in adults with SUD—alterations previously presumed to solely reflect the consequences of chronic substance use^39^. These parallels span functional activity^10,40–44^, dopaminergic signaling^45,46^, white matter integrity^35,47–50^, gray-matter volume^48,51,52^ and cortical thickness^32,53,54^. Together, this evidence suggests that neural correlates associated with SUD may, in part, reflect premorbid vulnerability^51^.

Yet these alterations in FH+ individuals and those with SUD are not confined to isolated regions, but probably reflect changes in large-scale brain networks. Such networks undergo extensive reorganization during development to support cognitive functions including reward processing and inhibition^55–57^. Premorbid disruptions in the activity and functional connectivity of these networks have been identified in youth who later develop SUD^55,58^ and in FH+ youth^36,43,44,59–62^, particularly in networks also implicated in SUD: default mode (DMN), frontoparietal (FPN) and salience/ventral attention (VAT) networks^63–67^. Sex modulates the manifestation of SUD in these networks^37,38,68^, but there is limited research on the interaction of FH and sex. One study found no FH-by-sex effects on functional connectivity^59^, whereas another study identified a three-way interaction of sex, childhood maltreatment and FH on activity in the FPN, executive control and DMN networks^69^. Both, however, included older adolescents with previous substance exposure, limiting interpretation.

Functional networks enable flexibly shifting between internally and externally oriented brain states^55,56,70^. The dynamics of these transitions differ by sex, with females exhibiting less overall dynamism^71^. SUD, too, alters these dynamics, with less time spent in internal states, more in external states^72–74^, and fewer transitions overall^65^. FH+ young adults, particularly males, show a reduction in a reconfiguration process in visual, DMN and attention networks associated with task-to-rest transitions^75^. Together, these findings suggest that FH affects the ability to shift between brain states and is likely modulated by sex.

Network control theory (NCT) is a powerful framework for capturing individual differences in brain dynamics. Unlike traditional metrics of functional activation or connectivity, NCT models how activity propagates across structural connections to support brain-state transitions^76^. The ease of these transitions is quantified as the transition energy (TE), the cumulative input required to steer the brain from one state to another^76^. TE indexes internal cognitive demands rather than direct metabolic costs, although it has recently been linked to metabolic activity^77,78^. By directly estimating the effort needed to reconfigure activity across networks, TE provides an apt metric for investigating vulnerability to disorders characterized by imbalance in top–down and bottom–up control. Earlier work has linked altered TE to alcohol use^65^, methamphetamine abstinence^79^, dopaminergic dysfunction^80^, psychopathology^57^ and sex-linked impulsivity^81^. However, the relationship between FH of SUD and TE during adolescence has not yet been assessed.

The literature on FH+ individuals is difficult to interpret due to methodological heterogeneity, small samples, wide age ranges, prior substance exposure and limited attention to sex. Nonetheless, four themes emerge: (1) SUD risk arises in adolescence from an imbalance of top–down and bottom–up control; (2) sex modulates this risk through distinct neurobehavioral pathways; (3) FH+ adolescents show a premorbid exaggeration of this neurodevelopmental profile, probably via altered network dynamics; and (4) FH and sex interact and may amplify baseline sex differences. However, no study has directly tested how sex and FH jointly influence brain-state dynamics. To address this gap, we applied NCT to functional and structural neuroimaging data from substance-naïve youth in the Adolescent Brain Cognitive Development Study (ABCD Study)^82^, quantifying sex-specific differences in TE between recurring brain states in FH+ versus FH− youth. By characterizing these premorbid dynamics, our work aims to clarify the neurobiological basis of SUD risk and to inform prevention and intervention strategies in vulnerable populations.

## Results

### Sample characteristics

To investigate sex-specific differences in the transition energies of youth with (FH+) and without (FH−) FH of SUD, we used diffusion MRI (dMRI) and resting-state functional MRI (rsfMRI) data from the ABCD Study’s baseline assessment of a large sample of substance-naïve youth (*N* = 1,886 individuals, 10.02 ± 0.62 years, 53% female) (Table 1). We classified individuals as FH+ if they had at least one parent or two grandparents with a history of SUD, and FH− if they had no parents or grandparents with a history^32,54,83^. Individuals with just one grandparent with a history of SUD were classified as FH+/− and are included only in analyses of continuous associations (that is, FH density, FHD). See ‘Exclusions’ section for the exclusion criteria. Demographic comparisons indicated no significant differences between FH groups in terms of sex, age, framewise displacement or MRI scanner model distribution. However, FH+ individuals tended to have lower household income, greater racial/ethnic diversity, lower parental education status, higher rates of prenatal substance exposure and parental mental health issues, and more advanced pubertal stages.

**Table 1:** Demographic and characteristic data for the full cohort and subgroups (FH+, FH−, FH+/−)

	Full cohort	FH+	FH−	FH+/−	*P* value
**Sample size**					
	1,886 (100.0%)	436 (23.1%)	1,175 (62.3%)	275 (14.6%)	
**Sex assigned at birth**					0.451
Male	885 (46.9%)	198 (45.4%)	549 (46.7%)	138 (50.2%)	
Female	1,001 (53.1%)	238 (54.6%)	626 (53.3%)	137 (49.8%)	
**Age in months**					0.426
	120.2 (±7.5)	119.9 (±7.5)	120.3 (±7.5)	120.5 (±7.0)	
**Puberty status: female**					0.123
Pre-puberty	322 (32.2%)	70 (29.4%)	210 (33.6%)	42 (30.7%)	
Early puberty	259 (25.9%)	52 (21.9%)	165 (26.4%)	42 (30.7%)	
Mid/late	420 (42.0%)	116 (48.7%)	251 (40.1%)	53 (38.7%)	
**Puberty status: male**					**0.035**
Pre-puberty	672 (75.9%)	143 (72.2%)	417 (76.0%)	112 (81.1%)	
Early puberty	170 (19.2%)	42 (21.2%)	112 (20.4%)	16 (11.6%)	
Mid/late	43 (4.9%)	13 (6.6%)	20 (3.6%)	10 (7.2%)	
**Framewise displacement**					0.124
	0.12 (±0.08)	0.12 (±0.08)	0.12 (±0.07)	0.12 (±0.08)	
**MRI scanner model**					0.135
GE Discovery MR750	524 (27.8%)	140 (32.1%)	313 (26.6%)	71 (25.8%)	
Siemens Prisma	691 (36.6%)	141 (32.3%)	448 (38.1%)	102 (37.1%)	
Siemens Prisma Fit	671 (35.6%)	155 (35.6%)	414 (35.2%)	102 (37.1%)	
**Household income**					**2.37** × **10**^−**13**^
<$50,000	426 (22.6%)	151 (34.6%)	229 (19.5%)	46 (16.7%)	
$50,000–100,000	563 (29.9%)	141 (32.3%)	332 (28.3%)	90 (32.7%)	
$100,000+	897 (47.5%)	144 (33.0%)	614 (52.3%)	139 (50.6%)	
**Parental education**					**2.24** × **10**^−**15**^
<High school	49 (2.6%)	12 (2.8%)	35 (3.0%)	2 (0.7%)	
High school/GED	125 (6.6%)	51 (11.7%)	66 (5.6%)	8 (2.9%)	
Some college	184 (9.8%)	60 (13.8%)	94 (8.0%)	30 (10.9%)	
Associates/bachelor	765 (40.6%)	209 (47.9%)	451 (38.4%)	105 (38.2%)	
Postgraduate	763 (40.5%)	104 (23.9%)	529 (45.0%)	130 (47.3%)	
**Race/ethnicity**					**9.84** × **10**^−**6**^
White	1,183 (62.7%)	251 (57.6%)	746 (63.5%)	186 (67.6%)	
Black	144 (7.6%)	50 (11.5%)	76 (6.5%)	18 (6.6%)	
Hispanic/Latinx	345 (18.3%)	85 (19.5%)	212 (18.0%)	48 (17.5%)	
Asian	38 (2.0%)	0 (0.0%)	37 (3.2%)	1 (0.4%)	
Other	176 (9.3%)	50 (11.5%)	104 (8.9%)	22 (8.0%)	
**Parental mental health issues**					**8.82** × **10**^−**48**^
Yes	984 (52.2%)	354 (81.2%)	473 (40.3%)	157 (57.1%)	
No	902 (47.8%)	82 (18.8%)	702 (59.7%)	118 (42.9%)	
**Prenatal substance exposure**					**5.68** × **10**^−**16**^
Yes	122 (6.5%)	62 (14.2%)	38 (3.2%)	22 (8.0%)	
No	1,764 (93.5%)	374 (85.8%)	1,137 (96.8%)	253 (92.0%)	
**FHD**					–
	0.40 (±0.64)	1.41 (±0.57)	0.00 (±0.00)	0.50 (±0.00)	

Categorical variables are displayed as *N* (%) with *P* values from χ^2^ tests (within sex for puberty status). Continuous variables are displayed as mean (±s.d.) with *P* values from one-way analysis of variance (ANOVA; *F*-tests). No group comparison was performed for FHD (fixed at 0.50 for FH+/− and 0.00 for FH−). Significant *P* values are shown in bold at *P* < 0.05.

### NCT analysis

**Fig. 1:**
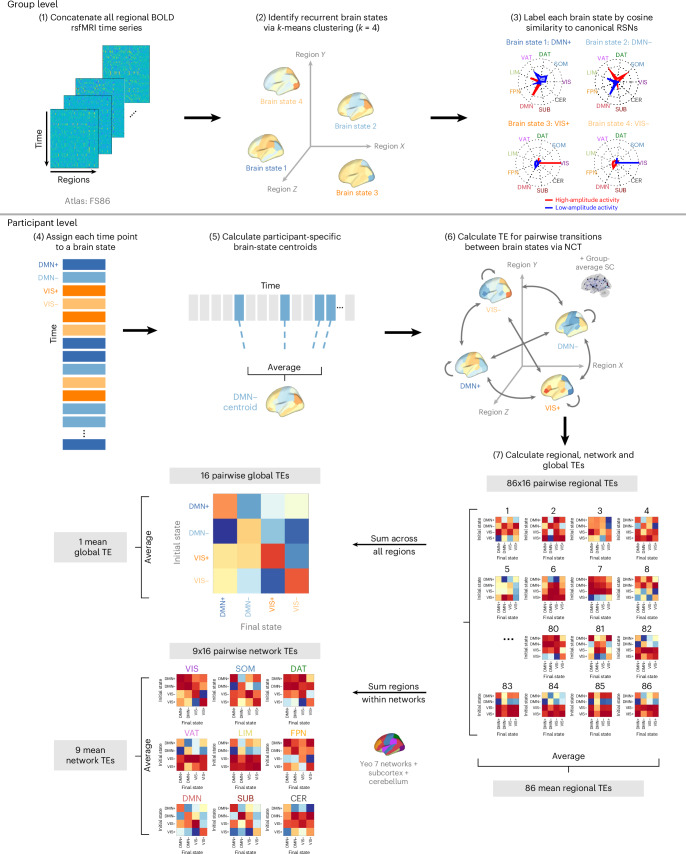
NCT analysis workflow. Steps 1–2: we applied *k*-means clustering to regional rsfMRI time series from all participants to identify four recurring brain states. Step 3: each state was labeled according to the Yeo 7 networks^87^ plus subcortical and cerebellar networks, based on the network showing the highest cosine similarity with the state’s activity pattern. Steps 4–5: next, individual time frames were assigned to brain states, and individual brain-state centroids were computed. Step 6: NCT was then implemented using a group-average SC to estimate the TE required for switching between pairs of individual brain states. Step 7: pairwise and mean TE values were then computed at global, network and regional levels for each participant. CER, cerebellum; DAT, dorsal attention network; DMN, default mode network; FPN, frontoparietal network; LIM, limbic network; RSN, resting-state network; SOM, somatomotor network; SUB, subcortex; VAT, ventral attention network; VIS, visual network.

Following previous work^84,85^, we applied *k*-means clustering to regional rsfMRI time-series data (86-region atlas) to identify *k* recurring patterns of brain activity, termed ‘brain states’ (Fig. 1). For each participant, we assigned each individual frame to a brain state and calculated individual brain-state centroids. We then applied NCT to calculate the global-, network- and region-level TE required to complete brain-state transitions. For this, we utilized a group-average structural connectome (SC; derived from dMRI from a subset of individuals in this dataset) as previously done^80^, and as supported by observations that functional abnormalities precede white-matter changes in FH+ youth^43,86^ (Supplementary Section 10.3 provides a replication of our results using individual SCs). We calculated both the pairwise and mean TE for all levels of analysis—global, network and regional. Initial TE calculations result in ‘pairwise regional TE’ values, that is, 86 (number of regions) *k* × *k* matrices. For each matrix, we summed the pairwise regional TE of all regions belonging to each of the seven Yeo networks^87^ plus subcortical and cerebellar networks to yield ‘pairwise network TE’ values, that is, nine (number of networks) *k* × *k* matrices. We derived a single ‘pairwise global TE’ *k* × *k* matrix by summing across all 86 regions’ pairwise TEs for each transition *k* × *k* matrix. We also averaged all entries in pairwise TE matrices to derive mean TE values for each region, network and globally, resulting in 86 mean regional TE values, nine mean network TEs, and a single value for mean global TE. Thus, pairwise TE represents the energy required of a given region, network or whole brain to complete transitions between each pair of brain states, whereas mean TE represents the energy required to transition across the entire state space (that is, all pairwise transitions). The section ‘TE calculations’ provides more details.

It is important to distinguish the four brain states from the nine networks used to calculate TE values, although both derive from a common nine-network parcellation (Yeo 7 networks plus subcortical and cerebellar networks)^87^. Brain states are the four *k*-means clusters assigned to the network whose high or low activity best explained each centroid. These descriptive labels did not influence analyses and are denoted with ‘+’ or ‘−’ to indicate above- or below-mean activity (for example, DMN+, VIS−). Network TE values, by contrast, are calculated by summing regional TEs across all regions in a given network and are referenced using the nine network names alone (for example, DAT, VIS, DMN).

We conducted a series of two-way analyses of covariance (ANCOVA) to examine the effects of FH of SUD and its interaction with sex on mean and pairwise TEs at global, network and regional levels. All models included the following independent variables: sex, age, FH of SUD (FH+ versus FH−), race/ethnicity, household income, parental education, parental mental health issues, prenatal substance exposure, MRI scanner model, in-scanner motion (mean framewise displacement) and puberty status. Additionally, three interaction terms were included: puberty and sex, FH of SUD and sex, and FH of SUD and income. The direction of effects was determined using post hoc unpaired *t*-tests on TE values that showed significant differences in ANCOVA models. To validate our findings, we used a continuous measure of FHD (number of affected first- and second-degree relatives) and performed Spearman’s rank correlations with significant TE values^54^ across all three groups (FH+, FH−, FH+/−). Effect sizes were reported as partial *η*^2^ for ANCOVA model results and Cohen’s *d* for the results of *t*-tests. To account for multiple comparisons, *P* values were adjusted using the Benjamini–Hochberg false discovery rate (FDR) procedure (*q* = 0.05), with *P*_FDR_ < 0.05 considered significant^88^.

### Brain state identification

We found an optimal solution of *k* = 4 brain states, determined using a cutoff of additional explained variance of <1% (Supplementary Fig. 1). The identified brain states consist of two pairs of anticorrelated activity patterns (that is, meta-states), the first dominated by high- and low-amplitude activity in the default mode network (DMN+/−) and the second by high- and low-amplitude activity in the visual network (VIS+/−) (Supplementary Fig. 3), aligning with previous work^65,84^. For replication of our results using *k* = 5, see Supplementary Fig. 13.

### Global TE

**Fig. 2:**
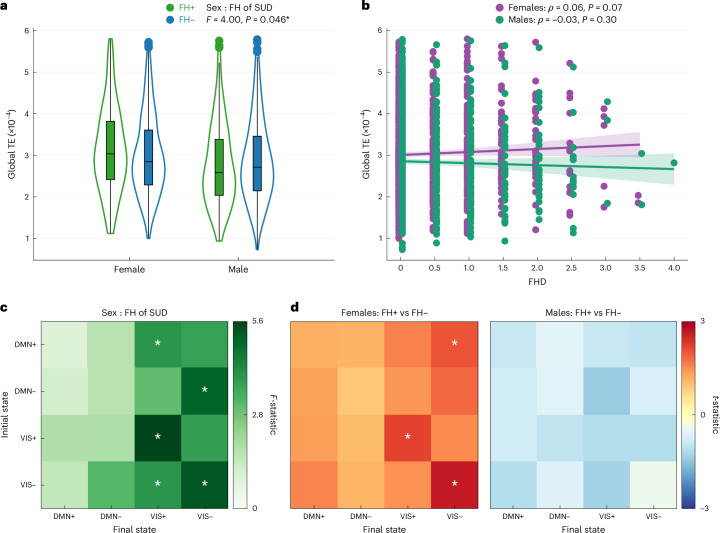
Global TE differs by sex and FH of SUD. **a**, ANCOVA on mean global TE (one value per participant; *N* = 1,611: 238 FH+ females, 626 FH− females, 198 FH+ males, 549 FH− males) revealed a nominally significant FH-by-sex interaction such that FH+ > FH− in females and FH+ < FH− in males. The violin plots show the full data distribution. Box plots display the median (line), interquartile range (25th to 75th percentiles), whiskers (1.5× interquartile range, IQR) and individual outliers. **b**, Spearman rank correlations revealed a weak, non-significant positive association between FHD and global TE in females (purple; *N* = 1,001) and no association in males (green; *N* = 885). Lines reflect generalized linear model fits, with shaded 95% confidence intervals. Each point represents a single participant, and points are jittered for visibility. **c**, ANCOVA on pairwise global TE (16 values per participant) revealed that the FH-by-sex interaction was primarily driven by transitions to the VIS+/− states. *N* = 1,611: 238 FH+ females, 626 FH− females, 198 FH+ males, 549 FH− males. **d**, Unpaired *t*-tests indicated that FH+ females had higher pairwise TE than FH− females across transitions, whereas FH+ males showed lower TE than FH− males (non-significant). *N* = 1,611: 238 FH+ females, 626 FH− females, 198 FH+ males, 549 FH− males. All tests were two-sided. Exact *P* values, test statistics (for example, *F*, *t*), degrees of freedom and effect sizes are reported in the figure or source data. *P < 0.05 (uncorrected); not significant after Benjamini–Hochberg correction. Source data

We first examined whether FH of SUD or its interaction with sex had a measurable global impact on the brain’s overall energetic landscape. In a two-way ANCOVA model assessing mean global TE, the main effect of FH of SUD was non-significant (*F*(1, 1,588) = 0.05, *P* = 0.82, ${\eta }_{p}^{2}{<}0.001$). By contrast, the interaction between sex and FH of SUD reached nominal significance before correction (*F*(1, 1,588) = 4.00, *P* = 0.046, *P*_FDR_ = 0.128, ${\eta }_{p}^{2}=0.003$). The lack of a main effect was partly driven by the opposite direction of effect in the sexes. Within-sex post hoc *t*-tests revealed FH+ females had higher mean global TE than FH− females (*t* = 1.73, *P* = 0.084, Cohen’s *d* = 0.13), whereas FH+ males had lower mean global TE than FH− males (*t* = −1.14, *P* = 0.256, Cohen’s *d* = −0.09) (Fig. 2a). Extending the analysis to include FH+/− individuals (that is, those with one grandparent with SUD) revealed weak, non-significant correlations between FHD and mean global TE, trending in the same direction as the categorical effects: a positive association in females (Spearman’s *ρ* = 0.06, *P* = 0.073) and a negative association in males (Spearman’s *ρ* = −0.04, *P* = 0.302) (Fig. 2b).

The main effect of sex was a significant effect on mean global TE (*F*(1, 1,588) = 25.93, *P* = 3.96 × 10^−7^, *P*_FDR_ = 2.77 × 10^−6^, ${\eta }_{p}^{2}=0.019$). Post hoc analyses using two-sided, unpaired *t*-tests confirmed that females exhibited higher mean global TE compared to males (*t* = 4.29, *P* = 1.89 × 10^−5^, Cohen’s *d* = 0.20). Among all subgroups, FH+ females showed the highest mean global TE, followed by FH− females, FH− males and FH+ males.

Additionally, parental history of mental health issues significantly (before correction) affected mean global TE (*F*(1, 1,585) = 6.05, *P* = 0.014, *P*_FDR_ = 0.065, ${\eta }_{p}^{2}=0.004$), driven by higher TE in youth with parental history of mental illness compared to without such history (*t* = 3.04, *P* = 0.002, Cohen’s *d* = 0.14). Other significant factors included MRI scanner model (*F*(2, 1,585) = 24.98, *P* = 2.08 × 10^−11^, *P*_FDR_ = 2.91 × 10^−10^, ${\eta }_{p}^{2}=0.031$) and the interaction of sex and puberty stage (*F*(2, 1,585) = 3.126, *P* = 0.043, *P*_FDR_ = 0.668, ${\eta }_{p}^{2}=0.004$). Supplementary Table 4 presents the full ANCOVA results and Supplementary Fig. 18 additional analyses stratified by scanner type.

Two-way ANCOVA models for entries in the pairwise global TE matrix indicated that the effect of the interaction of sex and FH of SUD was strongest in transitions to and persistence within the VIS meta-state (Fig. 2c). Post hoc unpaired *t*-tests showed that this interaction effect was primarily driven by greater pairwise global TE in FH+ females versus FH− females in transitions to the VIS meta-state (Fig. 2d). All pairwise transitions had higher TE in FH+ females and lower TE in FH+ males compared to their sex-matched FH− counterparts.

### Network TE

**Fig. 3:**
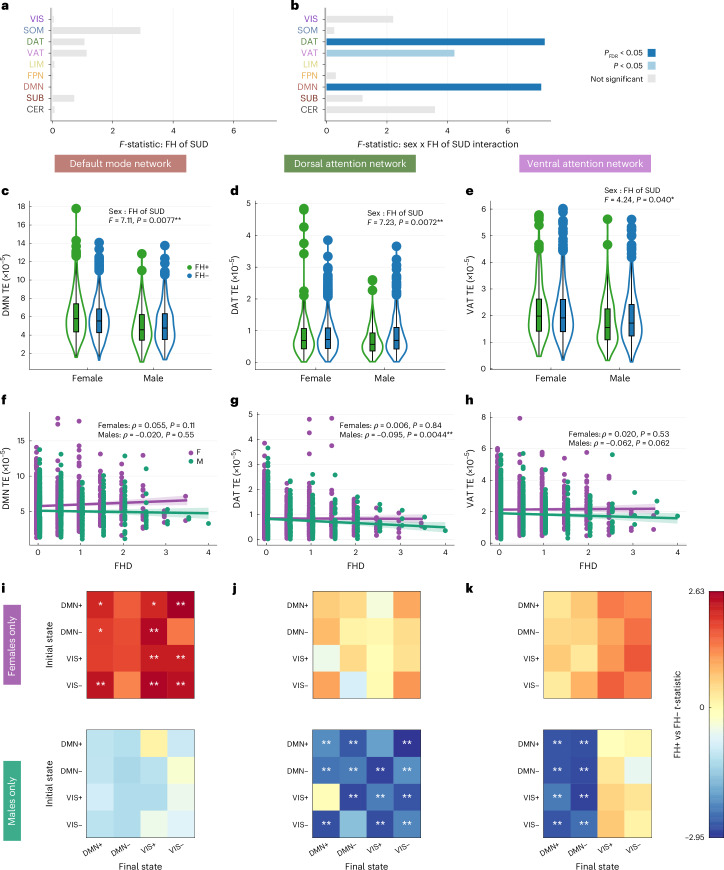
Network-level TE differs by sex and FH of SUD. **a**,**b**, ANCOVA models performed on mean network TE across nine canonical brain networks (nine values per participant; *N* = 1,611; 238 FH+ females, 626 FH− females, 198 FH+ males, 549 FH− males) showed no main effect of FH (**a**), but a significant FH-by-sex interaction in the DMN and DAT networks (*P*_FDR_ < 0.05, Benjamini–Hochberg corrected), and an uncorrected effect in the VAT network (*P* < 0.05) (**b**). Bar plots show *F* statistics, with color indicating significance before (light blue) and after (dark blue) multiple-comparison correction. **c**–**e**, Violin plots showing kernel density estimates. Box plots indicate IQR, median (line), whiskers (1.5× IQR) and individual outliers for mean network TE in the DMN (**c**), DAT (**d**) and VAT (**e**). FH+ females (*N* = 238) showed higher TE than FH− females (*N* = 626) in the DMN, and FH+ males (*N* = 198) showed lower TE than FH− males (*N* = 549) in the DAT and VAT. **f**–**h**, Spearman correlations between FHD and mean network TE showing a weak positive association in the DMN in females (**f**, purple; *N* = 1,001) and significant negative associations in males (green; *N* = 885) in the DAT (**g**) and VAT (**h**). Solid lines indicate linear fits from generalized linear models. Shaded bands represent 95% confidence intervals. Each point represents a single participant, and points are jittered for visibility. **i**–**k**, *t*-tests of pairwise TE values (16 transitions per participant) supported these effects: FH+ females (*N* = 238) compared to FH− females (*N* = 626) had higher pairwise DMN TE especially in transitions to the VIS+/− states (**i**); FH+ males (*N* = 198) compared to FH− males (*N* = 549) had lower pairwise network TE in the DAT (**j**) across most transitions, and in the VAT (**k**), particularly in transitions to the DMN+/− states. All tests were two-sided. Multiple comparisons were controlled with Benjamini–Hochberg FDR (*q* = 0.05) across families of tests. Exact *P* values, test statistics (for example, *F*, *t*), degrees of freedom and effect sizes are reported in the figure or source data. *P < 0.05 before correction; **P_FDR_ < 0.05. Source data

We next investigated which networks are driving the observed effects in global TE. For each network, we ran two-way ANCOVAs for mean network TE using the same covariates described above. Mean network TE did not show a significant main effect of FH of SUD in any network (Fig. 3a), but significant FH-by-sex interaction effects were observed in the DMN, DAT and VAT networks, although the VAT effect did not survive FDR correction across the nine networks (Fig. 3b). Full ANCOVA results for all networks are presented in Supplementary Information (Supplementary Fig. 6).

To examine the direction of these interaction effects, we performed unpaired *t*-tests comparing FH+ and FH− individuals within each sex. In the DMN, the interaction was driven by higher mean TE in FH+ compared to FH− females (*t* = 2.55, *P* = 0.011, *P*_FDR_ = 0.027, Cohen’s *d* = 0.19), but no significant difference was seen in FH+ versus FH− males (*t* = −1.19, *P* = 0.233, Cohen’s *d* = −0.10) (Fig. 3c). Supporting this pattern, FHD of SUD in females had a weak trending positive correlation with mean DMN TE (*ρ* = 0.051, *P* = 0.105; Fig. 3f). Conversely, in the DAT and VAT networks, the interaction effects were driven by significantly lower mean network TE in FH+ compared to FH− males (DAT: *t* = −3.38, *P* = 0.001, *P*_FDR_ = 0.005, Cohen’s *d* = −0.28; VAT: *t* = −2.48, *P* = 0.014, *P*_FDR_ = 0.027, Cohen’s *d* = −0.21), whereas females showed no significant differences in mean TE of these networks (DAT: *t* = 0.46, *P* = 0.646, Cohen’s *d* = 0.04; VAT: *t* = 0.90, *P* = 0.366, Cohen’s *d* = 0.07) (Fig. 3d,e). Finally, we found significant, albeit weak, negative correlations between mean network TE and FHD in males for both the DAT (*ρ* = −0.095, *P* = 0.004) and VAT (*ρ* = −0.063, *P* = 0.062) networks, though the VAT result did not survive FDR correction (Fig. 3g,h).

We next investigated which pairwise state transitions drive the observed effects of FH of SUD and sex on mean network TE. Within-sex unpaired *t*-tests revealed greater pairwise DMN TE in FH+ compared to FH− females across all transitions, particularly in transitions to the VIS meta-state and to DMN−, whereas FH+ and FH− males exhibited no significant differences in pairwise DMN TE (Fig. 3h). FH+ males had lower pairwise DAT TE for almost all transitions compared to FH− males (Fig. 3i) and lower pairwise VAT TE only in transitions to the DMN meta-state (Fig. 3j), whereas no differences were observed in FH+ versus FH− females.

### Regional TE

**Fig. 4:**
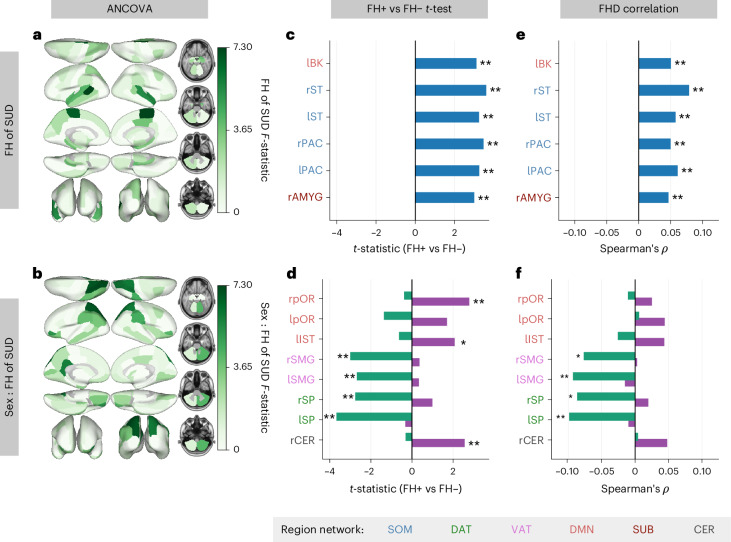
Regional TE differs by FH of SUD and its interaction with sex. **a**,**b**, ANCOVA models on mean regional TE (86 values per participant; total *N* = 1,611; 238 FH+ females, 626 FH− females, 198 FH+ males, 549 FH− males) revealed nominally significant effects of FH in six regions (**a**) and FH-by-sex interaction in eight regions (**b**), although none survived correction across all regions. Regional *F*-statistics are plotted on the brain regardless of significance. **c**, Unpaired *t*-tests showed greater TE in FH+ (*N* = 436) than FH− (*N* = 1,175) individuals across the six regions found to have an effect of FH of SUD in ANCOVA models. **d**, Unpaired *t*-tests comparing FH+ versus FH− participants within each sex for the TE of eight regions found to have a FH-by-sex interaction effect revealed higher TE in FH+ females (*N* = 238) than FH− females (*N* = 626) in the right pars orbitalis, left isthmus cingulate and right cerebellum, and lower TE in FH+ males (*N* = 198) than FH− males (*N* = 549) in the bilateral superior parietal lobules and supramarginal gyri. Purple denotes females and green denotes males. **e**, Spearman rank correlations revealed weak positive associations between FHD and TE in the six regions found to exhibit a main effect of FH of SUD across all participants (*N* = 1,886). **f**, Spearman rank correlations were performed within each sex between FHD and TE of the eight regions found to have an interaction effect of FH-by-sex, revealing weak negative associations in males (*N* = 885) in the bilateral superior parietal lobules and right supramarginal gyrus and no significant associations in females (*N* = 1,001). In **c**–**f**, region labels are color-coded by Yeo network assignment. *P < 0.05 before correction; **P_FDR_ < 0.05. All tests were two-sided. Multiple comparisons were controlled with Benjamini–Hochberg FDR (*q* = 0.05) across families of tests. Exact *P* values, test statistics (for example, *F*, *t*), degrees of freedom and effect sizes are reported in the figure or source data. l, left; r, right; AMYG, amygdala; BK, banks of the superior temporal sulcus; IST, isthmus cingulate; PAC, paracentral gyrus; pOR, pars orbitalis; SMG, supramarginal gyrus; SP, superior parietal lobule; ST, superior temporal gyrus. Source data

We next sought to identify regions contributing to the observed FH-by-sex differences in global and network TE. We ran two-way ANCOVAs on mean regional TE (dependent variable) using the same terms included in the models above. ANCOVA results for all regions are provided in Supplementary Information (Supplementary Figs. 8 and 9). Before FDR correction (over 86 regions), FH of SUD had a significant effect on the mean regional TE of the bilateral paracentral lobule, bilateral superior temporal gyrus, right banks of the superior temporal sulcus (STS) and right amygdala (Fig. 4a). Unpaired *t*-tests were performed to compare FH+ and FH− individuals’ mean regional TE for the six regions found to have a significant effect of FH or FH-by-sex. All regions analyzed exhibited significantly greater mean regional TE in FH+ individuals compared to FH− individuals (regardless of sex; Fig. 4b), and had significant, though weak, positive correlations with FHD (Fig. 4c). These six regions belong to the DMN, somatomotor network (SOM) and SUB networks.

The interaction between FH of SUD and sex had a significant effect on mean regional TE—prior to correction—bilaterally in the pars orbitalis, superior parietal lobule and supramarginal gyrus, and in the left isthmus cingulate and right cerebellum (Fig. 4d). Unpaired within-sex *t*-tests revealed significantly higher mean regional TE of the bilateral pars orbitalis, left isthmus cingulate and right cerebellum in FH+ females compared to FH− females. Mean regional TE of the bilateral superior parietal lobule and bilateral supramarginal gyrus was significantly lower in FH+ males compared to FH− males (Fig. 4e). The direction of correlations between FHD and mean regional TE of these regions largely recapitulated the results of within-sex *t*-tests. The bilateral supramarginal gyri and superior parietal lobules demonstrated significant, yet mild, negative correlations with FHD in males (Fig. 4f). Group differences in pairwise regional TE of these regions were consistent with the mean regional TE results shown here (Supplementary Figs. 4 and 5). The regions found to have lower mean TE in FH+ versus FH− males all belong to the DAT and VAT networks, and the regions found to have greater mean TE in FH+ versus FH− females all belong to the DMN and CER networks.

### Robustness analyses

To ensure the robustness of our results, we replicated our main findings in several ways: (1) re-clustering with *k* = 5 brain states, (2) utilizing individual SCs in a cortex-only parcellation and (3) in a cohort of sex, age and in-scanner motion-matched participants from an external dataset (the National Consortium on Alcohol and NeuroDevelopment in Adolescence, NCANDA)^89^. We also re-ran two-way ANCOVA models by stratifying our cohort in different ways: (1) a single site with the largest number of participants, (2) within each MRI scanner model and (3) within each income category.

We found that our main results were largely consistent when varying the number of clusters (*k* = 5) (Supplementary Fig. 13) and when using individual SCs (Supplementary Fig. 16). Our independent analysis of the NCANDA dataset showed similar trends of FH+ > FH− females and FH+ < FH− males at the global and regional levels for mean and pairwise TE, further supporting the generalizability of our findings across different populations (Supplementary Figs. 14 and 15). Analysis within the site with the greatest number of participants (site 16) displayed significant FH-by-sex interactions on mean global TE prior to correction, mean DMN TE after correction, and mean TE of the DAT and CER networks before correction (Supplementary Fig. 17). Analyzing the data by MRI scanner model, we observed that our main results were largely consistent in participants scanned with Siemens models, whereas results from the GE scanner differed, probably due to demographic differences and lower data quality (Supplementary Fig. 18). Supplementary Table 6 presents the participant demographics by MRI model. Previous ABCD analyses found GE scanners have lower reproducibility in dMRI metrics^90^, higher distinguishability after site-normalization in rsfMRI data^91^ and higher non-compliance to imaging protocols across modalities^92^, compared to Siemens scanners. Furthermore, Siemens scanners implement real-time motion monitoring but GE scanners do not^93^, and motion correction is critical in this dataset of young adolescents. When stratifying the cohort by income level, our primary findings were consistent mainly in the highest income group (that is, largest subgroup; Supplementary Fig. 20). Overall, these analyses confirm the robustness and reliability of our findings across various conditions and datasets, but indicate a possible influence of socioeconomic and demographic factors.

To further contextualize our findings, we examined associations between TE and behavioral and psychological risk factors for SUD in the ABCD cohort (Supplementary Section 12). Follow-up two-way ANCOVA and sex-stratified correlation analyses revealed that these relationships were often sex-specific. Overall, females exhibited modest positive correlations between TE and syndrome scales from the Childhood Behavior CheckList (CBCL)^94^, and males showed modest negative correlations with impulsivity-related traits from the Behavioral Inhibition/Behavioral Activation (BIS/BAS) and UPPS-P Impulsive Behavior (UPPS-P) scales^95,96^. In females, mean DMN TE was positively correlated with CBCL subscales for rule-breaking behavior, social problems and somatic complaints. In males, mean DAT and VAT TE values showed modest negative associations with BAS Drive and Fun-Seeking, as well as UPPS-P Positive Urgency. Further details on the interactions between behavior, sex and FH are provided in Supplementary Section 12.2). These results suggest that altered brain dynamics in at-risk youth may reflect both sex-specific and potentially sex-general neurobehavioral vulnerabilities relevant to future SUD outcomes.

## Discussion

Using an NCT framework, we modeled the brain as a networked dynamical system to examine how FH of SUD shapes brain activity dynamics in substance-naïve youth. Our findings demonstrate that FH of SUD manifests both through sex-independent increases in TE in specific regions and through divergent effects in males and females, with FH+ females exhibiting elevated DMN TE and FH+ males exhibiting reduced attentional network TE. These divergent findings in males and females with FH of SUD probably reflect sex-specific responses to genetic and environmental factors contributing to familial risk. The mechanism linking differences in TE at rest to SUD predisposition remains unclear. One possibility is that the energetic demand of a network or region determines its dominance in flexibly driving or suppressing brain-state transitions during rest, action and environmental processing. This theory, grounded in the principle of energy minimization^78,97^, suggests that networks requiring greater TE exert less efficient and flexible control over whole-brain dynamics. In this context, our findings imply that familial SUD reduces the flexible control of the DMN in females and disinhibits lower-order attentional networks in males, potentially predisposing each sex to distinct neurobehavioral pathways to SUD.

### Sex-independent elevation in regional TEs in FH+ youth

Across the sexes, FH+ youth showed elevated TE in the paracentral lobule, amygdala and superior temporal regions. These regions have been consistently linked to executive functioning, reward responsivity, craving and emotional processing in individuals with SUD^98–102^ and in FH+ youth^36,42,103–106^. Notably, the amygdala—a region strongly implicated in internalizing disorders—shows alterations that are more pronounced in FH+ females across development^38,104^, consistent with females’ greater tendency for internalizing pathways to SUD. Longitudinal studies are needed to determine whether regional TE trajectories differ by sex. Together, these findings suggest disruptions in these regions may represent shared familial markers of SUD risk.

### Inflexible DMN dynamics in FH+ females

FH+ females exhibited the highest mean global TE across groups, suggesting reduced neural flexibility and a greater tendency to become ‘stuck’ in certain brain states. This pattern resembles the elevated global TE we previously observed in young adults with heavy drinking^65^, and may help explain the accelerated habit formation reported in females^15,107^. Elevated TE was most pronounced in the DMN, a network widely implicated in SUD risk and FH of SUD^44,75,108,109^. Greater DMN TE may confer SUD vulnerability by promoting (1) persistence in internally oriented states, (2) disruption of rest-task transitions and (3) weakened top–down regulation of lower-order systems.

The DMN is a task-negative network, most active at rest and associated with self-referential and internally directed cognition^110^. FH+ females exhibited greater pairwise DMN TE for transitions to VIS+, VIS− and DMN− states, but not to the DMN+ state. This pattern suggests that once engaged in a negative internal state (for example, stress, withdrawal, craving), FH+ females may experience greater difficulty in disengaging. Supporting this, global and DMN TE correlated modestly with somatic symptoms—physical complaints that reflect underlying psychological distress (Supplementary Section 12.2)—indicating heightened sensitivity to negative internal states.

The DMN also guides rest-task transitions^111^, a process disrupted in FH+ youth^75^. Consistent with this, denser FH of SUD has been linked to DMN hypoactivity during task and hyperactivity during rest^44^. Elevated DMN TE in FH+ females may therefore reflect reduced flexibility in shifting between internal and external states, thereby weakening inhibitory control over goal-directed behavior, heightening vulnerability to rumination and stress reactivity, and biasing behavior toward negative reinforcement^15,112^.

Positioned at the top of the network hierarchy, the DMN is thought to exert inhibitory regulation over attention and sensory systems^113–115^. Elevated DMN TE during transitions to VIS+/− may therefore signal inefficient top–down control^116,117^. This aligns with evidence that deficient DMN modulation in FH+ youth predicts impaired inhibition, reduced cognitive efficiency and poorer goal-directed behavior^44^, and evidence that DMN inefficiency mediates the relationship between drug use, inhibitory deficits and disrupted sequential planning in adolescents^118^. Such inefficiency may explain the correlations observed here between DMN TE and rule-breaking behavior in females (Supplementary Section 12.2). Regionally, elevated TE was observed in the pars orbitalis and isthmus cingulate, with the latter also showing higher TE in females in an external dataset (Supplementary Section 10.2). Both regions have been implicated in inhibitory deficits in FH+ youth^42,119–121^. Elevated TE in the cerebellum, a DMN-coupled region with atypical inhibitory function in FH+ youth^122,123^, further reinforces this interpretation. Notably, reduced efficiency of posterior cingulate-cerebellar circuits has been reported in alcoholism^109^. These findings suggest that elevated TE in these regions probably represents premorbid alterations in the neural efficiency of inhibitory control, emerging before substance use and conferring vulnerability to its onset.

Taken together, these findings suggest that FH+ females may be predisposed to SUD through DMN inflexibility that disrupts transitions between internal and external states and weakens inhibitory control. This inefficiency may heighten sensitivity to negative internal states such as rumination and stress, channeling risk along an internalizing pathway.

### Disinhibited attentional dynamics in FH+ males

FH of SUD manifests in males in the opposite direction, with lower global TE suggesting overall disinhibition of brain dynamics. The largest TE differences in FH+ males were localized to attentional networks: the DAT, which supports goal-directed attention, and the VAT, which mediates bottom–up reorienting to salient stimuli^124^. These findings align with evidence linking attention deficits to elevated SUD risk in youth^125^, abnormal functional activity of attention networks in individuals with SUD^126–129^, and a sustained attention network predictive of future substance use in adolescents^58^. Furthermore, reduced P300 amplitude—a neural marker of disinhibition tied to inefficient attentional allocation—has been consistently observed in males, but not females, with SUD and with FH of SUD^130^. Specifically, FH+ males showed reductions in pairwise VAT TE for bottom–up transitions to the DMN meta-state and in DAT TE for transitions to both the DMN and VIS meta-states. Reduced energetic demands in attentional networks may promote disinhibition by lowering the threshold for cue reactivity and reward-driven attention. Together, these alterations point to heightened sensitivity to external stimuli, potentially manifesting as greater responsivity to drug-related cues and reward-directed attention. Thus, reduced energetic demands in attentional networks before substance exposure may predispose FH+ males to more readily attend to the rewarding effects of substances once exposed. This interpretation is supported by the modest negative correlations we observed between DAT/VAT TE and behavioral measures, including positive urgency, lack of planning, fun-seeking and goal-driven behavior (Supplementary Section 12.2).

Regionally, FH+ males exhibited lower TE bilaterally in the superior parietal lobules and supramarginal gyri—regions implicated in drug cue reactivity in SUD^131^. A systematic review has identified the parietal cortex, which supports goal-directed attention^132^, as the most common region to show sex differences in SUD in rsfMRI studies^26^. The left superior parietal lobule is hyperactive in individuals with SUD^98^ and during attentional control tasks in FH+ youth^133^, and its functional connectivity exhibits protracted neurodevelopment in alcohol use disorder (AUD) FH+ youth^134^. Moreover, reduced cortical thickness of the left supramarginal gyrus was the strongest predictor of future alcohol use in substance-naïve youth, second only to male sex^135^. Together, regional reductions in TE in FH+ males appear to reflect overactive reward salience processing and attentional disinhibition.

### Sex-divergent neural pathways of familial SUD risk

Our results suggest that FH+ males show stronger reward salience driven by low-cost attentional dynamics, whereas FH+ females exhibit high-cost DMN dynamics that could impair inhibitory control. These findings align with previous reports that females exhibit ‘stickier’ brain dynamics, marked by fewer state switches and slower response inhibition, whereas males show greater dynamic fluidity, shifting between states more frequently and exploring a larger state space^71^. We build on two existing models of SUD risk: dual-systems theory of adolescent vulnerability and sex-divergent substance reinforcement.

**Fig. 5:**
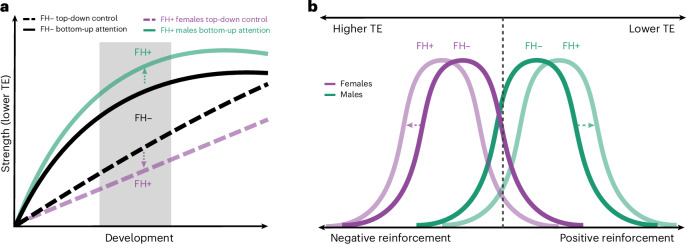
Sex-specific models of FH effects on adolescent SUD vulnerability. **a**, Dual-systems models implicate a developmental imbalance between top–down inhibitory control and bottom–up reward saliency in FH− adolescents (black lines), with baseline sex differences not depicted. FH of SUD appears to amplify this imbalance in sex-specific ways: FH+ females (purple dashed line) show protracted development of top–down control, linked to higher DMN TE, whereas FH+ males (green solid line) show accelerated development of bottom–up attention, linked to lower DAT/VAT TE. The gray-shaded area marks the age range of the present cohort. Longitudinal data are needed to confirm these trajectories. Although FH+ males and females may share vulnerability to both processes, these effects appear more prominent within the respective sex shown here. **b**, We propose that sex-divergent reinforcement mechanisms observed in SUD (dark lines) emerge in adolescence and are further amplified by FH of SUD (light lines). Higher TE in FH+ females (purple) may reflect neural inflexibility, promoting negative reinforcement via difficulty disengaging from negative internal states. Lower TE in FH+ males (green) may reflect neural disinhibition and a stronger susceptibility to positive reinforcement of external stimuli. As depicted here, these populations overlap—some females may be more prone to positive reinforcement, and some males to negative reinforcement of substance use. Panel **b** adapted from ref. ^16^ under a Creative Commons licence CC BY-NC-ND 4.0.

First, dual-systems theory attributes SUD risk to an imbalance between heightened bottom–up salience and weakened top–down control, but is typically described as sex-invariant^136^. Our data suggest that FH+ males and females map onto distinct halves of this model: higher TE may impair the DMN’s ability to exert inhibitory control in FH+ females, whereas lower TE in the DAT/VAT may amplify reward salience in FH+ males (Fig. 5a). Longitudinal work is needed to establish sex-specific developmental trajectories. By analogy, FH+ females appear less able to ‘step on the brakes’ (higher DMN TE), whereas FH+ males more readily ‘step on the gas’ (lower DAT TE), both alterations potentially accelerating progression to SUD. This echoes clinical evidence that males are more likely to initiate use earlier, whereas females progress more rapidly to loss of control once initiated^15,107^. Importantly, the DMN and DAT are strongly anticorrelated from infancy^115,137–140^, suggesting that opposite alterations in these networks may nonetheless converge on equifinal behavioral phenotypes of heightened SUD risk.

Second, sex-specificity in substance reinforcement (that is, stronger positive and negative reinforcement in males and females, respectively) has been thought to emerge in late adolescence or young adulthood^16,107^. Our findings suggest that this divergence is already evident by ages 9–11 years and is amplified by FH (Fig. 5b). Greater DMN TE in FH+ females may explain heightened vulnerability to an internalizing pathway to SUD via negative reinforcement, whereas lower TE in attentional networks may bias FH+ males toward an externalizing pathway via positive reinforcement. Unexpectedly, in females, global and DMN TE correlated with externalizing rather than internalizing symptoms (Supplementary Section 12.2), a pattern that probably reflects developmental stage, as internalizing symptoms typically emerge in girls by mid-adolescence^141^. Thus, greater TE in females may index a latent neural predisposition to both internalizing disorders and SUD, a vulnerability that may become more apparent as the cohort matures.

Taken together, our findings suggest that familial risk manifests as a dual-systems imbalance in both sexes, through opposite mechanisms that foreshadow adult reinforcement pathways and may widen the gap between sexes. Such sex-divergent effects—in which the same phenotype maps onto opposite neural manifestations—are well documented in SUD and SUD risk. For example, externalizing problems have been linked to DMN–FPN hyperconnectivity in males but DMN-affective hypoconnectivity in females^142^, and greater prefrontal activity predicts lower stress reactivity in men but higher in women^112^. Additionally, the networks most implicated here (that is, DAT, VAT and DMN) are also those in which baseline sex differences have been most consistently reported^143–145^.

Specific mechanisms underlying this sex difference are not yet fully understood, but may include hormonal modulation of dopaminergic and fronto-striatal circuits, genetic and epigenetic regulation of reward pathways, and sex-specific neurodevelopmental responses to stressors and sociocultural factors^14,146,147^. The historical neglect of women and people assigned female at birth, and the failure to account for sex as a moderator, may partly explain the mixed or null findings often reported in the familial risk literature^148^. Without modeling the interaction between sex and FH, opposing effects in males and females cancel out. We urge future work to treat sex and gender as moderators of familial risk to uncover these hidden mechanisms and to advance precision prevention and intervention strategies tailored to at-risk youth.

### Cortical functional dynamics as early markers of SUD vulnerability

Most alterations linked to FH of SUD were cortical, with the exception of the cerebellum and amygdala. This contrasts with previous work that emphasized subcortical dopaminergic systems in SUD risk^10,149–152^. A meta-analysis has identified striatal differences as the most consistent marker of vulnerability, but largely in older adolescents who had already initiated use^10^. Subcortical alterations may therefore emerge later in development or following chronic exposure^149,153,154^. Indeed, addiction is characterized by both cortical and subcortical pathologies, whereas occasional use is marked by cortical dysfunction alone^48^. Together, these findings suggest familial SUD risk manifests first in cortical networks, with subcortical abnormalities emerging later in adolescence or after exposure.

Structural findings in FH+ youth have been inconsistent. We previously reported higher TE in subcortical–frontoparietal transitions in young adults with heavy alcohol use that mapped to structural abnormalities^65^. By contrast, here we identify functional cortical alterations in substance-naïve FH+ youth, robust across both group-average and individual SCs (Supplementary Fig. 16). Although some studies report structural connectivity alterations in FH+ adolescents^61^, others find no differences in white-matter integrity^43^. Our findings align with the latter, and suggest that functional cortical abnormalities precede substance initiation, with cortical and subcortical structural pathologies more likely accumulating after chronic use.

### Limitations and future directions

Several limitations should be noted. The age range of our cohort coincides with a period of major neurodevelopmental change, which varies by sex^81,125,155–158^ and by interactions between FH and sex^32,38^. The young age of the cohort also limited pubertal-stage diversity, constraining insights into how FH, sex and puberty interact^141^. Thus, our findings should be validated and extended across the full developmental window. Notably, we partially replicated our results in the NCANDA dataset among individuals aged 12–16 years. The reported effect sizes fall in ranges traditionally considered small. However, small effects in large population-based samples are often reliable and reproducible^159–162^. Moreover, effect size may be underestimated here by the exclusion of participants with excessive head motion, a heritable trait linked to impulsivity and future alcohol use^163–166^.

The majority of FH+ participants had relatives with alcohol use problems, making our findings more representative of FH of AUD. Whether cross-substance effects generalize remains unclear. In addition, the present work does not disentangle whether findings in FH+ children reflect genetic predisposition^7,167^, adverse childhood experiences associated with having a family member with SUD^8^, prenatal substance exposure, or their combination. Future work should aim to separate these influences.

Importantly, interpretation of sex differences is limited by a reliance on a binarized variable of sex assigned at birth and by not accounting for gender identity due to limited gender diversity in the cohort. Sex and gender are not binary, but are complex, multidimensional constructs with distinct neural manifestations^17,19^, and ‘male’ and ‘female’ features exist as a mosaic in all brains^81,168^. Although biological sex is a practical biomarker that revealed dimorphic traits in relation to FH of SUD, these differences may partly reflect variables covarying with sex rather than true dimorphisms^81,169^. Thus, sex is an informative but imperfect proxy. Future research should test whether these traits vary across diverse sex and gender identities and examine how sex, gender and sexual orientation intersect with SUD risk, particularly given elevated rates among LGBTQ+ youth^170–172^.

## Conclusion

Our study reveals sex-specific effects of FH of SUD, with distinct network alterations in male and female youth: FH+ males exhibited lower TE in attentional networks, whereas FH+ females showed heightened TE in the DMN. This pattern may translate to FH+ males more readily ‘stepping on the gas’ and FH+ females having greater difficulty ‘stepping on the brakes’ in substance-use trajectories. These findings suggest that mechanisms underlying SUD predisposition are shaped by sex-specific neurodevelopmental pathways that may converge on similar behavioral outcomes. Our results validate previous reports of sex-related differences in familial risk and provide novel evidence that the neural substrates of sex-divergent substance-use behaviors observed in adults emerge in adolescence. By linking these alterations to behavioral measures, future substance use and replication in an external dataset spanning a wider age range, we strengthen the generalizability of our findings. Recognizing these mechanistic differences is essential for understanding SUD onset and developing targeted, sex-informed intervention strategies.

## Methods

### Sample characteristics

The Adolescent Brain Cognitive Development (ABCD) Study is longitudinally tracking the brain development and health of a nationally representative sample of children aged 9–11 years (at the time of enrollment) from 21 centers across the United States (https://abcdstudy.org). All parents or legal guardians provided written informed consent before participation in the study, and children provided verbal assent. Participants and their families received financial compensation for their time. Research protocols were approved by the institutional review board of the University of California, San Diego (no. 160091), and the institutional review boards of the 21 data-collection sites^173^.

The current study utilized neuroimaging data from the 2.0.1 release and non-imaging instruments from the baseline assessment updated to ABCD Data Release 5.1. Access to ABCD Study data is restricted to protect participants’ privacy. Users must create an account through the National Institute of Mental Health Data Archive and they may then complete the necessary steps to gain access. Researchers with access to the ABCD data will be able to download the data from https://nda.nih.gov/study.html?id=1368.

#### Exclusions

From the original ABCD cohort of *N* = 11,868, we excluded youth who (1) did not survive strict MRI quality control and/or exclusion criteria previously established by refs. ^174,175^ (*N* = 9,506), (2) were scanned on Phillips scanners (*N* = 2), (3) did not meet criteria for group definitions of FH+ or FH− (‘Exclusions’ section; *N* = 109), (4) had missing information on maternal substance use (‘Exposure to substances’ section; *N* = 59), (5) were adopted (*N* = 9), (6) had previously used substances (‘Exposure to substances’ section; *N* = 54), (7) had a mismatch between reported sex assigned at birth and their sex determined by salivary samples (*N* = 17), (8) had missing household income information (‘Household income and parental education’ section, *N* = 75), (9) had missing information on parental mental health issues (‘FH of SUD and mental illness’ section, *N* = 79) or (10) had missing information on pubertal status (‘Pubertal status’ section, *N* = 14). A further *N* = 58 participants were excluded (after *k*-means clustering and TE calculations) due to outlier mean global TE values (‘Defining outlier transition energies’ section). Our final cohort had *N* = 1,886 participants. Supplementary Table 1 provides information on excluded participant demographics.

#### FH of SUD and mental illness

We used the baseline Family History Module Screener (FHAM-S)^176,177^, in which parents reported substance use and psychopathology among first- and second-degree biological relatives. In the FHAM-S, drug or alcohol problems may include marital, work, school, legal (for example, DUI), health, rehabilitation, heavy use or social issues.

Following previous work^32,54,83^, participants were classified as FH+ if they had ≥1 parent or ≥2 grandparents with a history of SUD; FH− if no parents or grandparents had SUD; and FH+/− if they had one grandparent with SUD. FH+/− individuals, presumed to have minimal genetic load^32^, were excluded from categorical analyses, but included in continuous FHD analyses. FHD was computed as the sum of substance-related problems in biological parents (+1 each) and grandparents (+0.5 each), ranging from 0 (no history) to 4 (SUD in both parents and all four grandparents)^54^.

Alcohol and drug histories were reported separately. We utilized a cross-substance definition of FH+ to capture shared heritable vulnerability^7^ and brain network abnormalities^178^ across various SUDs.

To account for psychiatric comorbidity, we also included a binary variable indicating parental history of mental illness other than SUD. Per FHAM-S, this includes suicide, depression, mania, antisocial personality, schizophrenia and other emotional or mental health issues. Participants met criteria if ≥1 parent had any such condition.

#### Exposure to substances

For childhood substance use, to isolate the effects of FH of SUD from substance use itself, we excluded youth who had initiated substance use according to parents or children themselves^179^. Participants were excluded if they self-reported lifetime use of more than a sip of alcohol, more than a puff of a cigarette/e-cigarettes or any use of nicotine products, cannabis products, synthetic cannabinoids, cocaine, cathinones, methamphetamine, ecstasy/MDMA, ketamine, gamma-hydroxybutyrate, heroin, psilocybin, salvia, other hallucinogens, anabolic steroids, inhalants or prescription misuse of stimulants, sedatives, opioid pain relievers or over-the-counter cough/cold medicine (*N* = 39). Parents were also asked about their child’s substance use, including alcohol (consumed three or more drinks a day, consumed two drinks in the last 12 months) or used drugs (cocaine, marijuana, solvents, stimulants, tobacco, opioids, hallucinogens, sedatives or other). If parents endorsed any of these, children were excluded from analyses (*N* = 15).

Given the known impact of maternal substance use during pregnancy on brain development^180,181^, we included prenatal substance exposure as a binary variable in our ANCOVA models. Prenatal substance exposure was reported based on caregiver recall in the ABCD Developmental History Questionnaire^177^. Consistent with previous work using dichotomous analyses^182^, we considered prenatal exposure as either present or absent based on whether mothers reported maternal use of alcohol or other drugs after the pregnancy was recognized. Additional details on the ABCD protocol and a table of the breakdown of substance type by FH of SUD group are provided in Supplementary Section 2.

#### Household income and parental education

We included two key socioeconomic indicators: household income (HI) and parental education (PE), both critical to mental health research per NIMH guidelines^183^ and linked to substance use outcomes^184,185^. In the ABCD Study, HI and PE were parent-reported via demographic questionnaires^177^. To simplify modeling while retaining detail, we followed previous work in re-coding these variables^186,187^. PE responses (originally 21 options) were collapsed into five categories: high school, high school/GED, some college, associate’s/bachelor’s and postgraduate degree. The higher value was used if both caregivers provided data; otherwise, we used the available response. HI (originally nine levels) was collapsed into three levels: (1) less than US$50,000, (2) more than US$50,000 and less than US$100,000 and (3) over US$100,000. If only one caregiver reported HI, that value was used.

#### Sex assigned at birth

In our analyses, we utilized a binary measure of sex assigned at birth, which we refer to as ‘sex’. We did not control for or assess gender-based differences due to limited gender diversity in this cohort at baseline. We excluded individuals whose sex assigned at birth did not match their sex as determined in a salivary sample, as this could indicate clerical errors in reporting or reflect sex- and gender-diverse individuals, for whom we did not have a large enough population in this cohort to properly assess.

#### Pubertal status

We used parent-reported Pubertal Development Scale (PDS) summary scores from the baseline visit^188^, as youth tend to overestimate their development at younger ages^189^. When parent reports were missing (*N* = 30), child-reported scores were used. PDS questions are sex-specific and were summarized into a five-level categorical stage: (1) pre-pubertal, (2) early pubertal, (3) mid-pubertal, (4) late pubertal and (5) post-pubertal. Due to small sample sizes in higher stages (for example, only one male in stage > 3), we collapsed stages 3, 4 and 5 into a single level, resulting in three modified stages: 1 = pre-pubertal, 2 = early pubertal, 3 = mid to post-pubertal. Females generally had more advanced pubertal stages than males, and FH+ youth of both sexes showed more advanced development than FH− youth. The modified PDS stage and its interaction with sex were included in all ANCOVA models.

### Neuroimaging data

#### Parcellation

In our main results, we present analyses in which the rsfMRI time series and group-average SCs were parcellated using an 86-region atlas derived from FreeSurfer (FS86) combining the 68 region Desikan–Killiany (DK68) gyral atlas (34 cortical regions per hemisphere) with 16 subcortical structures (eight per hemisphere, excluding brainstem) and two cerebellar structures (one per hemisphere) to render a whole-brain anatomically defined parcellation for each participant^190,191^. Each cortical region was assigned to one of seven networks of the functionally defined Yeo 7-network parcellation^87^. Subcortical regions were assigned to a subcortical network and cerebellar regions to a cerebellar network. The rsfMRI time series and individual SCs were also extracted in the DK68 cortical atlas (no subcortical region data).

#### rsfMRI

We analyzed baseline rsfMRI data from the ABCD Study, using minimally processed scans further preprocessed and quality-controlled as described in refs. ^174,175^. Philips scanner data were excluded due to known post-processing errors per ABCD recommendations. Preprocessing included removal of initial frames, alignment to T1 images via boundary-based registration (BBR), and censoring of volumes with framewise displacement >0.3 mm or DVARS >50, plus one preceding and two following volumes. Uncensored segments <5 volumes were also censored. Runs were excluded if more than 50% of volumes were censored or BBR cost exceeded 0.6.

Nuisance covariates (global signal, motion parameters, ventricular and white-matter signals, and their derivatives) were regressed out using non-censored volumes. Data were bandpass-filtered (0.009 ≤ *f* ≤ 0.08 Hz), mapped to FreeSurfer fsaverage6 space, and smoothed using a 6-mm full-width at half-maximum kernel. We then removed censored volumes and normalized BOLD time series by mean gray-matter signal (pre-filtering). After censoring, an average of 1,158.3 ± 289.18 (mean ± s.d.) rsfMRI frames remained per scan.

To account for scanner effects^91^, scanner model was included as a covariate in all ANCOVA models. Only Siemens Prisma, Siemens Prisma Fit and GE Discovery MR750 scanners were included.

#### SCs

In the ABCD Study, dMRI data were collected at baseline assessment^192^. The preprocessed dMRI data were further processed by deterministic tractography with SIFT2 global streamline weighting and regional volume normalization. The SCs were extracted in FS86 (cortical and subcortical) for 149 participants and in DK68 (cortical only) for 2,080 participants. The SC matrices are symmetric, with the diagonal (self-connections) set equal to zero. Mean global TE values using a group-average SC and individual SCs for *N* participants were found to be highly correlated (Pearson’s *ρ* = 0.998, *P* < 0.0001). Given this high correlation and the known relevance of subcortical regions in the SUD literature^10,149–152^, we chose to use the group-average SC for the FS86 atlas. This choice of a group-average SC is further supported by previous NCT work^80^ and recent observations of a lack of differences in white-matter integrity between FH+ and FH− drug-naïve adolescents, suggesting that familial predisposition is manifested primarily in functional dynamics^43,60^. The main results were replicated in the DK68 parcellation using individual, cortex-only SCs (Supplementary Fig. 16).

#### Framewise displacement

Given the concern for head motion during neuroimaging of a pediatric cohort, we controlled for participants’ tendency to move their head during rest in the scanner by calculating the average framewise displacement (FD) across all time points for each participant. We include mean FD as a covariate of no interest in all ANCOVA models.

### NCT analyses

#### Extraction of brain states

Following ref. ^84^, all participants’ fMRI time series were concatenated in time, and *k*-means clustering was applied to identify clusters of brain activation patterns, or brain states. Pearson correlation was used as the distance metric, and clustering was repeated ten times with random initializations before choosing the solution with the best separation of data. To further assess the stability of clustering and ensure our partitions were reliable, we independently repeated this process ten times and compared the adjusted mutual information (AMI) between each of the ten resulting partitions. The partition that shared the greatest total AMI with all other partitions was selected for further analysis. In general, we found that the mutual information shared between partitions was very high (>0.99), suggesting consistent clustering across independent runs. We chose the number of clusters *k* via the elbow criterion, that is, by plotting the gain in explained variance across clusterings for *k* = 2 through *k* = 14 and identifying the ‘elbow’ of the plot, which was at *k* = 4 (Supplementary Fig. 1). In addition, *k* = 4 fell below 1% of variance explained by clustering, a threshold used previously for determining *k* (refs. ^84,85^). We thus chose *k* = 4 for its straightforward and symmetric interpretation, and replicated the main results for *k* = 5, as shown in Supplementary Information (Supplementary Section 10.1). For interpretability, each cluster centroid was named via one of nine a priori defined canonical resting-state networks (RSNs)^87^ plus subcortical and cerebellar networks by the cosine similarity between the centroid and binary representations of each RSN. Because the mean signal from each scan’s BOLD time series was removed during bandpass filtering, positive values in the centroid reflect activation above the mean (high-amplitude) and negative values reflect activation below the mean (low-amplitude). Individual brain-state centroids were calculated for each individual across all included time points of their rsfMRI scans.

#### TE calculations

To calculate the TEs we followed procedures similar to those described elsewhere^84,85^, and summarize them briefly here. We employed a linear time-invariant model:

$${\dot{x}{(t)}={Ax(t)+Bu(t)}}$$

where *A* is a representative (group average) *N* *×* *N* structural connectivity matrix obtained as described above using deterministic tractography from a subset of ABCD participants (‘SCs’ section). *A* is normalized by its maximum eigenvalue plus 1 and subtracted by the identity matrix to create a continuous system. *x*(*t*) is a vector of length *N* containing the regional activation at time *t*. *B* is an *N* *×* *N* matrix of control points, in this case, the identity matrix is used for uniform control. *u*(*t*) is the external input into the system. *N* is the number of regions in our parcellation, where *N* = 86 for our main results. We selected a time horizon of *T* = 1.501, which yielded the strongest inverse relationship between TE and transition probability (that is, the likelihood of a transition to occur), across the tested range (*T* = 0.001 to 10), consistent with previous work^84,85^. To compute the minimum control energy required to drive the system from an initial brain state to a target state over *T*, we computed an invertible controllability Gramian for controlling network A from *N* nodes.

Using the above methodology to define brain states (‘Extraction of brain states’ section), we calculated the regional TE for a given transition between each pair of brain states and persistence within each state (pairwise regional TE). To calculate pairwise regional TEs, we integrated *u*(*t*) over the time horizon to yield the total amount of input signals into each region necessary to complete each transition, resulting in a *k* × *k* matrix for each region (where *k* = 4 in our main results). We calculated the global TE (pairwise global TE), a *k* × *k* matrix, by summing all pairwise regional TEs for the transition. We calculated the TE required of a network (pairwise network TE) by summing all pairwise regional TEs for those regions assigned to a network, resulting in a *k* × *k* matrix for each network. To calculate mean global TE (1 constant), mean network TE (vector of length equal to the number of networks) and mean regional TE (vector of length equal to number of regions), we averaged across all pairwise TEs at each respective level of analysis.

#### Defining outlier transition energies

After calculating mean global TE values, we excluded 58 individuals from further analyses due to outlier mean global TE values. Outliers were defined as participants with mean global TE values exceeding 1.5 times the IQR above the upper quartile (75th percentile) or below the lower quartile (25th percentile). The excluded participants exhibited significantly higher mean framewise displacement compared to non-outlier participants (*t* = 3.14, *P* = 0.0017). Sample characteristics of all excluded participants are provided in Supplementary Table 1.

### Statistics

We tested for FH+ versus FH− group differences at various levels of TE using two-way ANCOVA models, with sex assigned at birth and *F* of SUD as the primary independent variables, and their interaction (sex × FH) as the effect of interest. Covariates included age, race/ethnicity, parental education, household income, prenatal substance exposure, parental mental health, MRI scanner model, in-scanner motion (mean framewise displacement) and pubertal stage. We also examined two additional interaction terms (FH × household income and sex × puberty). Full ANCOVA results are reported in Supplementary Section 8. Effect sizes for all model terms were summarized using partial *η*^2^. Additionally, for TE measures with significant ANCOVA effects, post hoc tests were conducted using unpaired, two-sided *t*-tests to determine the direction of effect between subgroups: FH+ versus FH− across all participants (for FH main effects) and FH+ versus FH− within each sex separately (for sex × FH interactions). Cohen’s *d* was reported as the effect size for post hoc *t*-tests. Associations between mean global TE and FHD were further assessed using Spearman’s rank correlations. Multiple comparisons were corrected using the Benjamini–Hochberg FDR procedure (*q* = 0.05)^88^.

Custom scripts were developed using MATLAB R2023a and Python 3.11. MATLAB visualizations were generated using the gramm toolbox (v2.25; https://github.com/piermorel/gramm). Python visualizations were rendered using brainmontageplot (v1.4.2; https://github.com/kjamison/brainmontageplot).

### Reporting summary

Further information on research design is available in the Nature Portfolio Reporting Summary linked to this article.

## Supplementary information

Supplementary Information Supplementary Sections 1–12, including discussions, figures and tables, Figs. 1–25 and Tables 1–6.

Reporting Summary

## Source data

Source Data Figs. 2–4 Statistical source data.

## Data Availability

The data used in this study are from the Adolescent Brain Cognitive Development (ABCD) Study (https://abcdstudy.org), a longitudinal study supported by the NIH and other federal partners. Access to the ABCD data is subject to controlled access and requires registration through the National Institute of Mental Health Data Archive (NDA): https://nda.nih.gov. Researchers with approved access can download the raw and processed data used in this study. The ABCD neuroimaging data were originally downloaded from 10.15154/1504041 and preprocessed derivatives (as described in Chen et al.^174^ and Ooi et al.^175^) were utilized in the present study, available at https://nda.nih.gov/study.html?id=1368. Non-imaging data are from the ABCD 5.1 release (10.15154/z563-zd24). Collection and distribution of the NCANDA data were supported by NIH funding (AA021681, AA021690, AA021691, AA021692, AA021695, AA021696 and AA021697). Researchers with access to the NCANDA data will be able to download the data via https://nda.nih.gov/study.html?id=4513. Source data are provided with this paper.

## Code availability

This project utilized custom scripts as well as publicly available code published by Cornblath et al.^84^ and Singleton and colleagues^85^. All analysis code is available on GitHub at https://github.com/louisaschill/abcd_fhsud_nct.
