# Supplementary material for: Sex-specific differences in brain activity dynamics of youth with a family history of substance use disorder
Source: Nat Ment Health. 2025 Nov 21;3(12):1493–511. doi: 10.1038/s44220-025-00523-2 (PMC12705459; doi:10.1038/s44220-025-00523-2)
Supplement: Supplementary file 1 — Supplementary Sections 1–12, including discussions, figures and tables, Figs. 1–25 and Tables 1–6. [file 44220_2025_523_MOESM1_ESM.pdf]

nature mental health

Supplementary information

https://doi.org/10.1038/s44220-025-00523-2

Sex-specific differences in brain activity dynamics of youth with a family history of substance use disorder

In the format provided by the authors and unedited

Supplementary Information

S1 Subject exclusions

We excluded subjects who met any of the following criteria:

1. Failed MRI quality control and/or did not meet previously established exclusion criteria[1,2] (N=9506);
2. Were scanned on Philips scanners (N=2);
3. Did not meet criteria for family history (FH) group definitions of FH+, FH+/–, or FH– (N=109);
4. Had missing information on maternal substance use (N=59);
5. Were adopted (N=9);
6. Reported previous substance use (N=54);
7. Had a mismatch between reported sex and genetically determined sex from salivary samples (N=17);
8. Had missing household income information (N=75);
9. Had missing information on parental mental health (N=79);
10. Had missing information on pubertal status (N=14).

After these exclusions, we ran *k*-means clustering and calculated mean global transition energy (TE) for all remaining subjects. Subjects identified as outliers based on mean global TE values following *k*-means clustering and TE calculations were subsequently excluded (N=58). See Methods for more information on outlier criteria. Thus, our final sample size was N = 1886. See Table S1 below for more information on excluded subjects.

	Cohort with usable rsfMRI Data (N=2362)	Excluded Subjects (N=476)	Included Subjects (N=1886)
**Biological Sex, n (%)**			
Female	1265 (54%)	264 (55%)	885 (47%)
Male	1096 (46%)	211 (44%)	1001 (53%)
**Age in months, Mean ± SD**			
	119.9 ± 7.50	119.9 ± 7.63	120.2 ± 7.47
**Family History of SUD, n (%)**			
FH+	534 (23%)	98 (21%)	451 (24%)
FH-	1400 (59%)	225 (47%)	1169 (62%)
FH+/-	319 (14%)	44 (9%)	274 (14%)
Missing	109 (5%)	109 (23%)	0 (0%)
**Family History Density, Mean ± SD**			
	0.41 ± 0.67	0.44 ± 0.79	0.40 ± 0.64
**Framewise Displacement, Mean ± SD**			
	0.12 ± 0.08	0.13 ± 0.08	0.12 ± 0.08
**MRI Scanner Model, n (%)**			
GE Discovery MR750	670 (28%)	146 (30%)	524 (28%)
Siemens Prisma	823 (35%)	132 (28%)	691 (37%)
Siemens Prisma Fit	867 (37%)	196 (41%)	671 (36%)
Undefined	2 (<1%)	2 (<1%)	0 (0%)
**Household Income, n (%)**			
< $50,000	500 (21%)	99 (21%)	426 (23%)
$50,000–$100,000	666 (28%)	111 (23%)	563 (30%)
$100,000+	1196 (51%)	168 (35%)	897 (48%)
Missing	87 (4%)	98 (21%)	0 (0%)
**Parent Education, n (%)**			
< High School	68 (3%)	19 (4%)	49 (3%)
High School/GED	160 (7%)	35 (7%)	125 (7%)
Some College	251 (11%)	67 (14%)	184 (10%)
Associates/Bachelor	945 (40%)	180 (38%)	765 (41%)
Postgraduate	936 (40%)	173 (36%)	763 (40%)
Missing	2 (<1%)	2 (<1%)	0 (0%)
**Race/Ethnicity, n (%)**			
White	1412 (60%)	229 (48%)	1183 (63%)
Black	203 (9%)	59 (12%)	144 (8%)
Hispanic/Latinx	447 (18%)	102 (21%)	345 (18%)
Asian	58 (3%)	20 (4%)	38 (2%)
Other	241 (10%)	65 (14%)	176 (9%)
Missing	1 (<1%)	1 (<1%)	0 (0%)
**Prenatal Substance Exposure, n (%)**			
Yes	169 (7%)	47 (10%)	122 (6%)
No	2193 (93%)	429 (90%)	1764 (94%)
**Parent Mental Health, n (%)**			
Yes	1208 (51%)	224 (47%)	984 (52%)
No	1034 (44%)	132 (28%)	902 (48%)
Missing	58 (5%)	20 (4%)	0 (0%)

Table S1. Demographic and characteristic data for the full cohort of subjects for whom we had usable rsfMRI data[1,2], excluded subjects (i.e., those with missing data or outlier mean global TE values), and included subjects in our analyses. Excluded subjects are missing data for some variables. Values are n (%) unless otherwise specified; continuous variables are presented as mean ± SD.

S2 Prenatal substance exposure.

We included a binary variable of prenatal substance exposure in all ANCOVA models in the main analysis. Consistent with previous work using dichotomous analyses[3], we considered exposure as either present or absent based on whether there was reported maternal use of alcohol, tobacco, marijuana, cocaine/crack, heroin/morphine, oxycontin, or other drugs after pregnancy had been recognized. This classification was based on responses to the following items:

- *devhx_9_alcohol*: Once the biological mother/you knew she was pregnant, was she/you using alcohol?
- *devhx_9_marijuana*: Once the biological mother/you knew she was pregnant, was she/you using marijuana?
- *devhx_9_her_coc_crack*: Once the biological mother/you knew she was pregnant, was she/you using cocaine/crack?
- *devhx_9_her_tobacco*: Once the biological mother/you knew she was pregnant, was she/you using tobacco?
- *devhx_9_her_morph*: Once the biological mother/you knew she was pregnant, was she/you using heroin/morphine?
- *devhx_9_oxycont*: Once the biological mother/you knew she was pregnant, was she/you using oxycontin?
- *devhx_9_other_drugs*: Once the biological mother/you knew she was pregnant, was she/you using any other drugs?
- *devhx_9_other1_name_2, devhx_9_other2_name_2, devhx_9_other3_name_2, devhx_9_other4_name_2, devhx_9_other5_name_2* (up to 5 selections for other drugs used):
  - 0 = None
  - 1 = Amphetamines or methamphetamine (meth)
  - 2 = Benzodiazepines
  - 3 = Caffeine
  - 4 = Cathinones (bath salts)
  - 5 = Fake or synthetic marijuana (like spice or K2)
  - 6 = GHB (liquid G or Georgia home boy)
  - 7 = Hallucinogens (LSD or acid)
  - 8 = Inhalants
  - 9 = Ketamine (special K)
  - 10 = MDMA (ecstasy)
  - 11 = Opioids
  - 12 = Other
  - 13 = Barbiturates
  - 999 = Don't Know

If any of the above items were endorsed, the subject was considered to have prenatal substance exposure, and if responses to all items were negative, the subject was considered not to have prenatal substance exposure. If any of the above items were missing, the subject was excluded from analyses. Additionally, due to the known effect of prenatal caffeine exposure[4,5], we also validated our mean global and network TE results by modifying our covariate for prenatal substance exposure (0 or 1) to include weekly or daily caffeine usage at any time during the pregnancy (excluding subjects missing information of prenatal caffeine exposure).

Substance	FH+	FH-	FH+/-
Tobacco	37 (8.4%)	14 (1.2%)	13 (4.7%)
Alcohol	19 (4.3%)	19 (1.6%)	9 (3.2%)
Marijuana	16 (3.6%)	3 (0.3%)	2 (0.7%)
Morphine	0	0	0
Oxycontin	1 (0.2%)	1 (0.1%)	0
Crack	0	0	0
Amphetamines	1 (0.2%)	0	0
Benzodiazepines	0	0	0
Caffeine	0	1 (0.1%)	0
Cathinones	0	0	0
Synthetic marijuana	0	0	0
GHB	0	0	0
Hallucinogens	0	0	0
Inhalants	0	0	0
Ketamine	0	0	0
MDMA	0	0	0
Opioids	1 (0.2%)	0	0
Barbiturates	0	0	0
Other	1 (0.2%)	0	0

Table S2. Frequency of prenatal substance use among FH+, FH–, and FH+/– groups. Values are n (%).

S3 Family history of substance use disorder by substance type.

The ABCD dataset includes information on whether family members exhibited problems consistent with alcohol use disorders (AUD) or drug use disorders (DUD) but does not provide more detailed subcategories. Here, we present subjects categorized based on family history of AUD and DUD. Our original criteria for family history of SUD classification - FH+ (one or more parents and/or two or more grandparents), FH- (no parents or grandparents), or FH+/- (one grandparent) —were based on family history of any SUD due to evidence of general liability to SUD[6,7]. When considering AUD or DUD specifically, we reclassified subjects using the same criteria for each respective type of SUD. Consequently, some subjects categorized as FH+ in the main text may not meet the criteria for either AUD or DUD individually and instead have a mixed family history involving both disorders, while other subjects meet criteria for both AUD and DUD. Thus, subjects can be categorized into seven groups: FH+ for AUD (1 parent and/or 2+ grandparents with AUD), FH+/- for AUD (1 grandparent with AUD), FH+ for DUD (1 parent and/or 2+ grandparents with DUD), FH+/- for DUD (1 grandparent with DUD), FH+ for mixed SUDs (1 parent and/or 2+ grandparents with any SUD), FH+ for both AUD and DUD (1 parent and/or 2+ grandparents with AUD, and 1 parent and/or 2+ grandparents with DUD), or FH- for both.

Type of SUD	FH+		FH+/–		FH–	
	n	%	n	%	n	%
AUD	194	44.50	244	88.73	—	—
DUD	48	11.01	31	11.27	—	—
Mixed[a]	78	17.89	—	—	—	—
Both[b]	116	26.61	—	—	—	—
None	—	—	—	—	1176	100.00

Table S3. Distribution of subjects by specific family history (FH) categories and type of substance use disorder (SUD). Values are counts (n) and percentages (% of the column total within each FH group). [a] Mixed = family history involving both AUD and DUD but not meeting criteria for each disorder independently. [b] Both = meets criteria for AUD and DUD independently (i.e., satisfies FH criteria for both).

S4 Cluster Number Selection and Validation

S4.1 Choosing *k*

We performed 10 repetitions of k-means clustering for k=2 to k=13. The maximum value (i.e., 13) of k was chosen to satisfy the following condition:

$$k^2 < \text{minimum number of frames across subjects.}$$

We quantified the variance explained by clustering as the ratio of between-cluster variance to total variance in the data[8–10]. We chose k by plotting the variance gained by increasing k, to observe where increasing k begins to provide diminishing returns in terms of variance explained. The elbow occurs around k = 4–5. For $k \geq 4$, the additional variance explained drops below 1%. We therefore focused on k = 4 and replicated results at k = 5, consistent with prior work[8,9].

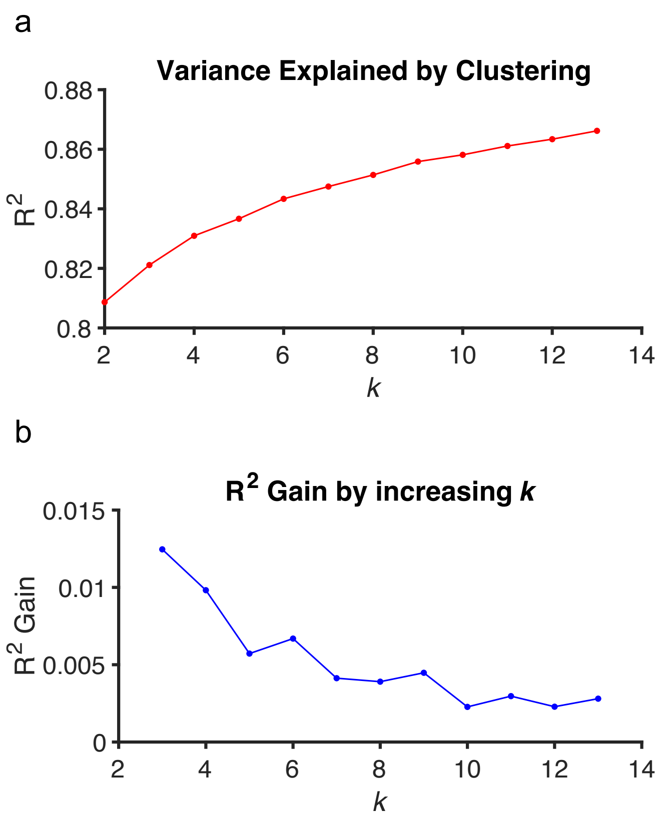

Figure S1. **Choosing *k*.**(a) Elbow Plot of the variance explained by clustering for each choice of *k*, showing an 'elbow' around *k*=4 to *k*=5. (b) Plot of the gained variance explained by increasing *k*. Increasing *k* beyond *k*=4 results in less than a 1% increase in variance explained.

S4.2 Assessing the stability of clustering

As mentioned in the main text, we performed 10 repetitions of k-means clustering, choosing the lowest error solution. To ensure this solution was a consistent global minimum, we repeated this entire process 10 times and compared the adjusted mutual information (AMI) shared between the 10 partitions. The partition that had the maximum sum of AMI scores with all other partitions was selected for analysis. More importantly, this process confirmed that k-means clustering was highly consistent and stable (AMI > 0.99).

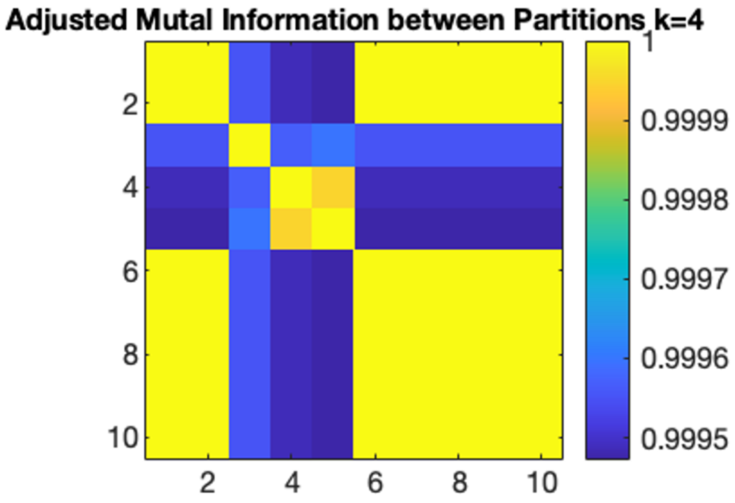

Figure S2. The adjusted mutual information (AMI) shared between 10 independently generated partitions of our data at k=4. Colors reflect adjusted mutual information values, which can range from 0 to 1, with 1 indicating identical partitions. The AMI between all partitions was >0.99.

S5 Clustering results: *k*=4 brain states

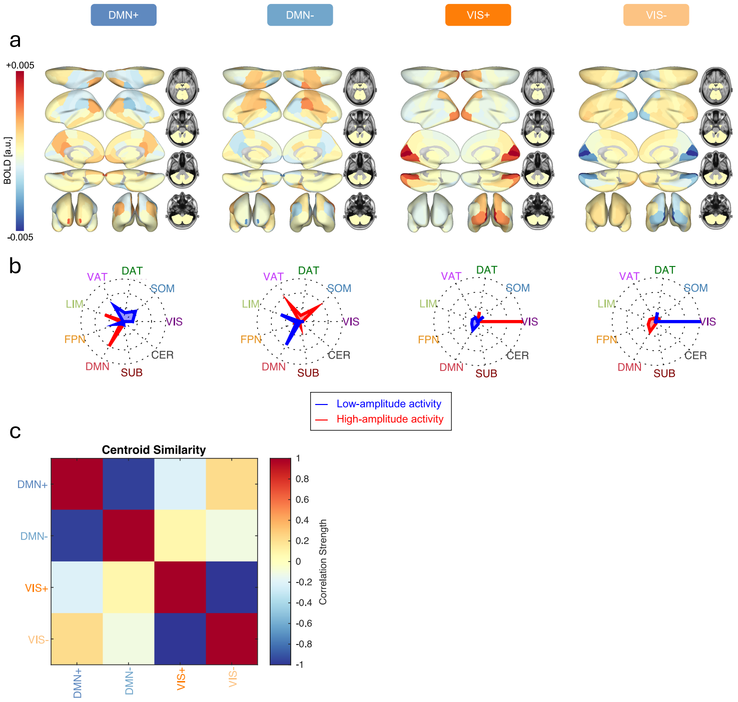

Figure S3. **Four recurrent brain states identified via *k*-means clustering across all subjects (N=1886)**. (a) Group-average centroids of mean BOLD activation for each of *k*=4 brain states plotted on the cortical and subcortical surface (a.u. = arbitrary units). (b) Cosine similarity between each state's centroid and canonical resting-state networks (RSNs)[11], computed separately for positive (high-amplitude) and negative (low-amplitude) components. Each state was assigned the RSN label with the maximal similarity, with a sign indicating whether the match was based on high (+) or low (–) amplitude activity. (c) Pearson correlation between each pair of brain states. Abbreviations: CER = cerebellum, DAT = dorsal attention network, DMN = default mode network, FPN = frontoparietal network, LIM = limbic network, SOM = somatomotor network, SUB = subcortex, VAT = ventral attention network, VIS = visual network.

S6 Global transition energy: ANCOVA results

Variable	F(df)	p	p_{FDR}	η_p^2
**Main effects**				
Sex	25.93 (1, 1585)	$\mathbf{3.96{\times}10^{-7}}$	$\mathbf{2.77{\times}10^{-6}}$	0.016
Age	0.46 (1, 1585)	0.499	0.699	0.000
Family History of SUD (FH+ vs FH-)	0.05 (1, 1585)	0.824	0.900	0.000
Puberty	2.01 (2, 1585)	0.134	0.269	0.003
Framewise Displacement	0.62 (1, 1585)	0.430	0.668	0.000
MRI Scanner Model	24.98 (2, 1585)	$\mathbf{2.08{\times}10^{-11}}$	$\mathbf{2.91{\times}10^{-10}}$	0.031
Household Income	0.33 (2, 1585)	0.720	0.900	0.000
Parental Education	1.60 (4, 1585)	0.171	0.299	0.004
Race/Ethnicity	0.14 (4, 1585)	0.968	0.968	0.000
Prenatal Substance Exposure	0.04 (1, 1585)	0.836	0.900	0.000
Parental History of Mental Health	6.05 (1, 1585)	**0.014**	0.065	0.004
**Interactions**				
Sex × Family History of SUD	4.00 (1, 1585)	**0.046**	0.128	0.003
Family History of SUD × Household Income	2.05 (2, 1585)	0.129	0.269	0.003
Sex × Puberty	3.16 (2, 1585)	**0.043**	0.128	0.004

Table S4. ANCOVA results for mean global transition energy ($N = 1611$; FH+ and FH- subjects only). Reported are F-statistics with numerator and denominator degrees of freedom in parentheses, p-values, Benjamini–Hochberg corrected p-values (corrected across all variables), and partial eta-squared (η_p^2) effect sizes. Significant results at $p < 0.05$ (uncorrected or FDR-corrected) are in bold.

S7 Pairwise regional transition energy: post hoc *t*-tests.

S7.1 Post-hoc *t*-tests of pairwise regional transition energy in regions found to have significant effect of family history of SUD (across both males and females).

For each pair of brain states (*k*x*k*), we performed post hoc *t*-tests for the pairwise regional TE in each of the seven regions found to be significant for family history of SUD (FH+ vs FH-).

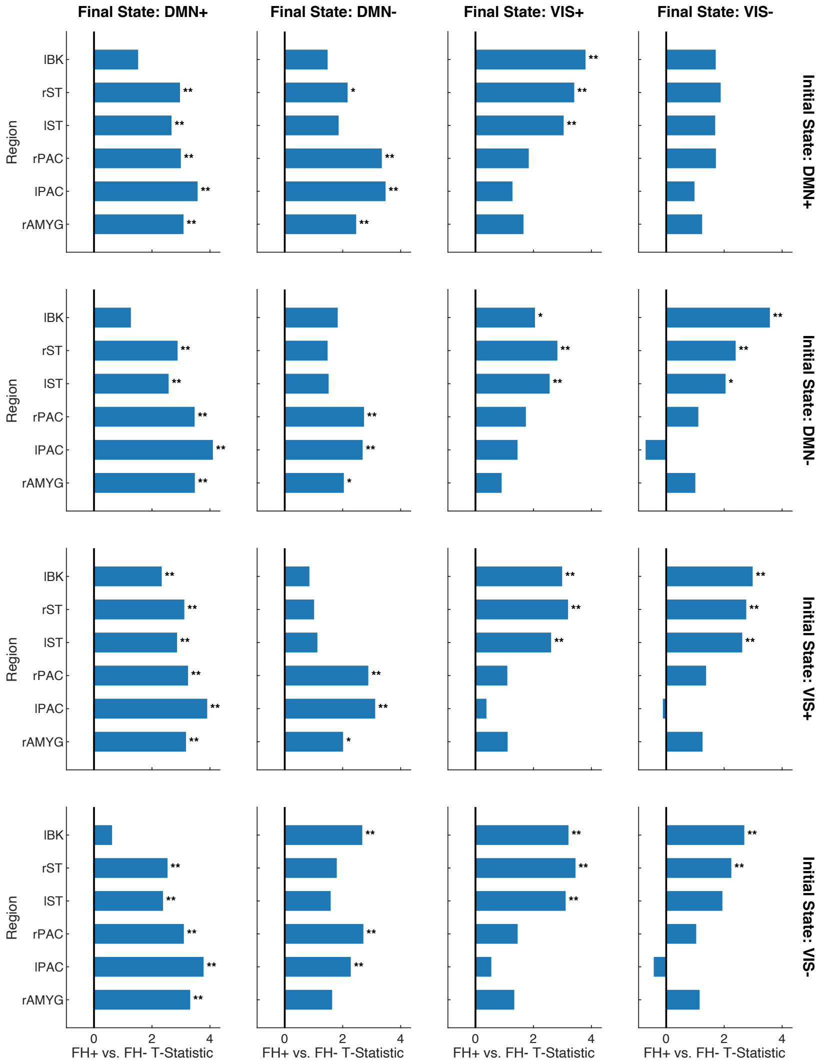

Figure S4. Pairwise regional transition energies: FH+ versus FH-. Bar plots of *t*-statistics from *t*-tests (unpaired, two-sided) comparing of pairwise regional TE values of FH+ and FH- subjects. Regions are limited to only those shown to have a significant main effect of family history of SUD in ANCOVA models (see main text). *P*-values were corrected for multiple comparisons using the Benjamini-Hochberg procedure. * = uncorrected $p < 0.05$; ** = $p_{FDR} < 0.05$. N = 1611 (436 FH+, 1175 FH-).

S7.2 Post-hoc *t*-tests of pairwise regional transition energy in regions found to have significant effect of the interaction between sex and family history of SUD.

For each pair of brain states, we performed post hoc *t*-tests on the pairwise regional TE in each of the eight regions found to be significant for the interaction of sex and family history of SUD (within-sex FH+ vs FH-).

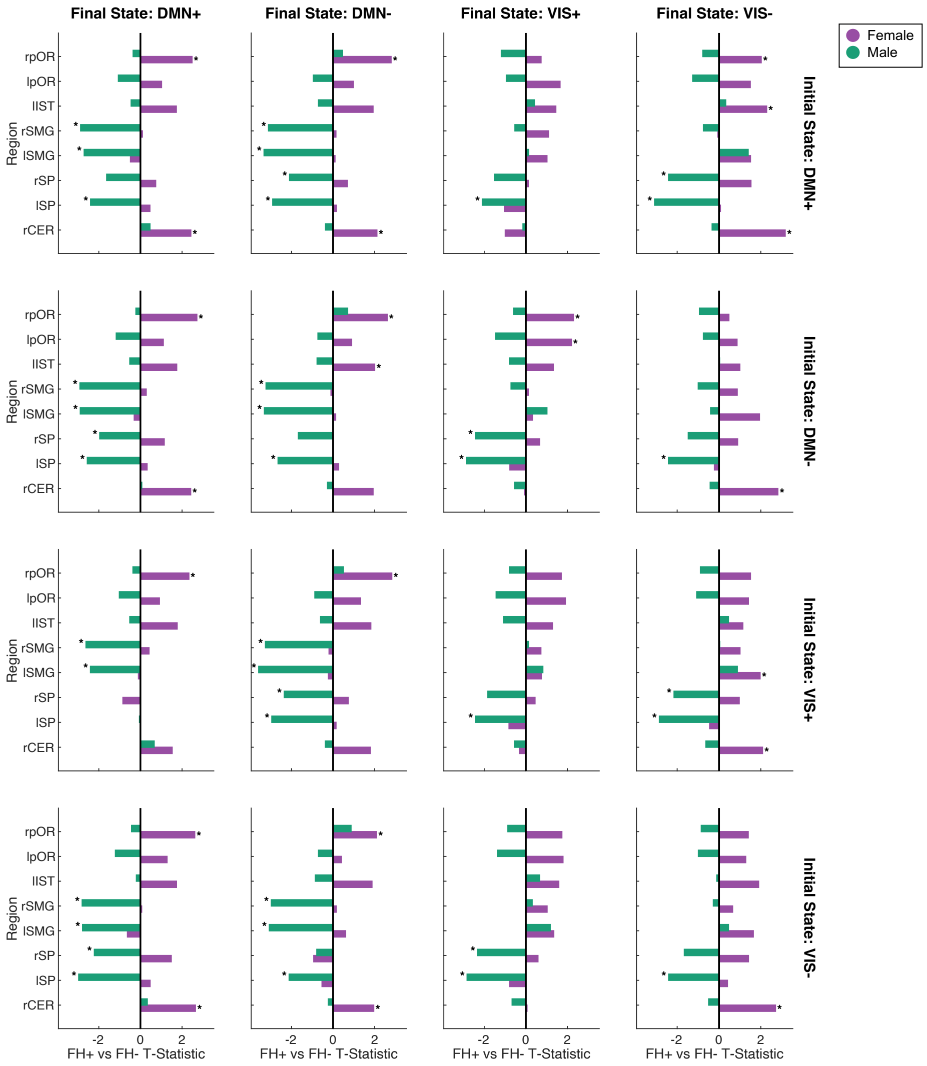

Figure S5. Pairwise regional transition energies: FH+ versus FH- in females and males. Bar plots of *t*-statistics from within-sex *t*-tests (unpaired, two-sided) of pairwise regional TE values. Regions shown were those with a significant family history-by-sex interaction effect in ANCOVA models (see main text). Green bars indicate results for males (FH+ vs. FH-) and purple bars indicate results for females (FH+ vs. FH-). *P*-values were corrected for multiple comparisons using the Benjamini–Hochberg procedure. * = uncorrected $p < 0.05$; ** = $p_{FDR} < 0.05$. N = 1611 (238 FH+ females, 626 FH- females, 198 FH+ males, 549 FH- males).

S8 Full statistical results for ANCOVA models.

S8.1 Mean network TE: ANCOVA F-statistics and *p*-values.

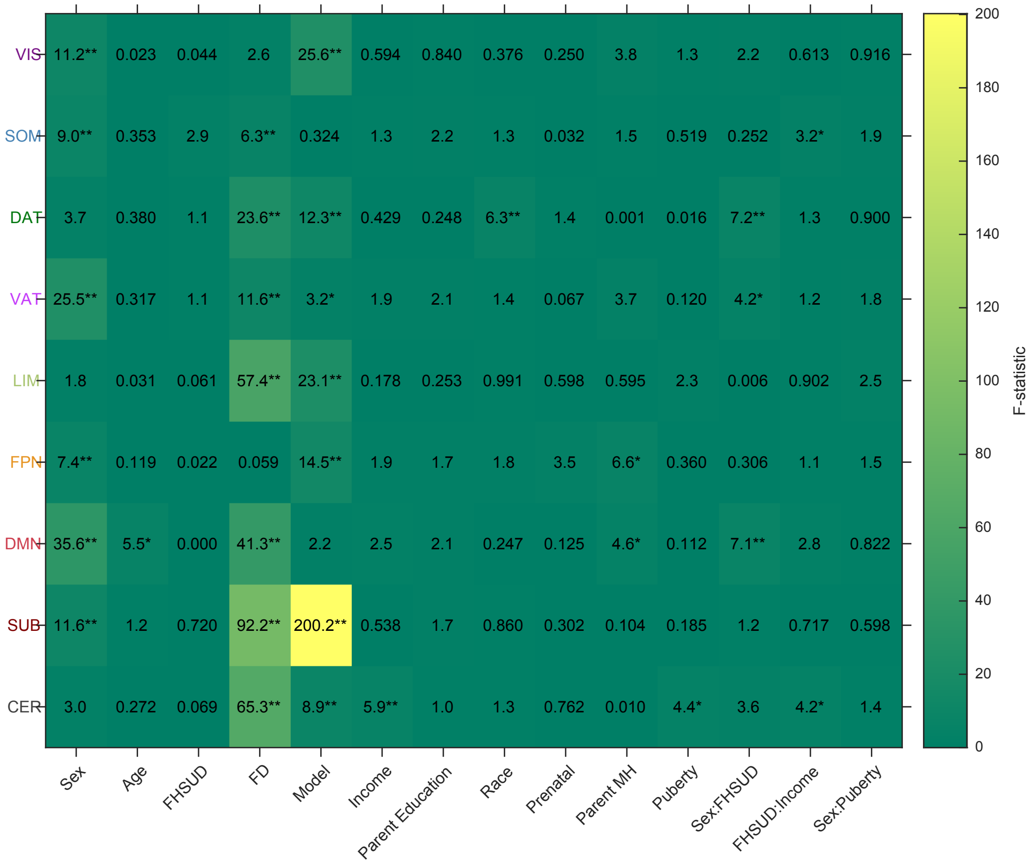

Figure S6. F-statistics for all variables included in ANCOVA models on mean network TE (see main text). *P*-values were corrected for multiple comparisons using the Benjamini–Hochberg procedure. * = uncorrected $p < 0.05$; ** = $p_{\text{FDR}} < 0.05$. N = 1611 (238 FH+ females, 626 FH- females, 198 FH+ males, 549 FH- males).

S8.2 Mean network TE: ANCOVA partial eta-squared and *p*-values.

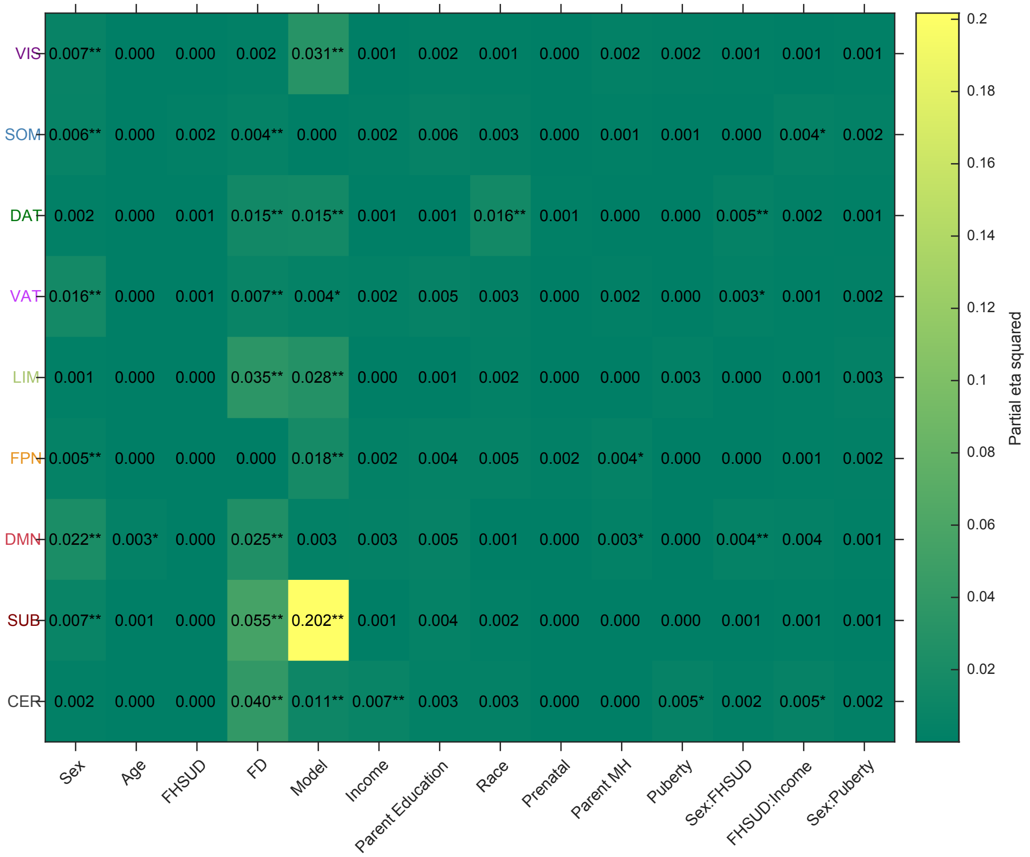

Figure S7. Partial eta-squared (η_p^2) values for effect size of all variables included in ANCOVA models on mean network TE (see main text). *P*-values were corrected for multiple comparisons using the Benjamini–Hochberg procedure. * = uncorrected $p < 0.05$; ** = $p_{\mathrm{FDR}} < 0.05$. N = 1611 (238 FH+ females, 626 FH- females, 198 FH+ males, 549 FH- males).

S8.3 Mean regional TE: ANCOVA F-statistics and *p*-values.

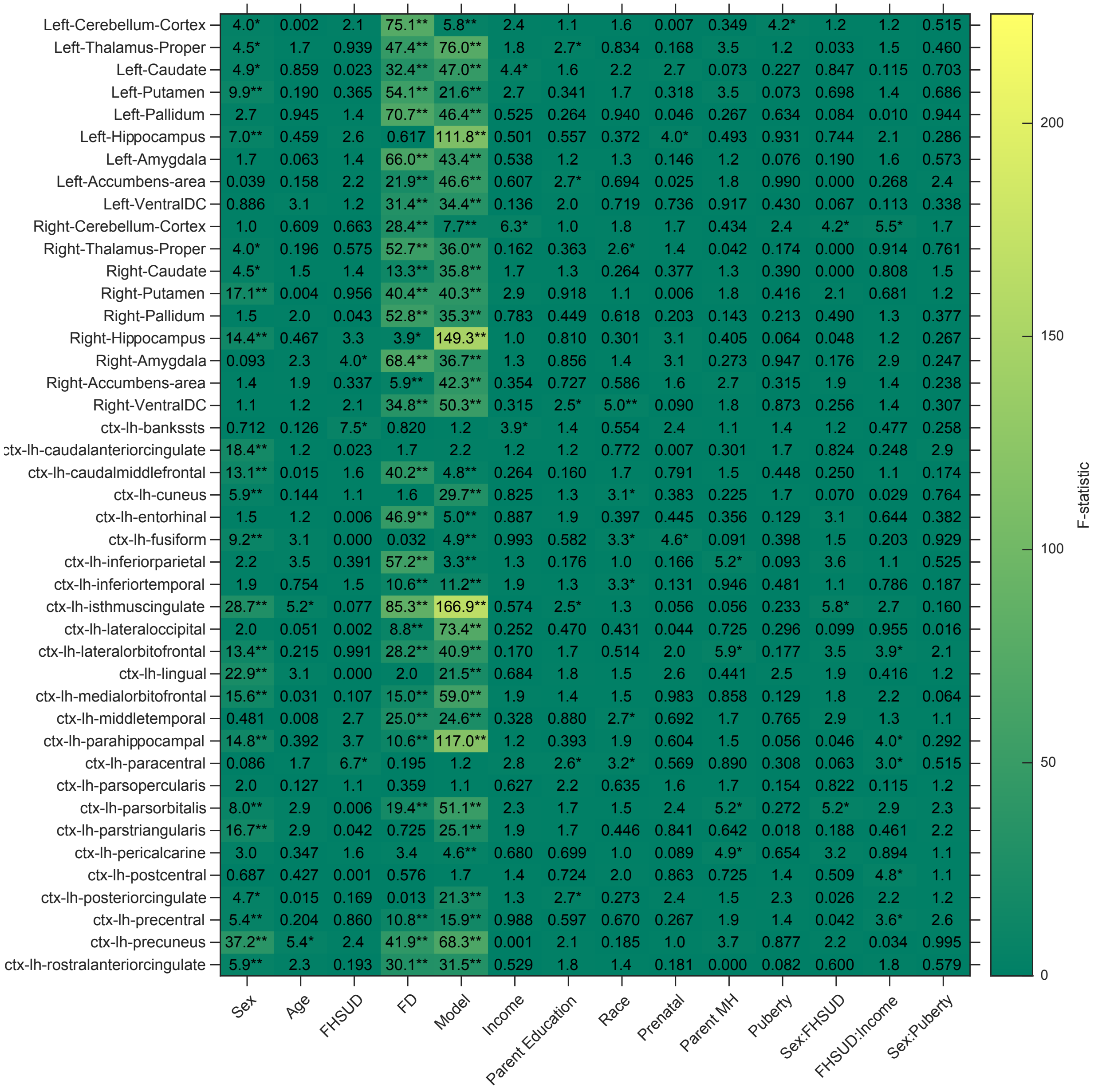

Figure S8. F-statistics for all variables included in ANCOVA models on mean regional TE values (see main text). Regions 1 to 43. *P*-values were corrected for multiple comparisons using the Benjamini–Hochberg procedure. * = uncorrected $p < 0.05$; ** = $p_{FDR} < 0.05$. N = 1611 (238 FH+ females, 626 FH- females, 198 FH+ males, 549 FH- males).

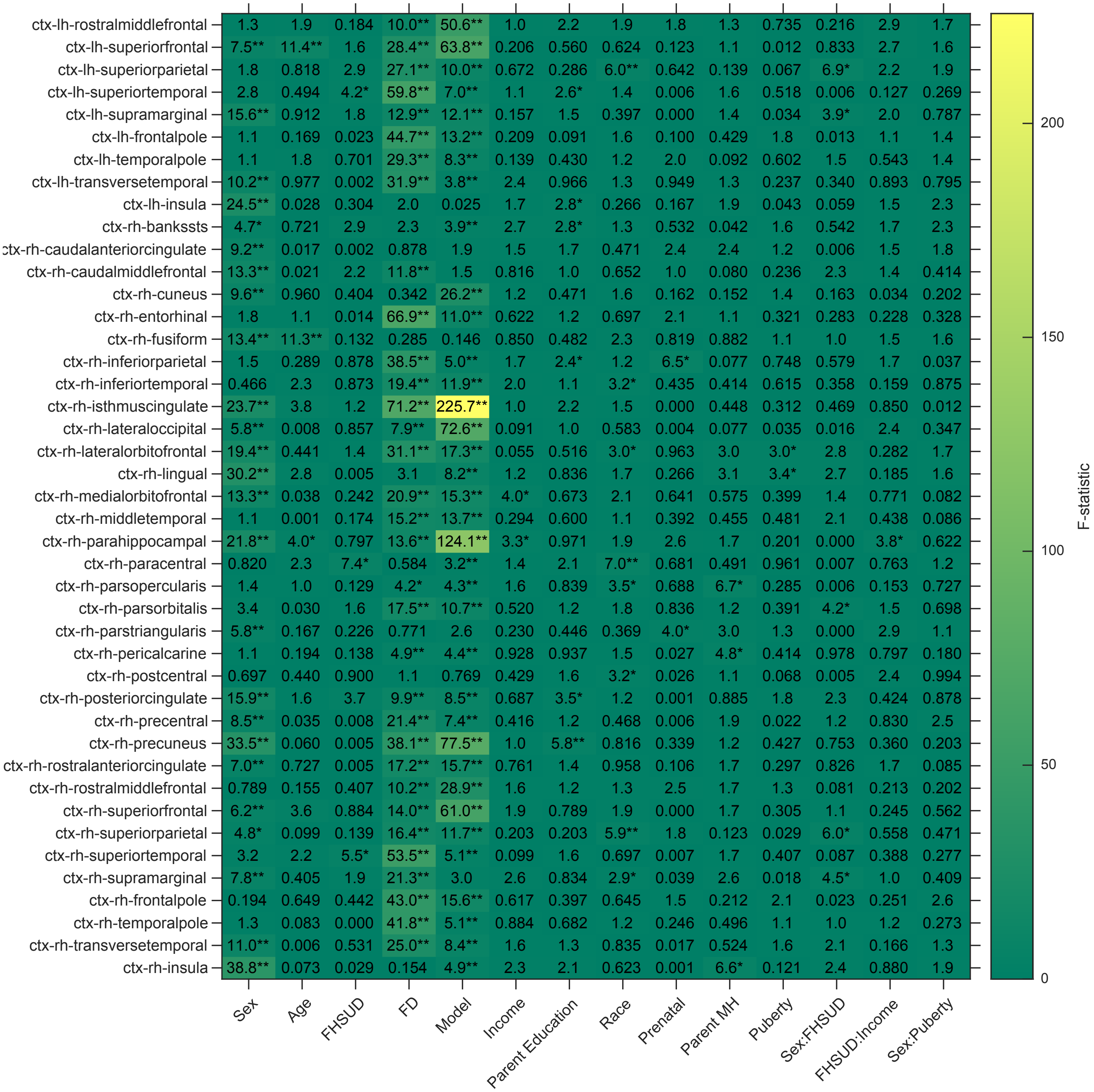

Figure S9. F-statistics for all variables included in ANCOVA models on mean regional TE values (see main text): regions 44 to 86. *P*-values were corrected for multiple comparisons using the Benjamini–Hochberg procedure. * = uncorrected $p < 0.05$; ** = $p_{FDR} < 0.05$. N = 1611 (238 FH+ females, 626 FH- females, 198 FH+ males, 549 FH- males).

S8.4 Mean regional TE: ANCOVA partial eta-squared and *p*-values.

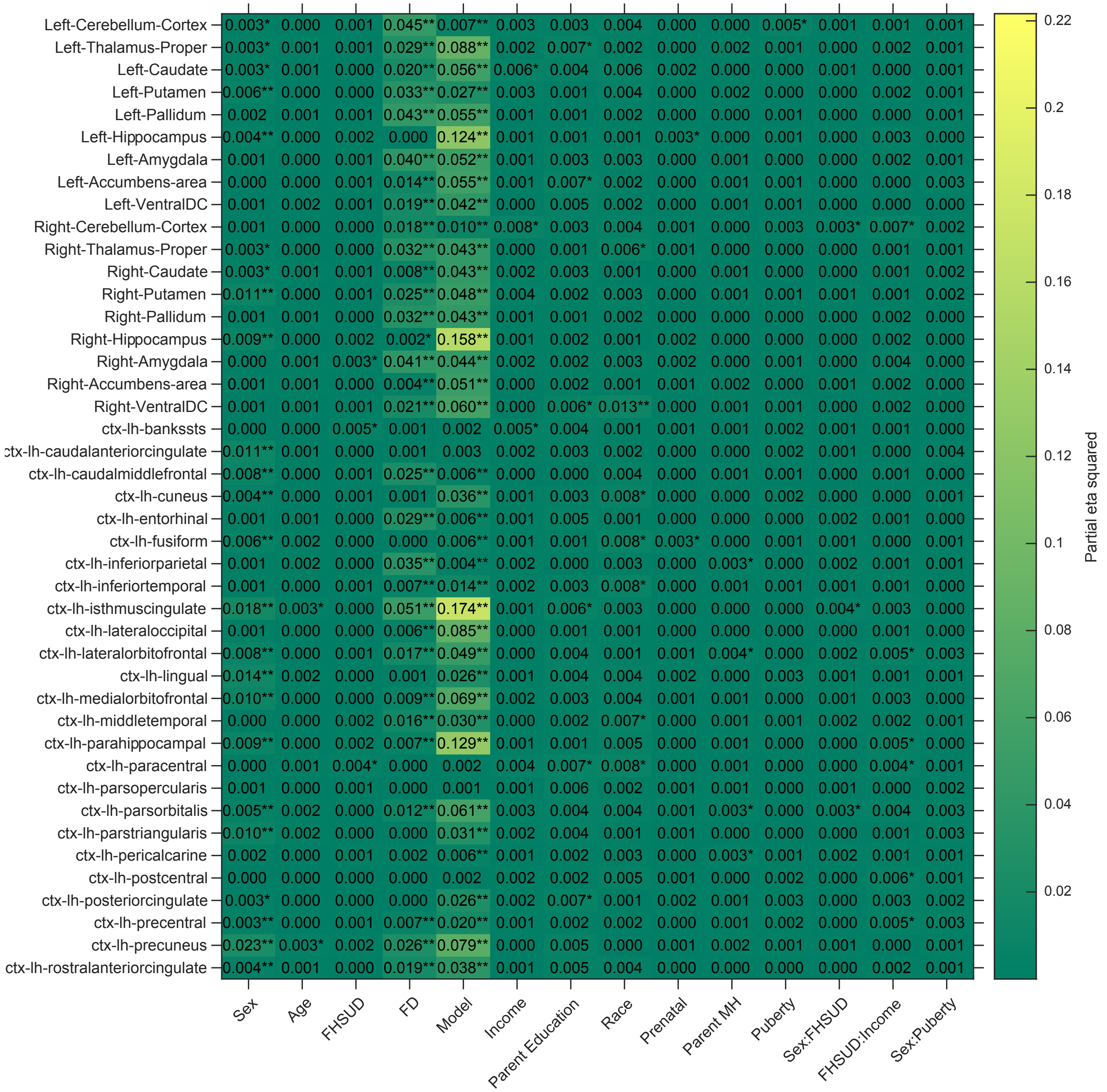

Figure S10. Partial eta-squared (η_p^2) values for effect size of all variables included in ANCOVA models on mean regional TE values (see main text). Regions 1 to 43.*P*-values were corrected for multiple comparisons using the Benjamini–Hochberg procedure. * = uncorrected $p < 0.05$; ** = $p_{\mathrm{FDR}} < 0.05$. N = 1611 (238 FH+ females, 626 FH- females, 198 FH+ males, 549 FH- males).

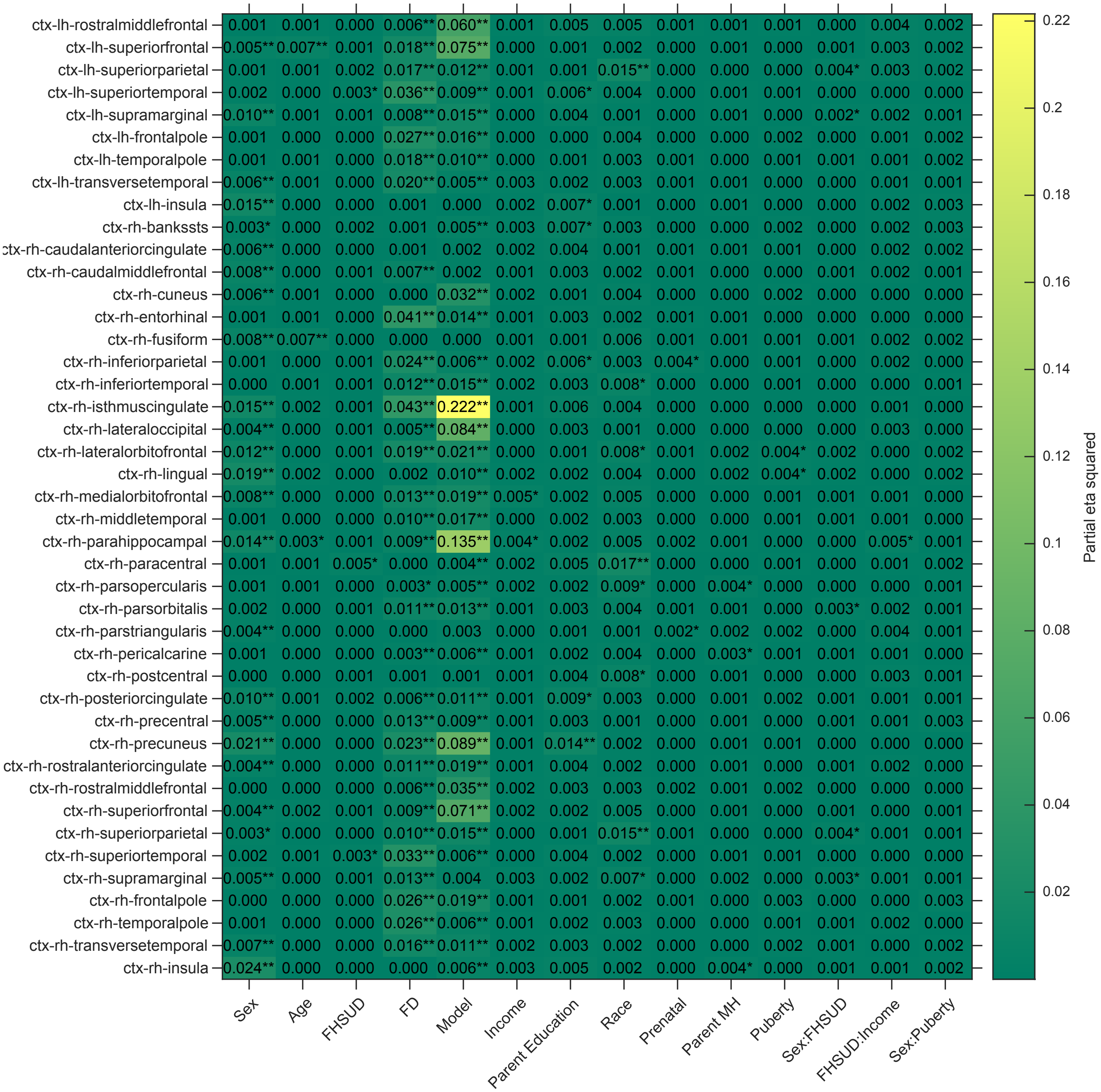

Figure S11. Partial eta-squared (η_p^2) values for effect size of all variables included in ANCOVA models on mean regional TE values. Regions 44 to 86. *P*-values were corrected for multiple comparisons using the Benjamini–Hochberg procedure. * = uncorrected $p < 0.05$; ** = $p_{\mathrm{FDR}} < 0.05$. N = 1611 (238 FH+ females, 626 FH- females, 198 FH+ males, 549 FH- males).

S9 Fractional occupancy of brain states.

For all subjects, we calculated the fractional occupancy (FO) (i.e., the percentage of frames assigned to a state for a given subject) of each k=4 brain state, following previous analyses[8]. We ran ANCOVA models on the FO of each brain state (including same covariates: sex, age, parental education, household income, race, parental history of mental illness, prenatal substance exposure, pubertal stage, family history of SUD, framewise displacement, MRI model, the interaction effects of sex and family history of SUD, household income and family history of SUD, and sex and puberty) and found no significant effect of family history of SUD or the interaction of sex and family history of SUD on FO of any brain state.

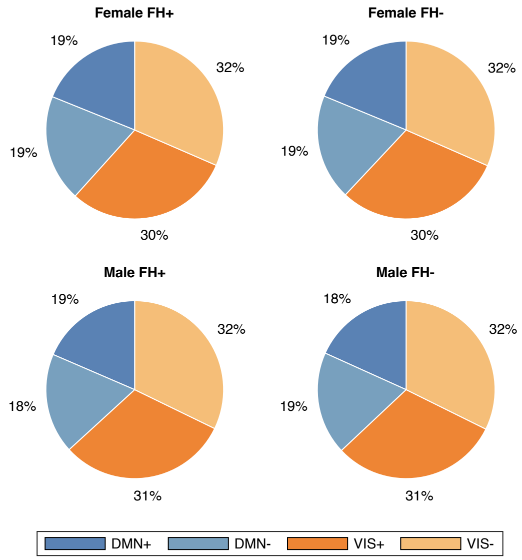

Figure S12. The group average of the fractional occupancies of k=4 brain states is plotted as a pie chart for each of the four family history-by-sex groups. Color represents the four brain states: DMN+ (dark blue), DMN- (light blue), VIS+ (green), VIS- (yellow). N = 1611 (238 FH+ females, 626 FH- females, 198 FH+ males, 549 FH- males).

	DMN+	DMN-	VIS+	VIS-
**Family history of SUD**	$F(1,1585) = 0.41$<br>$p = 0.52$<br>$\eta_p^2 = 0.0003$	$F(1,1585) = 0.29$<br>$p = 0.59$<br>$\eta_p^2 = 0.0018$	$F(1,1585) = 0.0009$<br>$p = 0.98$<br>$\eta_p^2 = 5.9 \times 10^{-7}$	$F(1,1585) = 0.01$<br>$p = 0.92$<br>$\eta_p^2 = 6.4 \times 10^{-6}$
**Sex × Family history of SUD**	$F(1,1585) = 1.31$<br>$p = 0.25$<br>$\eta_p^2 = 0.0008$	$F(1,1585) = 0.12$<br>$p = 0.73$<br>$\eta_p^2 = 7.5 \times 10^{-5}$	$F(1,1585) = 1.30$<br>$p = 0.25$<br>$\eta_p^2 = 0.0008$	$F(1,1585) = 0.026$<br>$p = 0.87$<br>$\eta_p^2 = 1.7 \times 10^{-5}$

Table S5. ANCOVA results for fractional occupancy in FH+ and FH– subjects across four brain-state clusters. Each cell reports $F(df_{\text{effect}}, df_{\text{error}})$, uncorrected p-value, and partial eta squared (η_p^2).

S10 Robustness analyses: replications

S10.1 Replication of main results with *k*=5.

We replicated our main analysis with *k*=5 to assess the robustness of our findings. The analysis was performed in the same cohort (FH+ and FH- subjects meeting inclusion criteria without outlier global TE values; N = 1611: 238 FH+ females, 626 FH- females, 198 FH+ males, 549 FH- males). At *k*=5, we identified the same four brain states observed with *k*=4, plus an additional state characterized by positive amplitude activity in the limbic network (LIM+). All major results and trends were preserved; however, the family history-by-sex effect on mean DMN TE did not reach significance.

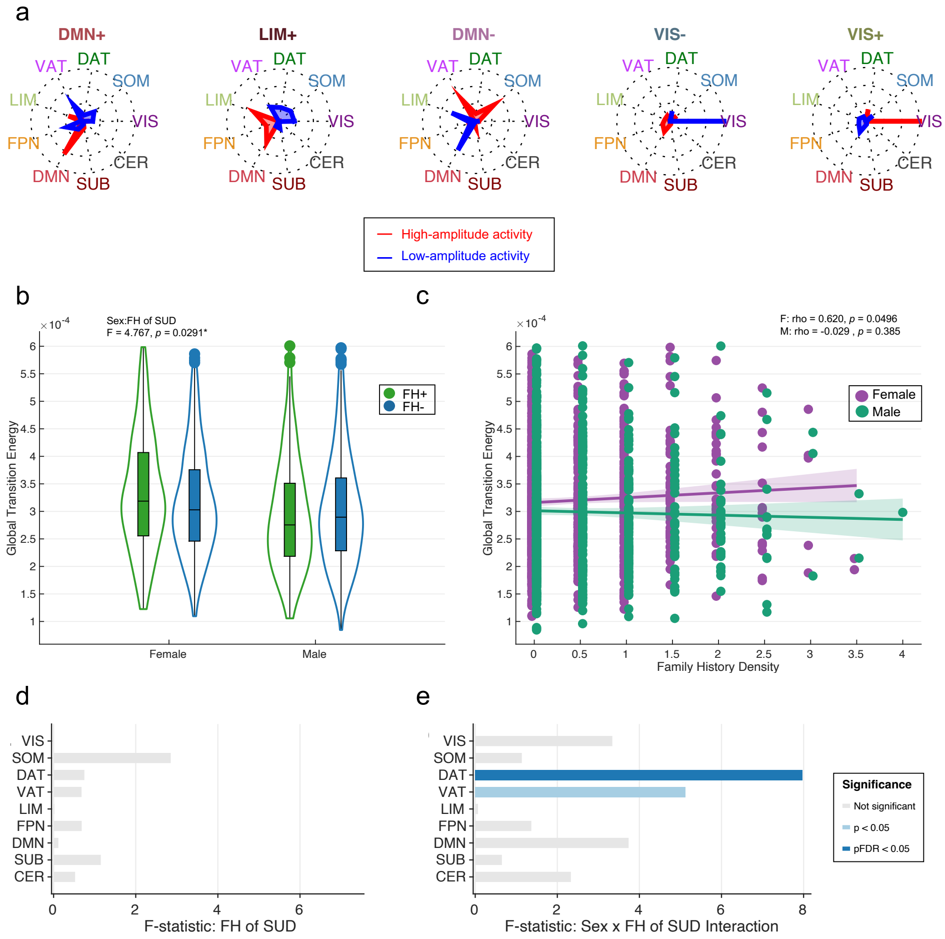

Figure S13. Replication of the main analysis with k=5. (a) Radar plots of group-average centroids for the k=5 clusters. Red lines indicate high-amplitude activity and blue lines indicate low-amplitude activity. (b) Violin plots of mean global TE. The ANCOVA model for global TE revealed a significant interaction between sex and family history of SUD after correction. Data distributions are shown with violins; box plots display the median (center line), interquartile range (25th-75th percentiles; box bounds), and whiskers extending to the most extreme values within 1.5×IQR; points beyond the whiskers are plotted as outliers. Green denotes FH+ and blue denotes FH- subjects, separated by sex. (c) Spearman correlations between family history density and mean global TE in males and females revealed a positive association in females (uncorrected). Solid lines represent generalized linear model fits, with shaded bands indicating 95% confidence intervals. Each point represents one subject; purple denotes females and green denotes males. (d–e) ANCOVA F-statistics for (d) the main effect of family history of SUD and (e) the interaction between sex and family history of SUD on mean network TE across the nine networks. For (b-c), * = uncorrected $p < 0.05$; ** = $p_{FDR} < 0.05$. For (d-e), grey bars indicate non-significant results, light blue indicates $p < 0.05$, and dark blue indicates $p_{FDR} < 0.05$. Sample size: N = 1611 (238 FH+ females, 626 FH- females, 198 FH+ males, 549 FH- males).

S10.2 Replication in adolescents from the NCANDA dataset.

We replicated our results in an external cohort of individuals from the National Consortium on Alcohol and Neurodevelopment in Adolescence (NCANDA) dataset (Data Release: NCANDA_PUBLIC_BASE_RESTINGSTATE _V01)[12]. Adult participants consented to participating, and minors provided written assent along with consent from a parent/legal guardian. Participants were compensated for completing all baseline sessions, and parents were compensated for completing the baseline interview, with total compensation ranging from $200 to $225 per family across sites. Participants discovered to be ineligible received partial compensation based on the measures completed before exclusion.

Baseline resting-state fMRI data were pre-processed using the publicly-available NCANDA pipeline[13], which consisted of motion correction, outlier-detection, detrending, physiological noise removal as well as temporal (low pass frequency: 0.1, high pass frequency: 0.01) and spatial smoothing. Frames in individual rsfMRI time series were labeled as outliers if framewise displacement > 0.3 mm/TR. After removing scans with usable frames < 7.8 min, each of the rsfMRI images of the remaining 715 subjects (aged 12-21 years old, 52% female) were registered to the SRI24 atlas[14] and parcellated into 90 regions (80 cortical and 10 subcortical). The BOLD time series was normalized by the mean gray matter BOLD signal (pre-spatial and temporal filtering). We subsequently removed all censored frames, plus one frame before and two frames after, as well as uncensored segments of fewer than five contiguous frames. Using probabilistic tractography, individual structural connectomes (SC) were computed and parcellated into the same 90 regions.

Of these 715 subjects, we excluded those exceeding alcohol, tobacco, marijuana, or other drug usage thresholds (N = 132)[12], as well as subjects aged $\geq$16 years (N = 260) to better match our ABCD cohort and minimize substance exposure. Following the same NCT analysis as in the main text, we next applied k-means clustering to BOLD time series data of these 260 subjects and computed mean global, pairwise global, and regional TEs using a group-average SC. Outlier subjects (N=76) were excluded if their mean global TE exceeded $\pm$3 scaled median absolute deviations. Due to the smaller cohort size and greater age-range compared to our ABCD cohort, we sex-, age-, and in-scanner motion–matched the cohort. We then performed 500 iterations of a matching algorithm to pair each FH+ subject with a same-sex FH– subject, selecting the solution with the smallest average difference in age (0.03 years) and mean framewise displacement (0.03 mm). Our final sample included N = 64 subjects (FH+ females N = 20, FH- females N= 20, FH+ males N = 12, FH- males N = 12).

We next ran an ANCOVA on mean global TE including sex, age, sex:age, family history of SUD, race/ethnicity, in-scanner motion, socioeconomic status (SES, i.e., parent education recoded as in ABCD), MRI scanner model (GE Discovery MR750 vs Siemens TrioTim), sex:family history of SUD, and family history of SUD:SES. As in our main results, we find FH+ females > FH- females, and FH+ males < FH- males in mean global TE (Figure S14. While family history of SUD had a very small effect size (F= 0.28, p = 0.59, η_p^2 = 0.006), the interaction of sex and family history of SUD trended towards significance (F = 2.82, p = 0.099, η_p^2 = 0.059). Spearman rank correlations found family history density was weakly positively and negatively correlated with mean global TE in females (rho = 0.11, p = 0.52) and males (rho = -0.22, p = 0.31), respectively, though the correlations were not significant.

The effect of family history of SUD was not significant for the mean regional TE of any regions. Mean regional TE of seven regions were found to have significant (before correction only) family history-by-sex effects: the bilateral cuneus, bilateral middle occipital gyrus, left paracentral lobule, the right precuneus, and the right pars orbitalis of the inferior frontal orbitalis. For all these regions, FH+ > FH- females and FH+ < FH- males. Notably, family history-by-sex effects were identified in cortical regions only, replicating our observations in the ABCD cohort. Additionally, we replicated our result of a significant family history-by-sex effect on the mean regional TE of the right pars orbitalis ("Frontal_Inf_Orb_R"), which exhibited the greatest difference in mean regional TE of all regions in FH+ > FH- females via unpaired t-tests (t = 2.195, p = 0.0343, pFDR = 0.426, Cohen's d = 0.694). On the other hand, whereas we found a significant effect of family history of SUD on mean regional TE of the bilateral paracentral lobule (FH+ > FH- in both males and females) in the ABCD sample, in this sample we found the left paracentral lobule had a significant family history-by-sex effect driven by lower mean regional TE in males (t = -1.976, p = 0.061, pFDR = 0.426, Cohen's d = -0.806). Overall, most regions showed higher TE in FH+ females and lower TE in FH+ males relative to FH- counterparts (Figure S15).

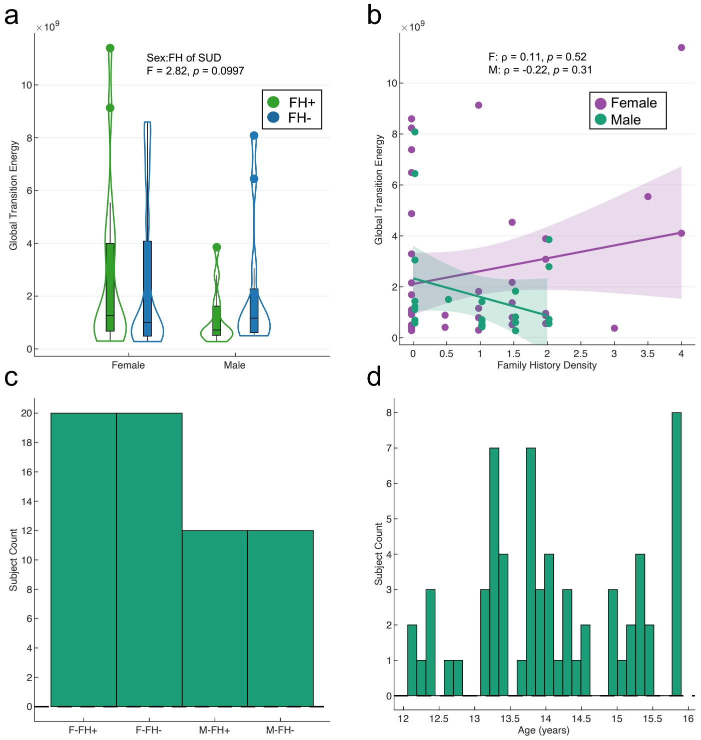

Figure S14. Replication of the FH-by-sex effect on global TE in NCANDA dataset. (a) Mean global TE violin plots. Violin plots show the full data distribution; box plots display the median (line), interquartile range (25th–75th percentiles), whiskers (1.5×IQR), and individual outliers. (b) Spearman correlation revealed replication at the trend level of relationship between of family history density and mean global TE in males (green) and females (purple). Lines reflect generalized linear model fits, with shaded 95% confidence intervals. Each point = one subject. (c) Histogram of number of subjects by sex and family history groups. (d) Histogram of ages of subjects. Total N = 64 subjects; FH+ females N = 20, FH- females N= 20, FH+ males N = 12, FH- males N = 12. * = uncorrected p <0.05 and ** = pFDR < 0.05.

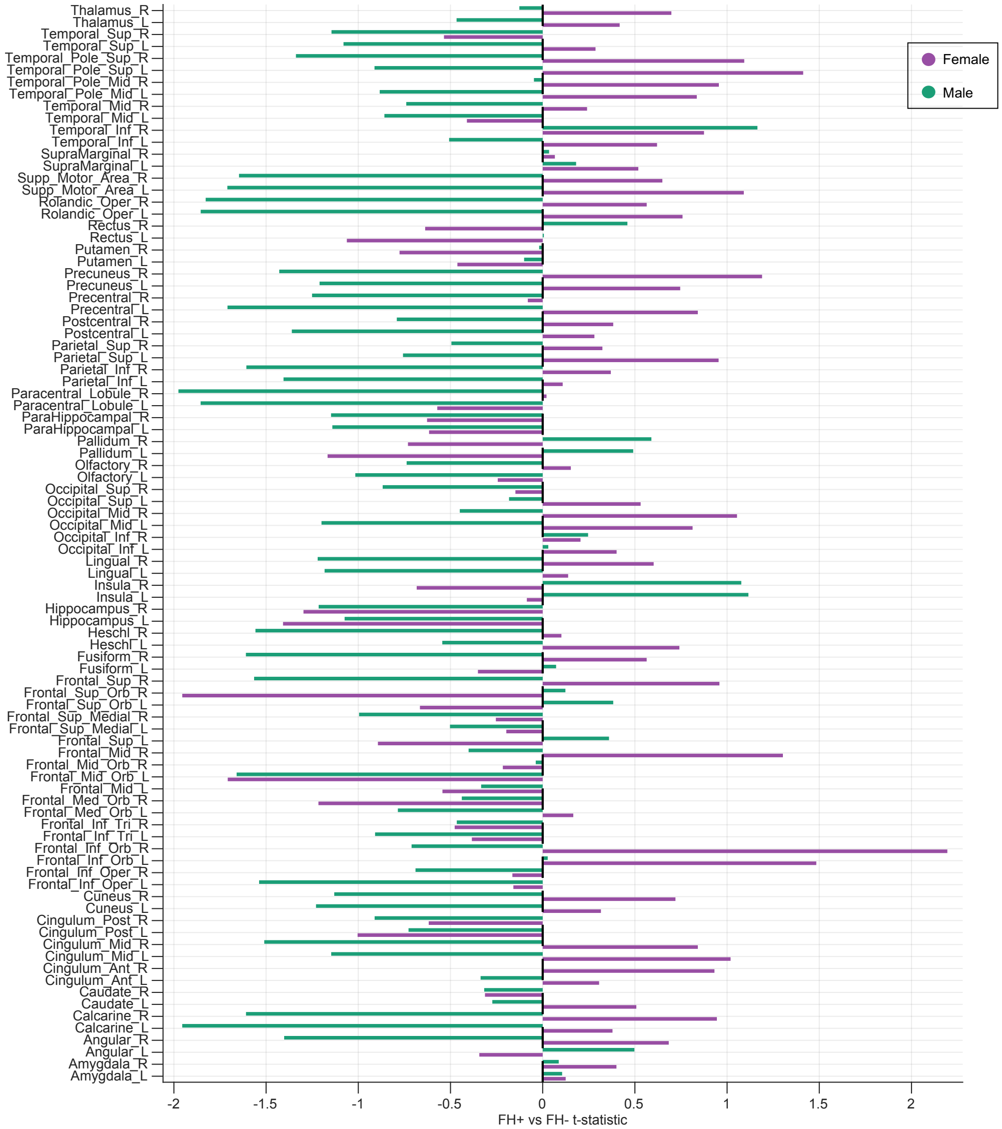

Figure S15. Replication of effect of interaction between sex and family history of SUD on mean regional transition energies in the NCANDA dataset. Bar plot of *t*-statistics from unpaired *t*-tests (two-sided) comparing FH+ and FH- mean regional TE across 90 regions in NCANDA dataset within males (green) and females (purple).

S10.3 Replication with individual structural connectomes in a cortex-only parcellation.

We replicated our main results using individual structural connectomes (SC) instead of a group-average (as in main text) in order to determine whether our results also reflect individual differences in structural connectivity or are driven by functional dynamics alone. Individual structural connectomes (SC) were available to us only in the Desikan-Killiany atlas (68 cortical regions; DK68). Thus for network-based analyses, we could only consider cortical networks (i.e., the Yeo-7 networks[11]) and not subcortical or cerebellar networks. Individual SCs were available for a subset (N = 2080) of our original cohort of ABCD subjects for whom we had usable resting-state functional MRI (rsfMRI) data (N = 2362); see main text for details. After applying the same exclusion criteria, we ran NCT analyses on N = 1709 subjects as described in the main text. We then excluded subjects (N=46) with outlier values of mean global TE. The results of this analysis with individual SCs largely replicate the results reported in the main text using a group-average SC: DAT and DMN both had significant family history-by-sex effects after correction. However, the VAT was not significant and the VIS network was significant (before correction) - a trend visible in our main analyses using a group-average SC. Mean global TE values from a group-average SC and individual SC for N = 1660 overlapping subjects were found to be highly correlated (Pearson rho = 0.998, $p < 0.0001$). Therefore, the minor differences in the results of analyses using individual versus group-average SCs are most likely due to a smaller sample size and thus reduced power rather than the effect of group differences in structural connectivity.

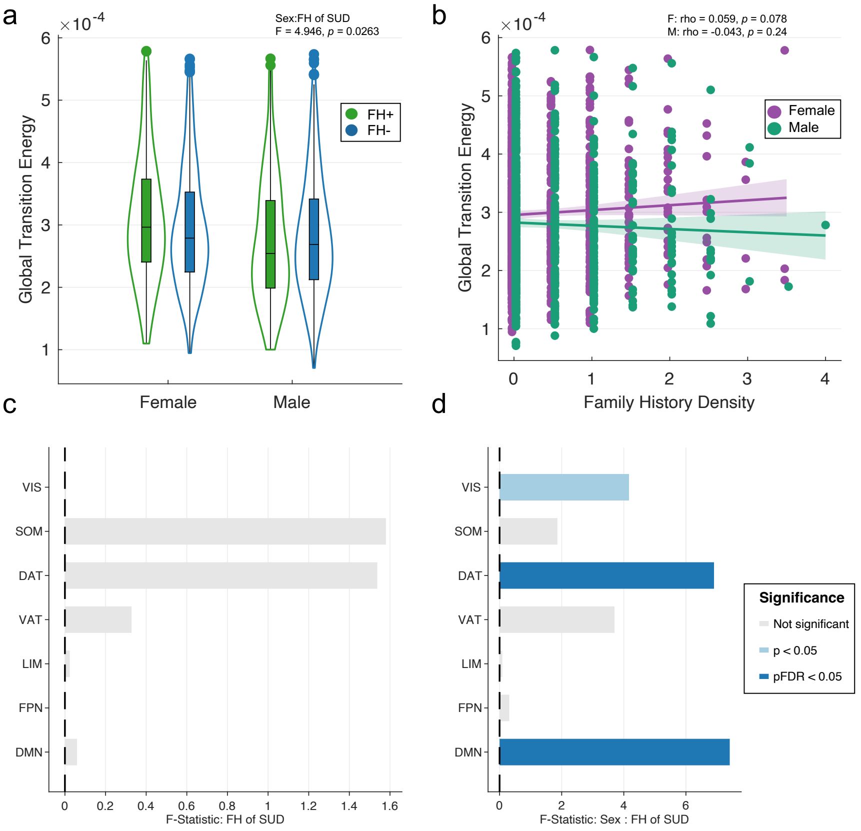

Figure S16. Replication of the main analysis with individual structural connectomes. (a) Violin plots of mean global TE. The ANCOVA model for global TE revealed a significant interaction between sex and family history of SUD after correction. Data distributions are shown with violins; box plots display the median (center line), interquartile range (25th-75th percentiles; box bounds), and whiskers extending to the most extreme values within 1.5×IQR; points beyond the whiskers are plotted as outliers. Green denotes FH+ and blue denotes FH- subjects, separated by sex. (b) Spearman correlations between family history density and mean global TE in males (green) and females (purple) revealed a positive association in females (uncorrected). Solid lines represent generalized linear model fits, with shaded bands indicating 95% confidence intervals. Each point represents one subject. (c–d) ANCOVA F-statistics for (c) the main effect of family history of SUD and (d) the interaction between sex and family history of SUD on mean network TE across the Yeo 7-networks. Grey bars indicate non-significant results, light blue indicates $p < 0.05$, and dark blue indicates $p_{\mathrm{FDR}} < 0.05$. Sample size: N=1709 subjects; FH+ females = 212, FH- females = 560, FH+ males = 162, FH- males = 487.

S11 Robustness analyses: stratified by single site, MRI model, and income level.

S11.1 Analysis within a single-site.

To demonstrate our findings are not the result of collection site effects, we analyzed subjects from within a single site. Site 16 was chosen because it had the largest sample size (N = 307) and previous work has noted site 16 as having "high-quality data" and was chosen as a reference site in a site-harmonization study[15]. In our cohort, all subjects from site 16 utilized the same Siemens Prisma MRI scanner. Using TE calculations from the analysis in the main text of k-means clustering ($k = 4$) from the entire cohort, we ran ANCOVAs on global and network TE values from subjects from site 16 only. We used the same covariates as in the main text, except we did not include MRI model as a covariate as it was Prisma for all subjects. Despite a relatively low N for FH+ subjects of either sex (FH+ females = 18 and FH+ males = 23), we replicated the trends in mean global TE such there was a significant effect of the interaction of sex and family history and FH+ > FH- in females and FH+ < FH- in males. We found mean network TE family history-by-sex effects in the DMN post-correction, and in the DAT and CER pre-correction.

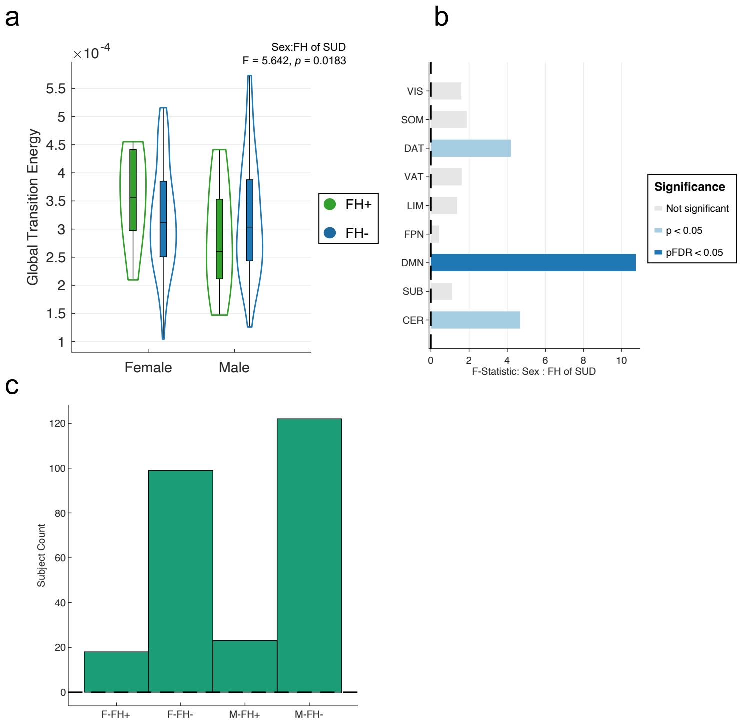

Figure S17. Mean global and mean network TE results from subjects within site 16. (a) Violin plots of mean global TE. The ANCOVA model for global TE revealed a significant (before correction) interaction between sex and family history of SUD. Data distributions are shown with violins; box plots display the median (center line), interquartile range (25th-75th percentiles; box bounds), and whiskers extending to the most extreme values within 1.5×IQR; points beyond the whiskers are plotted as outliers. Green denotes FH+ and blue denotes FH- subjects, separated by sex. (b) ANCOVA F-statistics for the interaction effect between sex and family history of SUD on mean network TE across the nine networks. Grey bars indicate non-significant results, light blue indicates $p < 0.05$, and dark blue indicates $p_{FDR} < 0.05$. (c) Histogram of subject counts by family history-by-sex groups. Sample size: N = 307; FH+ females = 18, FH- females = 99, FH+ males = 23, FH- males = 122.

S11.2 Analyses within-MRI scanner models.

Given the high effect size of MRI model in the ANCOVA models on our NCT metrics, we ran ANCOVA models separately on subjects scanned on each of the three scanner MRI models utilized in our cohort: Siemens Prisma, Siemens Prisma Fit, and GE Discovery MR750. We found that our main results from the main text were driven by subjects scanned on Prisma scanner models (Siemens). In both Siemens models (Prisma and Prisma fit), we find results in mean global TE consistent with our main results, such that FH+ > FH- in females and FH+ < FH- in males. Within GE scanner subjects, mean global TE was also FH+ > FH- females, but differed in males (FH+ > FH-). However, of the three models, only Prisma scanners showed a significant effect of family history-by-sex on mean global TE. At the network level, our main results were partially replicated in Prisma and Prisma Fit scanners. Prisma scanner subjects exhibited significant (post-correction) effects of family history-by-sex on mean network TE of the DMN and DAT, and pre-correction significance in the VIS and SUB. Subjects from Prisma Fit scanners exhibited pre-correction significance in the VAT network. No networks exhibited significant family history-by-sex effects in subjects from GE scanners.

As discussed in the main text, GE scanners had the smallest number of subjects and were made up of younger subjects with higher levels of framewise displacement, lower household income, greater racial/ethnic diversity, and greater family history density of SUD compared to the other two scanner models. The lack of replication of the results from our main text in GE scanner subjects may thus reflect differences in demographics or age-related differences in the neurodevelopmental trajectory. See Table S6 for subject demographics by MRI model. Furthermore, previous ABCD analyses have found GE scanners to have lower-quality data, more confounds, and no real-time motion monitoring[16–19] compared to Siemens.

We also confirmed that clustered brain states are stable across MRI scanners (Siemens vs GE) by examining the centroid similarity of the four brain states in group-average centroids from subjects scanned on GE and Siemens MRI scanners. The results showed high similarity, with Pearson correlation coefficients ranging from r = 0.97 to r = 0.99.

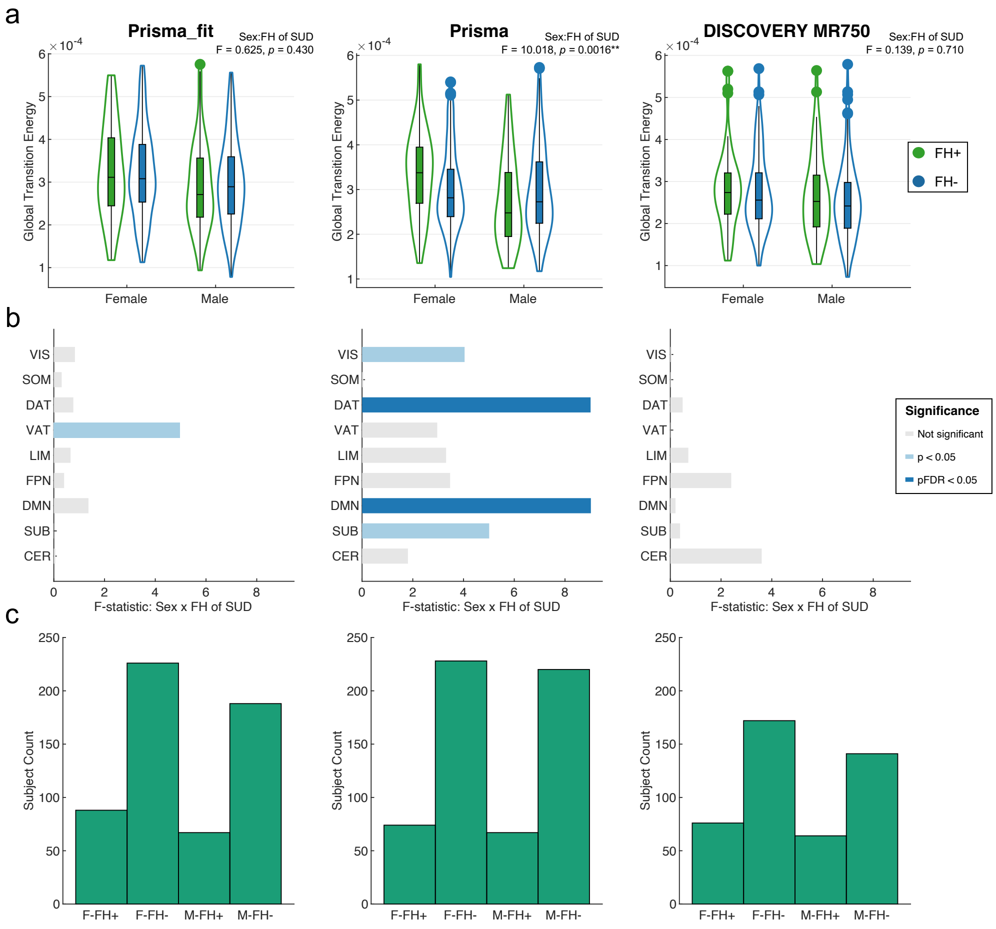

Figure S18. Main results stratified by MRI scanner model (GE Discovery MR750, Siemens Prisma, and Siemens Prisma Fit). (a) Violin plots from each MRI model of mean global TE. The ANCOVA model for global TE revealed a significant (before correction) interaction between sex and family history of SUD. Data distributions are shown with violins; box plots display the median (center line), interquartile range (25th-75th percentiles; box bounds), and whiskers extending to the most extreme values within 1.5×IQR; points beyond the whiskers are plotted as outliers. Green denotes FH+ and blue denotes FH- subjects, separated by sex. (b) ANCOVA F-statistics for the interaction effect between sex and family history of SUD on mean network TE across the nine networks within each MRI model. Grey bars indicate non-significant results, light blue indicates $p < 0.05$, and dark blue indicates $p_{FDR} < 0.05$. (c) Histogram of subject counts by family history-by-sex groups within each MRI model. Total sample: N = 1,611; 238 FH+ females, 626 FH- females, 198 FH+ males, 549 FH- males.

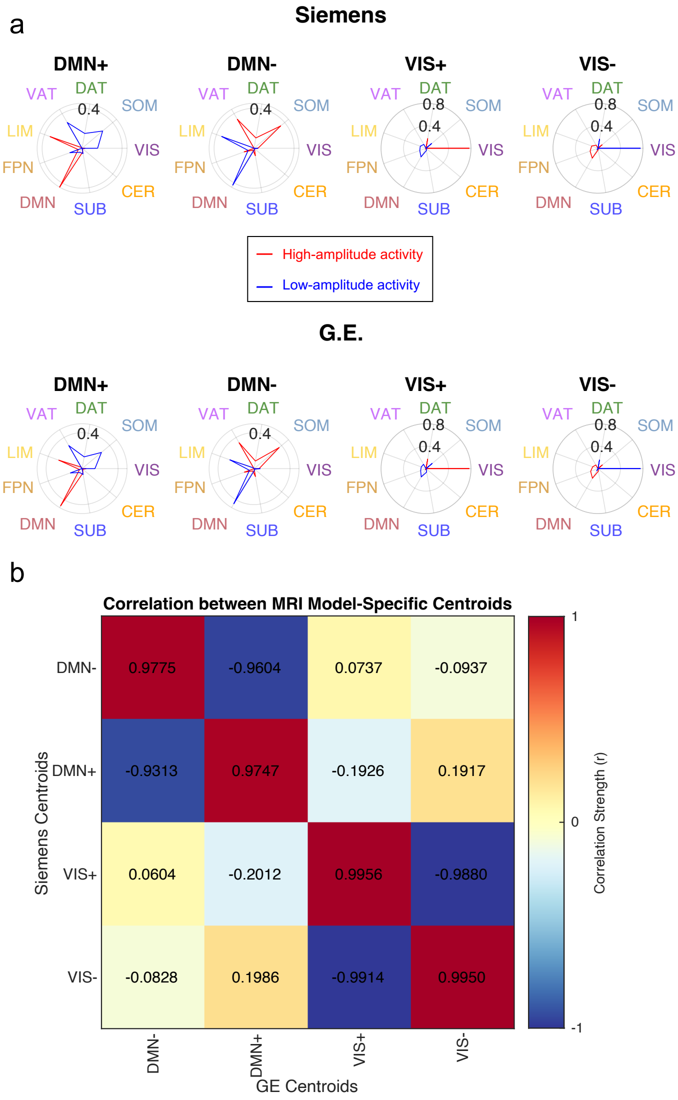

Figure S19. Brain state centroids are consistent across MRI models. (a) Group average centroids for *k*=4 brain states from subjects scanned on a GE or Siemens MRI. Cosine similarity between each state's centroid and canonical resting-state networks (RSNs)[11], computed separately for positive (high-amplitude) and negative (low-amplitude) components. Each state was assigned the RSN label with the maximal similarity, with a sign indicating whether the match was based on high (+) or low (–) amplitude activity. These were consistent across MRI types. (b) Matrix of Pearson correlation values between each pair of brain states from GE and Siemens models. As seen down the diagonal, all states had r > 0.97. SUB = subcortex, CER = cerebellum, VIS = visual network, SOM = somatomotor network, DAT = dorsal attention network, VAT = ventral attention network, LIM = limbic network, FPN = frontoparietal network, DMN = default mode network.

Variable	**GE Discovery MR750** (N = 524)	**Siemens Prisma Fit** (N = 671)	**Siemens Prisma** (N = 691)
**Family History of SUD**			
FH+	140 (26.72%)	155 (23.10%)	141 (20.41%)
FH-	313 (59.73%)	414 (61.70%)	448 (64.83%)
FH+/-	71 (13.55%)	102 (15.20%)	102 (14.76%)
**Sex**			
Male	242 (46.18%)	305 (45.45%)	338 (48.91%)
Female	282 (53.82%)	366 (54.55%)	353 (51.09%)
**Age (Mean ± SD)**	119.10 (±7.59)	120.61 (±7.31)	120.73 (±7.45)
**Framewise Displacement (Mean ± SD)**	0.13 (±0.08)	0.12 (±0.07)	0.11 (±0.07)
**Household Income**			
<$50,000	146 (27.86%)	109 (16.24%)	171 (24.75%)
$50,000 - $100,000	135 (25.76%)	193 (28.76%)	235 (34.01%)
>$100,000	243 (46.37%)	369 (54.99%)	285 (41.24%)
**Parental Education**			
< High School	22 (4.20%)	9 (2.75%)	18 (2.61%)
High School/GED	35 (6.68%)	46 (6.86%)	44 (6.37%)
Some College	63 (12.02%)	60 (13.76%)	59 (8.54%)
Associates/Bachelor	200 (38.17%)	246 (36.66%)	319 (46.16%)
Post-graduate	204 (38.93%)	308 (45.90%)	251 (36.32%)
**Race/Ethnicity**			
White	287 (54.77%)	441 (65.72%)	455 (65.85%)
Black	32 (6.11%)	67 (9.99%)	45 (6.51%)
Hispanic/Latinx	117 (22.33%)	89 (13.26%)	139 (20.12%)
Asian	23 (4.39%)	13 (1.94%)	2 (0.29%)
Other	65 (12.40%)	61 (9.09%)	50 (7.24%)
**Parental Mental Health**			
Yes	250 (47.71%)	326 (48.58%)	326 (47.18%)
No	274 (52.29%)	345 (51.42%)	365 (52.82%)
**Prenatal Substance Exposure**			
Yes	40 (7.63%)	43 (6.41%)	39 (5.64%)
No	484 (92.37%)	628 (93.59%)	652 (94.36%)
**Puberty Status (Male)**			
Pre-Puberty	188 (35.88%)	218 (32.49%)	266 (38.49%)
Early Puberty	42 (8.02%)	67 (9.99%)	61 (8.83%)
Mid/Late Puberty	12 (2.29%)	20 (2.98%)	11 (1.59%)
**Puberty Status (Female)**			
Pre-Puberty	93 (17.75%)	111 (16.54%)	118 (17.08%)
Early Puberty	72 (13.74%)	93 (13.86%)	94 (13.60%)
Mid/Late Puberty	117 (22.33%)	162 (24.14%)	141 (20.41%)
**Family History Density (Mean ± SD)**	0.44 (±0.68)	0.42 (±0.68)	0.34 (±0.57)

Table S6. Demographic data of included subjects by MRI model (GE Discovery MR750, Siemens Prisma Fit, and Siemens Prisma). Categorical variables are displayed as N(%) and continuous variables are displayed as mean (± standard deviation).

S11.3 Analyses within income levels.

Given the significant interaction of family history of SUD and income on global TE, we investigated whether our findings were consistent across all the three income levels: 1, 2, or 3 (low to high). After k-means clustering ($k = 4$) and calculating TE values across the entire cohort, we ran an ANCOVA on data from each income category and display the results here. We included all the same covariates except parental education (due to lack of representation of certain education levels in each income level), and - of course - household income and its interaction with family history of SUD. We find our main results are primarily driven by the largest group of subjects in the high-income category (income group 3), which replicate both global effects (FH+ > FH- females, FH+ < FH- males) and family history-by-sex effects in the DMN and DAT (after correction). Individuals in the income group 1 show the same effect in females (FH+ > FH-) on global TE, but the opposite in males (FH+ > FH-) from results in the main text, and significant (pre-correction) family history-by-sex effects on network TE of VAT and CER. Individuals in income group 2 showed lower mean global TE in males (FH+ < FH-) and slightly lower mean global TE in FH+ females compared to FH- females as well, and no significant family history-by-sex effects in any network. Studies have shown that in the ABCD cohort, which is made up of a majority of those with higher income, individuals from higher income families have higher curiosity and availability to substances, and earlier substance use initiation[20–22], which may be reflected in the stronger manifestation of family history in this group.

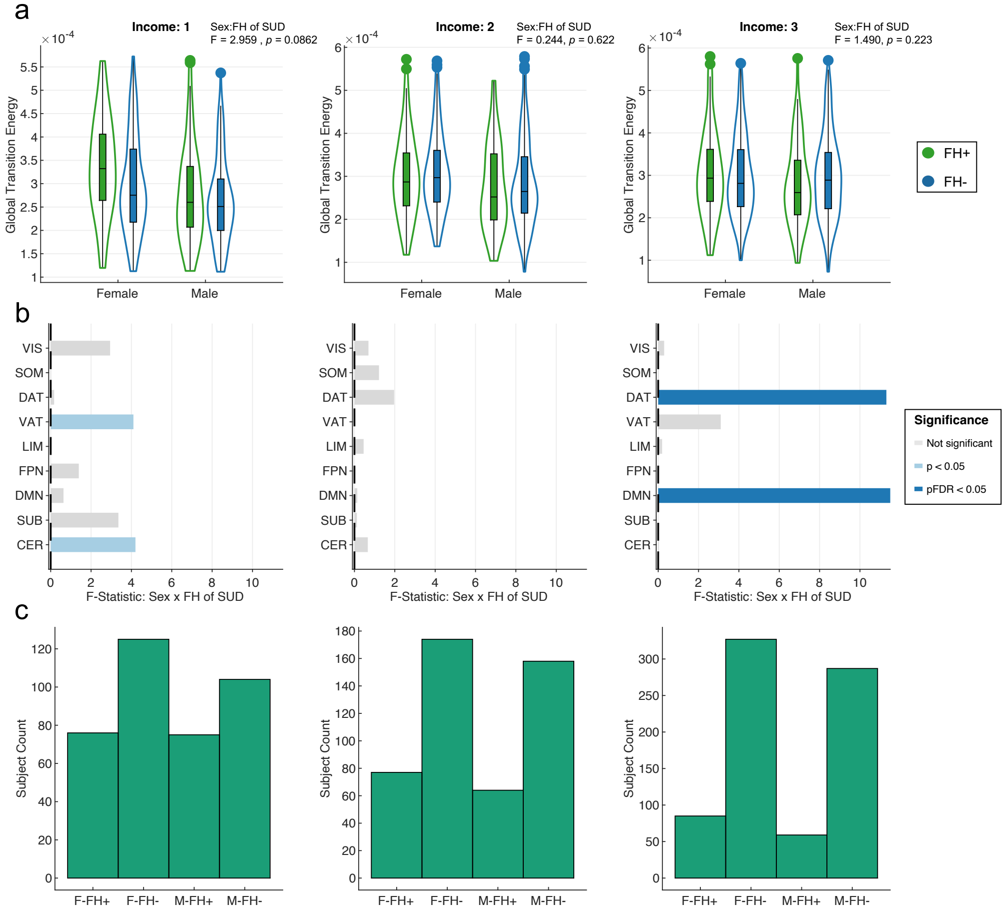

Figure S20. **Mean global and mean network TE results from subjects within household income groups (low = 1, medium = 2, high = 3).** (a) Violin plots of mean global transition energy within each household income. Data distributions are shown with violins; box plots display the median (center line), interquartile range (25th-75th percentiles; box bounds), and whiskers extending to the most extreme values within 1.5×IQR; points beyond the whiskers are plotted as outliers. Green denotes FH+ and blue denotes FH- subjects, separated by sex. (b) ANCOVA F-statistics for the interaction effect between sex and family history of SUD on mean network TE across the nine networks in each income group. Grey bars indicate non-significant results, light blue indicates $p < 0.05$, and dark blue indicates $p_{\mathrm{FDR}} < 0.05$. (c) Histogram of subject counts by family history-by-sex groups.

S12 Future substance use and behavioral risk factors.

S12.1 Future substance use.

We analyzed how our NCT results relate to follow-up substance use (SU) by looking at FH+ subjects from the same ABCD cohort who reported substance use at any of the annual follow-ups (1, 2, or 3-year follow-ups) in the `su_y_sui` instrument. We used FH+ individuals only as to better isolate the specific mechanisms of substance use initiation in the cohort with a family history. 4-year follow-up was not utilized due to missingness in many subjects. Subjects who used substances at any of these assessments were coded as follow-up SU = 1 and participants who reported no substance use at all time points were coded follow-up SU = 0. Substance use was considered a sip or more of alcohol, a puff or more of nicotine (including cigarettes, ENDS, smokeless tobacco, cigars, hookah, pipe, nicotine replacement) products, cannabis (including smoked flower, blunts, vaped flower, edibles, vaped concentrates, smoked concentrates, THC-infused alcohol, tinctures, synthetic THC), recreational drugs (amphetamine, tranq, vicodin, cough syrup, or steroids), hallucinogens (LSD, psilocybin, shrooms, salvia), non-medical use of prescription drugs or other drugs. Subjects who were missing follow-up data and did not respond yes to any other assessments were excluded. Given the various pathways that may lead an individual to problematic substance use, we limited our analysis to individuals with family history of SUD in order to specifically relate neural manifestation of family history to follow up substance use. To increase sample size, we included FH+/- individuals (at least one grandparent with SUD) alongside FH+ youth. This resulted in a final cohort of N = 629 of which N = 111 (17.6%) had used substances.

We conducted an ANCOVA on baseline global TE, controlling for sex, age, follow-up substance use, framewise displacement, MRI model, income, parental education, race, prenatal substance exposure, parental mental health, puberty, and interactions of sex and puberty, sex and follow-up SU, and follow-up SU and income. Baseline mean global TE was not significantly associated with follow-up SU ($F < 0.0001$, $p = 0.99$, $\eta_p^2 < 0.0001$), nor sex*follow-up SU ($F = 1.22$, $p = 0.27$, $\eta_p^2 = 0.0018$). Within-sex t-tests revealed no significant differences in baseline mean global TE between those who did and did not report follow-up SU (females: $t = 0.032$, $p = 0.975$, Cohen's $d = 0.005$; males: $t = -0.520$, $p = 0.603$, Cohen's $d = -0.076$). In network-specific ANCOVA models, follow-up SU was not significantly associated with any network. However, the interaction of sex and follow-up SU was significant for mean VAT TE (pre-correction) and mean DMN TE was second largest F-statistic but was sub-significant. Post-hoc t-tests found this was driven by a decrease in mean VAT TE in FH+ males who use substances (significant pre-correction) and increased in mean DMN TE in FH+ females who use substances at follow up (not significant).

These trends align with our main findings of sex-specific dynamics of DMN and attentional networks predispose FH+ youth to future SU. However, the small sample size and limited measures of severity of substance use at follow-up constrain definitive conclusions. While early substance use often predicts later problematic use, its risk level remains difficult to assess (e.g., does a single sip of alcohol indicate meaningful risk?). Additionally, it is possible some neurophenotypes of FH+ confer resiliency to SUD. Further research comparing these pathways and analyzing larger longitudinal datasets (e.g., future ABCD waves) will be crucial.

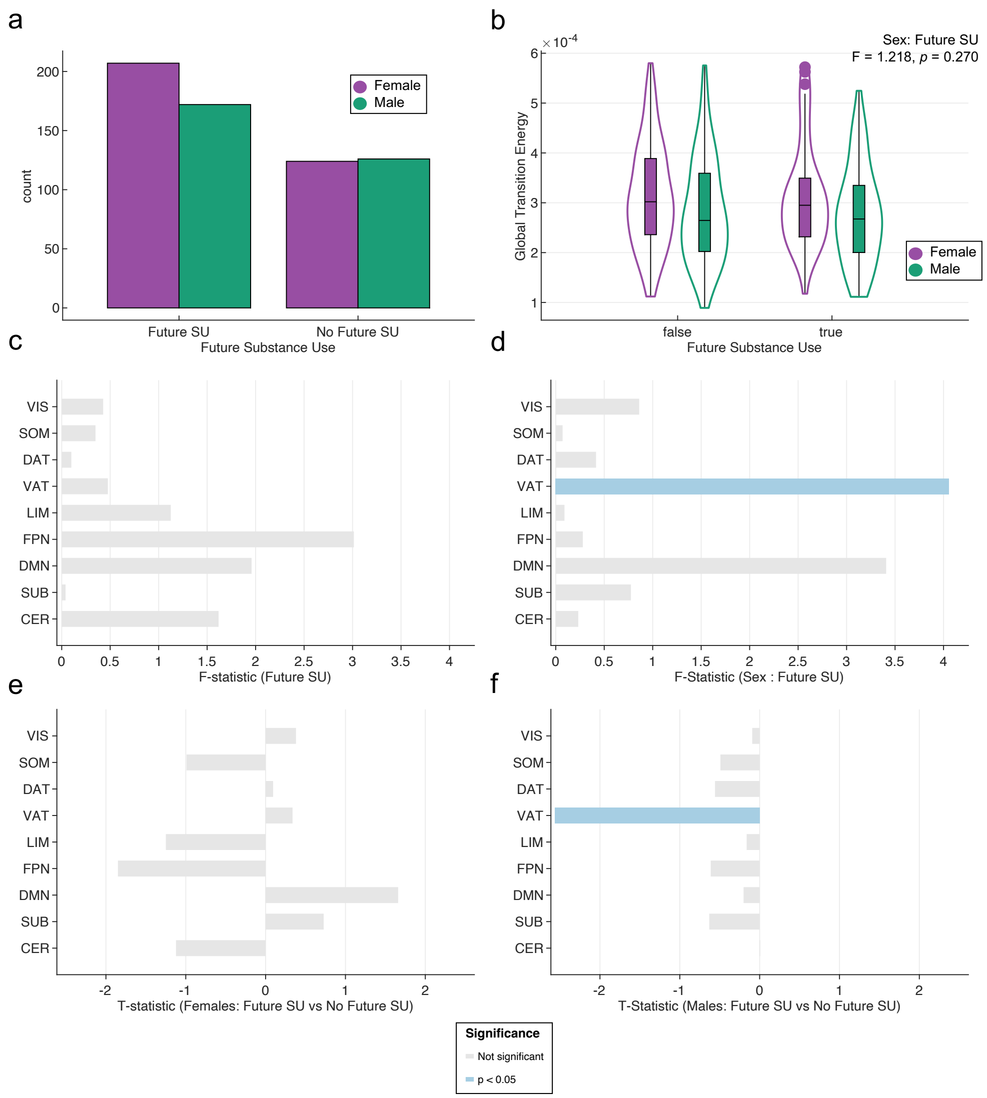

Figure S21. Comparisons of ABCD youth with a family history of SUD (either FH+ or FH+/-) who did versus did not use substances by the third year follow-up assessment. (a) Histogram of subject count of each family history-by-sex groups of those who did and did not use substances at follow-up. (b) Across-sex and within-sex comparisons in mean global TE were not significantly different between those with and without follow-up SU. Violin plots of mean global transition energy within each household income. Data distributions are shown with violins; box plots display the median (center line), interquartile range (25th–75th percentiles; box bounds), and whiskers extending to the most extreme values within 1.5×IQR; points beyond the whiskers are plotted as outliers. Green denotes males and purple denotes females subjects, separated by future substance use. (c-d) Bar plots of F-statistics from ANCOVA analysis of mean network TE, revealing no significant effect of future substance use in any network (c) and a significant (before correction) effect on VAT TE for the interaction of sex and future substance use (d). (e-f) T-statistics from within-sex, unpaired t-tests (two-sided) of mean network TE between those with and without future SU. This found group-by-sex effects in VAT were driven by decreased VAT in males with future SU. Mean DMN TE was greater in females with future SU compared to females without follow up, but this was not significant. For (c-f), grey bars indicate non-significant results and light blue indicates $p < 0.05$.

S12.2 Behavioral risk factors.

We explored the relationship between the observed increases/decreases in global and network TE values and known behavioral and mental health risk factors of SUD. We chose the following factors associated with risk of SUD[23–25].

- **Childhood Behavioral CheckList (CBCL)**: we utilized summary scores from 11 syndrome scales from the `abcd_cbcls01` instrument: externalizing, internalizing, withdrawal-depression, anxious-depression, attention, somatic, social, thought, rule-breaking, aggression, and total problems[26].
- **Behavioral Inhibition/Behavioral Activation Scale (BIS/BAS)**: from the `mh_y_bisbas` instrument, we used the summary score from the BIS questionnaire (`bis_y_ss_bis_sum`) and the summary score for each of three BAS subscales: Reward Responsiveness (`bis_y_ss_bas_rr`), Drive (`bis_y_ss_bas_drive`), and Fun-Seeking (`bis_y_ss_bas_fs`)[27].
- **UPPS-P Impulsive Behavior Scale (UPPS-P)**: the `mh_u_upps` instrument assesses five facets of impulsivity: lack of premeditation, lack of perseverance, negative urgency, sensation seeking and positive urgency[28].

All measures were coded such that a higher score reflects a potential higher risk in SUD. To this end, we took the negative of BIS summary score (i.e., higher BIS score = less inhibition = higher SUD risk).

To further evaluate whether the observed associations between transition energies and behavioral measures may reflect group-level differences, we ran follow-up ANCOVA models that - in addition to all covariates used in the main analysis - included each behavior and the interaction terms for behavior $\times$ family history of SUD, behavior $\times$ sex, and the three-way interaction of behavior $\times$ sex $\times$ family history of SUD. These models were run for each behavior and each level of TE (global and networks) separately. We applied Benjamini-Hochberg correction for multiple comparisons across all behavioral terms within each TE outcome, separately for each effect of interest (main effect, two-way interactions, and three-way interaction). Results are summarized below in heatmaps of F-statistics with significance annotations (* = $p < 0.05$; ** = pFDR < 0.05). This approach allowed us to test whether behavioral associations with TE varied by family history of SUD status and/or sex, capturing potential moderating effects that may have been obscured in the combined-group correlations.

None of the behavioral measures showed a significant effect of the interaction of behavior $\times$ family history of SUD on global TE. However, global TE exhibited significant (pre-correction) interaction effects of behavior and sex for three behaviors: rule-breaking (driven by FPN, DMN, VAT, and LIM), social problems (driven by FPN, VAT, SUB, and DMN), and sensation seeking (driven by DAT). The FPN network showed the strongest interaction effects of behavior and sex, particularly for externalizing, social, rule-breaking, aggression, and total problem behavioral scores (all significant post-correction). We also observed a significant (pre-correction) three-way interaction between sex $\times$ family history of SUD $\times$ BAS reward on global TE, with effects most pronounced in the VIS network. Indeed, the Spearman rank correlation between BAS reward and global TE varied by family history group and sex (FH+ females: rho = 0.04, $p = 0.56$; FH- females: rho = -0.04, $p = 0.36$; FH+ males: rho = -0.05, $p = 0.53$; and FH- males: rho = 0.04, p= 0.32), though all of these effects were weak and non-significant

Additionally, rule-breaking behavior showed a significant main effect on global TE, with particularly strong associations in the SOM network. The SOM network also showed significant (post-correction) effects of UPPS-P lack of perseverance, and of three-way interaction between BAS drive $\times$ sex $\times$ family history. The correlation between BAS drive and SOM TE varied by group (FH+ females: r = 0.02 , $p = 0.77$; FH- females: rho = -0.02, p = 0.61; FH+ males: rho = -0.07, $p = 0.30$; and FH- males: rho = 0.01, $p = 0.80$), but were weak and non-significant.

Overall, these findings suggest that the sex differences reported in the main text are not driven by family history group status. Notably, transition energies in the DMN, VAT, and DAT networks — those identified as altered by an interaction of sex and family history of SUD — were associated with different behavioral variables in males and females. The sex-specific and sex-general associations between behavior and TE observed here further support our conclusion that neurophenotypes associated with a family history of SUD likely exhibit overlapping but distinct patterns in males and females, with corresponding behavioral manifestations. The results highlight complex interactions among family history, sex, and behavior that may be valuable for future research.

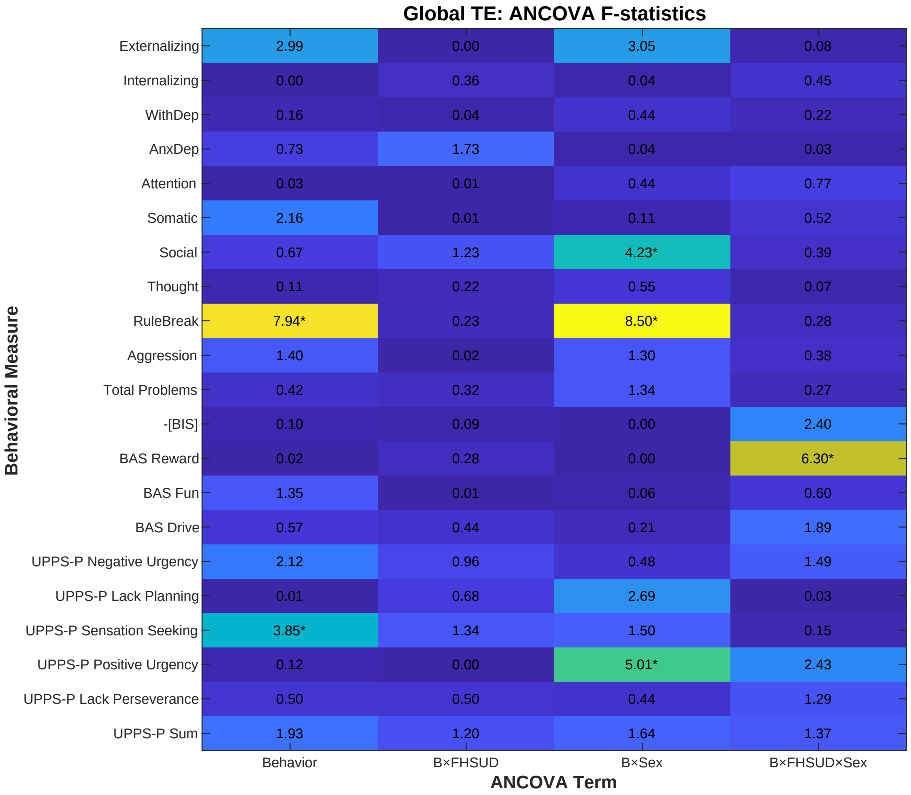

Figure S22. Each cell reflects the F-statistic for the main effect of behavior (B), behavior × family history SUD interaction, behavior × sex interaction, or the three-way interaction behavior × sex × family history of SUD on global transition energy (TE) across 21 behavioral and psychological measures. Significance is indicated with * ($p < 0.05$) and ** (pFDR < 0.05) based on Benjamini-Hochberg correction applied across behavioral terms within each effect.

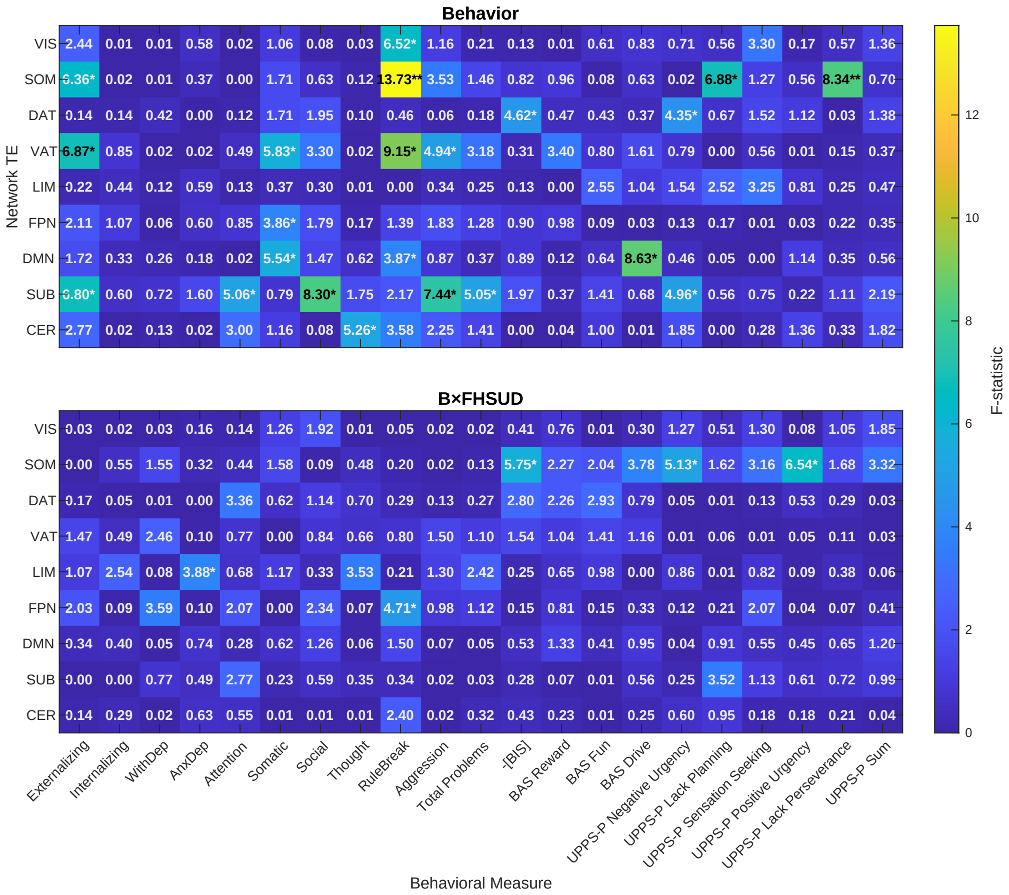

Figure S23. Each cell reflects the F-statistic for the main effect of behavior (top) and behavior × family history SUD interaction (bottom) on each of nine network's transition energies (TE) across 21 behavioral and psychological measures. Significance is indicated with * ($p < 0.05$) and ** (pFDR < 0.05) based on Benjamini-Hochberg correction applied across all behavioral terms within each effect.

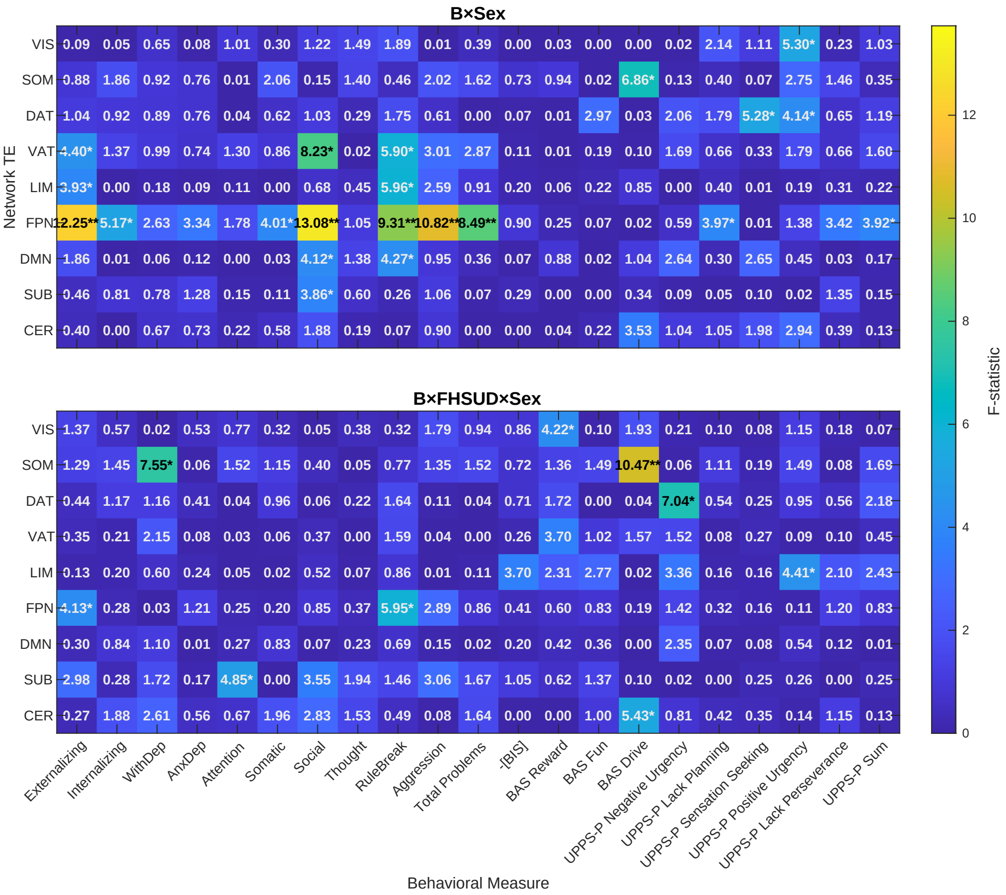

Figure S24. Each cell reflects the F-statistic for the effect of behavior (B) × sex interaction (top), and the three-way interaction behavior × sex × family history of SUD (bottom) on each network's transition energy (TE) across 21 behavioral and psychological measures. Significance is indicated with * ($p < 0.05$) and ** (pFDR < 0.05) based on Benjamini-Hochberg correction applied across behavioral terms within each effect.

Next, given the observed effects of the interaction between behavior and sex on TE, we performed Spearman's rank correlations between these baseline behavioral scores and baseline mean global/network TE values separately for females and males. We then applied the Benjamini-Hochberg procedure to all *p*-values within each sex.

In females, mean global TE showed significant (post-correction) positive correlations with CBCL Externalizing and Rule-Breaking sub-scales. Externalizing symptoms were also significantly associated with mean FPN TE (post-correction) and with VIS, LIM, and VAT network TEs (pre-correction). Rule-Breaking behavior was significantly correlated with VIS, SOM, and VAT TEs (post-correction), and with FPN and DMN TEs (pre-correction). Additionally, mean DMN TE—which was elevated in FH+ compared to FH– females—was positively correlated (pre-correction) with the CBCL Somatic and Social Problems sub-scales. Somatic symptoms, as a subtype of internalizing problems, may signal heightened sensitivity to negative reinforcement, a proposed mechanism linking distress to substance use according to the self-medication hypothesis[29,30]. Interestingly, DMN TE showed a positive association with somatic symptoms in both males and females (pre-correction), suggesting a possible sex-general behavioral correlate of brain dynamics more prominently expressed in FH+ females.

In males, neither mean global nor mean network TE values showed significant correlations with behavioral or psychological measures after correction. The plots below display only correlations that survived correction (pFDR < 0.05). Before correction, lower mean network TE in the DAT (FH+ < FH- males) was associated with higher scores on the BAS Fun and Drive sub-scales, as well as the UPPS-P Lack of Planning and Positive Urgency sub-scales. Lower mean VAT TE (FH+ < FH- males) was similarly associated with higher UPPS-P Positive Urgency scores. Overall, these findings suggest that lower mean network TE in the VAT and DAT may relate to greater impulsivity and sensitivity to positive reinforcement.

Overall, we observed a pattern of more negative correlations between mean network TE and behavioral/ psychological risk factors for SUD in males, whereas females showed more positive correlations. We did not observe the expected strong associations between mean network TE and internalizing or externalizing symptoms (e.g., higher mean DMN TE with greater internalizing in females, and lower mean DAT/VAT TE with greater externalizing in males). However, we found some evidence linking these networks to reinforcement processes: in males, mean DAT and VAT TE were associated with positive reinforcement (UPPS-P Positive Urgency), while in females, mean DMN TE was associated with negative reinforcement (somatic symptoms). Externalizing symptoms tend to be higher in males during childhood and converge with females later, whereas internalizing symptoms are typically higher in females than males by mid-adolescence[31]. Thus, the mean global/network TE differences observed in FH+ females at baseline may reflect an underlying neural predisposition to both internalizing disorders and SUD, which could become clearer as this cohort matures.

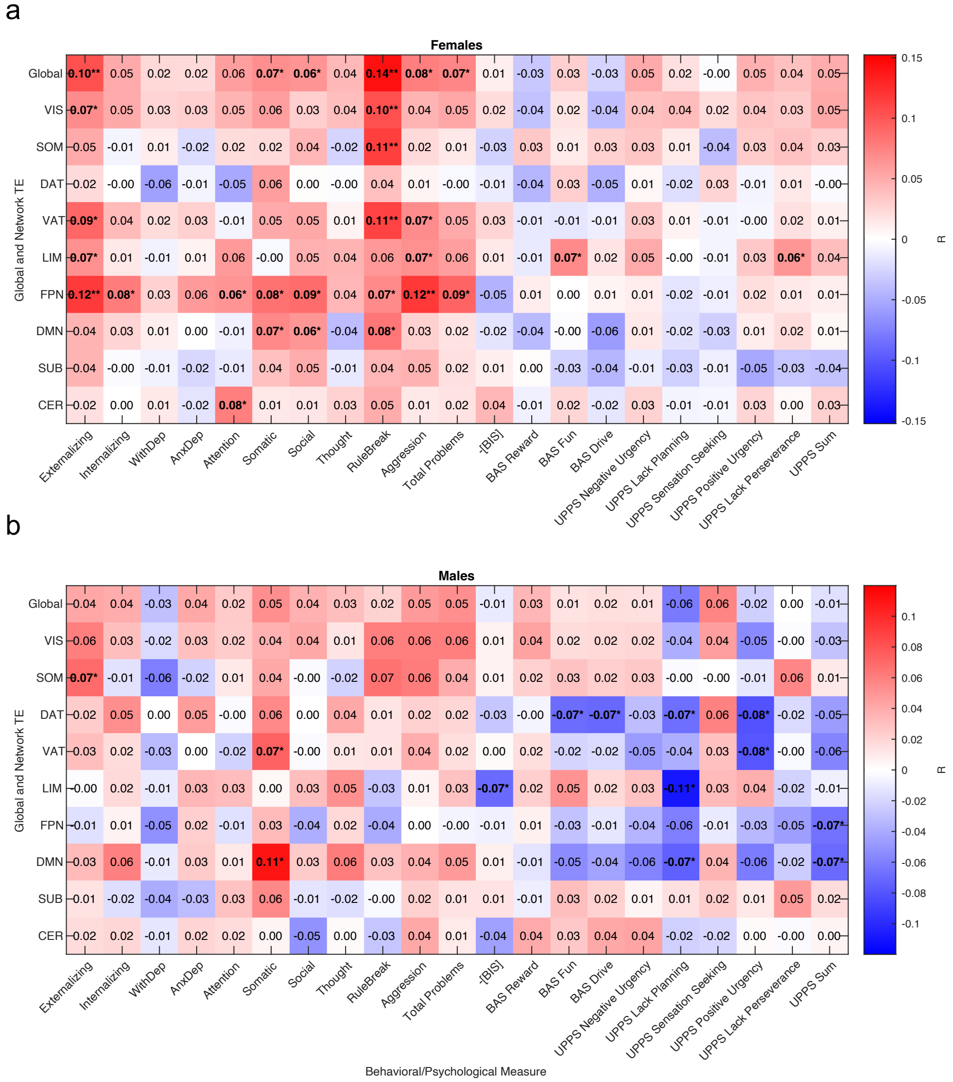

Figure S25. Associations between CBCL, BIS/BAS and UPPS-P scores (x-axis) and mean global/network TE measures (y-axis) in males (a) and females (b) separately - regardless of family history group. The color bar represents the correlation strength (rho) calculated by performing Spearman's rank correlations. One asterisk (*) indicates p<0.05, and two asterisks (**) indicate pFDR < 0.05.

References

1. Chen, J. *et al.* Shared and unique brain network features predict cognitive, personality, and mental health scores in the ABCD study. *Nat. Commun.* **13**, 2217, DOI: 10.1038/s41467-022-29766-8 (2022). Number: 1 Publisher: Nature Publishing Group.
2. Ooi, L. Q. R. *et al.* Comparison of individualized behavioral predictions across anatomical, diffusion and functional connectivity MRI. *NeuroImage* **263**, 119636, DOI: 10.1016/j.neuroimage.2022.119636 (2022).
3. Roffman, J. L. *et al.* Association of adverse prenatal exposure burden with child psychopathology in the Adolescent Brain Cognitive Development (ABCD) Study. *PLOS ONE* **16**, e0250235, DOI: 10.1371/journal.pone.0250235 (2021). Publisher: Public Library of Science.
4. Zhang, R., Manza, P. & Volkow, N. D. Prenatal caffeine exposure: association with neurodevelopmental outcomes in 9- to 11-year-old children. *J. Child Psychol. Psychiatry* **63**, 563–578, DOI: 10.1111/jcpp.13495 (2022). _eprint: https://onlinelibrary.wiley.com/doi/pdf/10.1111/jcpp.13495.
5. Christensen, Z. P., Freedman, E. G. & Foxe, J. J. Caffeine exposure *in utero* is associated with structural brain alterations and deleterious neurocognitive outcomes in 9–10 year old children. *Neuropharmacology* **186**, 108479, DOI: 10.1016/j.neuropharm.2021.108479 (2021).
6. Hatoum, A. S. *et al.* Multivariate genome-wide association meta-analysis of over 1 million subjects identifies loci underlying multiple substance use disorders. *Nat. mental health* **1**, 210–223, DOI: 10.1038/s44220-023-00034-y (2023).
7. Stubbs, J. L. *et al.* Heterogeneous neuroimaging findings across substance use disorders localize to a common brain network. *Nat. Mental Heal.* **1**, 772–781, DOI: 10.1038/s44220-023-00128-7 (2023). Publisher: Nature Publishing Group.
8. Cornblath, E. *et al.* Temporal sequences of brain activity at rest are constrained by white matter structure and modulated by cognitive demands. *Commun. Biol.* **3**, 261, DOI: 10.1038/s42003-020-0961-x (2020).
9. Singleton, S. P. *et al.* Receptor-informed network control theory links LSD and psilocybin to a flattening of the brain's control energy landscape. *Nat. Commun.* **13**, 5812, DOI: 10.1038/s41467-022-33578-1 (2022).
10. Goutte, C., Toft, P., Rostrup, E., Nielsen, F. A. & Hansen, L. K. On Clustering fMRI Time Series. *NeuroImage* **9**, 298–310, DOI: 10.1006/nimg.1998.0391 (1999).
11. Thomas Yeo, B. T. *et al.* The organization of the human cerebral cortex estimated by intrinsic functional connectivity. *J. Neurophysiol.* **106**, 1125–1165, DOI: 10.1152/jn.00338.2011 (2011). Publisher: American Physiological Society.
12. Brown, S. A. *et al.* The National Consortium on Alcohol and NeuroDevelopment in Adolescence (NCANDA): A Multisite Study of Adolescent Development and Substance Use. *J. Stud. on Alcohol Drugs* **76**, 895–908, DOI: 10.15288/jsad.2015.76.895 (2015).
13. Müller-Oehring, E. M. *et al.* Influences of Age, Sex, and Moderate Alcohol Drinking on the Intrinsic Functional Architecture of Adolescent Brains. *Cereb. Cortex (New York, NY)* **28**, 1049–1063, DOI: 10.1093/cercor/bhx014 (2018).
14. Rohlfing, T., Zahr, N. M., Sullivan, E. V. & Pfefferbaum, A. The SRI24 multichannel atlas of normal adult human brain structure. *Hum. Brain Mapp.* **31**, 798–819, DOI: 10.1002/hbm.20906 (2010).
15. Cetin-Karayumak, S. *et al.* Harmonized diffusion MRI data and white matter measures from the Adolescent Brain Cognitive Development Study. *bioRxiv: The Prepr. Serv. for Biol.* 2023.04.04.535587, DOI: 10.1101/2023.04.04.535587 (2023).
16. Pan, Y. *et al.* A Site-Wise Reliability Analysis of the ABCD Diffusion Fractional Anisotropy and Cortical Thickness: Impact of Scanner Platforms. *Hum. Brain Mapp.* **45**, e70070, DOI: 10.1002/hbm.70070 (2024). _eprint: https://onlinelibrary.wiley.com/doi/pdf/10.1002/hbm.70070.

17. Hagler, D. J. *et al.* Image processing and analysis methods for the Adolescent Brain Cognitive Development Study. *NeuroImage* **202**, 116091, DOI: 10.1016/j.neuroimage.2019.116091 (2019).

18. Nielson, D. M. *et al.* Detecting and harmonizing scanner differences in the ABCD study - annual release 1.0, DOI: 10.1101/309260 (2018). Pages: 309260 Section: New Results.

19. Sinha, H. & Raamana, P. R. Solving the Pervasive Problem of Protocol Non-Compliance in MRI using an Open-Source tool mrQA. *Neuroinformatics* DOI: 10.1007/s12021-024-09668-4 (2024).

20. Lisdahl, K. M. *et al.* Substance use patterns in 9-10 year olds: Baseline findings from the adolescent brain cognitive development (ABCD) study. *Drug Alcohol Dependence* **227**, 108946, DOI: 10.1016/j.drugalcdep.2021.108946 (2021).

21. Martz, M. E. *et al.* Individual-, peer-, and parent-level substance use-related factors among 9- and 10-year-olds from the ABCD Study: Prevalence rates and sociodemographic differences. *Drug Alcohol Dependence Reports* **3**, 100037, DOI: 10.1016/j.dadr.2022.100037 (2022).

22. Sullivan, R. M. *et al.* Substance use patterns in 9 to 13-year-olds: Longitudinal findings from the Adolescent Brain Cognitive Development (ABCD) study. *Drug Alcohol Dependence Reports* **5**, 100120, DOI: 10.1016/j.dadr.2022.100120 (2022).

23. Ferdinand, R. F., Blüm, M. & Verhulst, F. C. Psychopathology in adolescence predicts substance use in young adulthood. *Addict. (Abingdon, England)* **96**, 861–870, DOI: 10.1046/j.1360-0443.2001.9668617.x (2001).

24. Gonçalves, P. D. *et al.* Associations Between Family History of Alcohol and/or Substance Use Problems and Frontal Cortical Development From 9 to 13 Years of Age: A Longitudinal Analysis of the ABCD Study. *Biol. Psychiatry Glob. Open Sci.* **4**, 100284, DOI: 10.1016/j.bpsgos.2023.100284 (2024).

25. Rømer Thomsen, K. *et al.* Impulsivity traits and addiction-related behaviors in youth. *J. Behav. Addict.* **7**, 317–330, DOI: 10.1556/2006.7.2018.22 (2018).

26. Achenbach, T. M. & Ruffle, T. M. The Child Behavior Checklist and Related Forms for Assessing Behavioral/Emotional Problems and Competencies. *Pediatr. In Rev.* **21**, 265–271, DOI: 10.1542/pir.21.8.265 (2000). Publisher: American Academy of Pediatrics (AAP).

27. Carver, C. S. & White, T. L. Behavioral inhibition, behavioral activation, and affective responses to impending reward and punishment: The BIS/BAS Scales. *J. Pers. Soc. Psychol.* **67**, 319–333, DOI: 10.1037/0022-3514.67.2.319 (1994). Place: US Publisher: American Psychological Association.

28. Whiteside, S. P. & Lynam, D. R. The Five Factor Model and impulsivity: Using a structural model of personality to understand impulsivity. *Pers. Individ. Differ.* **30**, 669–689, DOI: 10.1016/S0191-8869(00)00064-7 (2001). Place: Netherlands Publisher: Elsevier Science.

29. Broman, C. L., Wright, M. K., Broman, M. J. & Bista, S. Self-Medication -and Substance Use: A Test of the Hypothesis. *J. Child & Adolesc. Subst. Abus.* **28**, 494–504, DOI: 10.1080/1067828X.2020.1789526 (2019). Publisher: Taylor & Francis _eprint: https://doi.org/10.1080/1067828X.2020.1789526.

30. Meneo, D. *et al.* A systematic review and meta-analysis of the association between young adults' sleep habits and substance use, with a focus on self-medication behaviours. *Sleep Medicine Rev.* **70**, 101792, DOI: 10.1016/j.smrv.2023.101792 (2023).

31. Ho, T. C., Buthmann, J., Chahal, R., Miller, J. G. & Gotlib, I. H. Exploring sex differences in trajectories of pubertal development and mental health following early adversity. *Psychoneuroendocrinology* **161**, 106944, DOI: 10.1016/j.psyneuen.2023.106944 (2024).
