# Supplementary material for: Sex-specific differences in brain activity dynamics of youth with a family history of substance use disorder
Source: Nat Ment Health. 2025 Nov 21;3(12):1493–511. doi: 10.1038/s44220-025-00523-2 (PMC12705459; doi:10.1038/s44220-025-00523-2)
Supplement: Supplementary file 2 — Reporting Summary [file 44220_2025_523_MOESM2_ESM.pdf]

nature portfolio

Corresponding author(s): Louisa Schilling

Last updated by author(s): Apr 9, 2025

Reporting Summary

Nature Portfolio wishes to improve the reproducibility of the work that we publish. This form provides structure for consistency and transparency in reporting. For further information on Nature Portfolio policies, see our Editorial Policies and the Editorial Policy Checklist.

Statistics

For all statistical analyses, confirm that the following items are present in the figure legend, table legend, main text, or Methods section.

n/a	Confirmed	
☐	☒	The exact sample size (*n*) for each experimental group/condition, given as a discrete number and unit of measurement
☐	☒	A statement on whether measurements were taken from distinct samples or whether the same sample was measured repeatedly
☐	☒	The statistical test(s) used AND whether they are one- or two-sided<br>*Only common tests should be described solely by name; describe more complex techniques in the Methods section.*
☐	☒	A description of all covariates tested
☐	☒	A description of any assumptions or corrections, such as tests of normality and adjustment for multiple comparisons
☐	☒	A full description of the statistical parameters including central tendency (e.g. means) or other basic estimates (e.g. regression coefficient) AND variation (e.g. standard deviation) or associated estimates of uncertainty (e.g. confidence intervals)
☐	☒	For null hypothesis testing, the test statistic (e.g. *F*, *t*, *r*) with confidence intervals, effect sizes, degrees of freedom and *P* value noted<br>*Give P values as exact values whenever suitable.*
☒	☐	For Bayesian analysis, information on the choice of priors and Markov chain Monte Carlo settings
☒	☐	For hierarchical and complex designs, identification of the appropriate level for tests and full reporting of outcomes
☐	☒	Estimates of effect sizes (e.g. Cohen's *d*, Pearson's *r*), indicating how they were calculated

Our web collection on statistics for biologists contains articles on many of the points above.

Software and code

Policy information about availability of computer code

Data collection	No software was used for data collection since all analyses used existing data resources. Data collection and pre-processing was performed as described elsewhere for ABCD (Casey et al., 2018, Dev Cogn Neurosci; Ooi, et al., 2022, NeuroImage) and NCANDA (Brown, et al., 2015, Journal of studies on alcohol and drugs).
Data analysis	This project utilized publicly available code developed by Cornblath et al. (2020) and Singleton et al. (2022). Custom scripts were developed using MATLAB R2023a and Python 3.11. MATLAB visualizations were generated using the gramm toolbox (v2.25; https://github.com/piermorel/gramm). Python visualizations were rendered using brainmontageplot (v1.4.2; https://github.com/kjamison/brainmontageplot). All code is available on GitHub: https://github.com/louisaschill/abcd_fhsud_nct.

For manuscripts utilizing custom algorithms or software that are central to the research but not yet described in published literature, software must be made available to editors and reviewers. We strongly encourage code deposition in a community repository (e.g. GitHub). See the Nature Portfolio guidelines for submitting code & software for further information.

Data

Policy information about availability of data

All manuscripts must include a data availability statement. This statement should provide the following information, where applicable:

- Accession codes, unique identifiers, or web links for publicly available datasets
- A description of any restrictions on data availability
- For clinical datasets or third party data, please ensure that the statement adheres to our policy

Data used in this article were obtained from the Adolescent Brain Cognitive Development (ABCD) Study (https://abcdstudy.org), held in the NIMH Data Archive (NDA). This is a multisite, longitudinal study designed to recruit more than 10,000 children age 9-10 and follow them over 10 years into early adulthood. The ABCD Study® is supported by the National Institutes of Health and additional federal partners under award numbers U01DA041048, U01DA050989, U01DA051016, U01DA041022, U01DA051018, U01DA051037, U01DA050987, U01DA041174, U01DA041106, U01DA041117, U01DA041028, U01DA041134, U01DA050988, U01DA051039, U01DA041156, U01DA041025, U01DA041120, U01DA051038, U01DA041148, U01DA041093, U01DA041089, U24DA041123, U24DA041147. A full list of supporters is available at https://abcdstudy.org/federal-partners.html. A listing of participating sites and a complete listing of the study investigators can be found at https://abcdstudy.org/consortium_members/. ABCD consortium investigators designed and implemented the study and/or provided data but did not necessarily participate in the analysis or writing of this report. This manuscript reflects the views of the authors and may not reflect the opinions or views of the NIH or ABCD consortium investigators. The ABCD data repository grows and changes over time. The processed neuroimaging data by (Chen et al., 2022; Ooi et al., 2022) used in this study was uploaded by the original authors to the NDA. Researchers with access to the ABCD data will be able to download the data: https://nda.nih.gov/study.html?id=1368. The ABCD imaging data used in this report came from https://doi.org/10.15154/1504041 and non-imaging data was from the 5.1 release (http://dx.doi.org/10.15154/z563-zd24). These data were used in the analyses described in https://doi.org/10.15154/c9z7-ng36.

Collection and distribution of the NCANDA data were supported by NIH funding AA021681, AA021690, AA021691, AA021692, AA021695, AA021696, AA021697. Researchers with access to the NCANDA data will be able to download the data via https://nda.nih.gov/study.html?id=4513.

Research involving human participants, their data, or biological material

Policy information about studies with human participants or human data. See also policy information about sex, gender (identity/presentation), and sexual orientation and race, ethnicity and racism.

Reporting on sex and gender	In both the main analysis in ABCD and in the replication in NCANDA, we focused on how sex modulates the effect of family history of substance use disorder (SUD), and accordingly, examined the interaction between sex and family history throughout our analyses. All ANCOVA models included both sex and the sex-by-family-history interaction term. To assess the directionality of effects within each sex, we conducted within-sex t-tests comparing youth with (FH+) and without (FH–) a family history of SUD. We did not explicitly examine gender, given the limited gender identity diversity in this relatively young cohort (ages 9–11). However, we acknowledge that this is a limitation, as sex and gender may have distinct neural correlates. We discuss these limitations in greater detail in the Discussion section of the main text. Sex was determined based on parent-reported biological sex assigned at birth and confirmed via salivary DNA testing.
Reporting on race, ethnicity, or other socially relevant groupings	ABCD Study: We included race/ethnicity as a covariate in all ANCOVA models to account for potential confounding effects on neuro-developmental outcomes. In the ABCD Study, race/ethnicity was parent- or guardian-reported at baseline using predefined categories provided by the study protocol (White, Black, Hispanic/Latinx, Asian or Other). We used household income and parental education for proxies of socioeconomic status. We recognize that race/ethnicity are socially constructed categories rather than biological ones, and we included them to account for the structural and social inequities that may influence brain development and related outcomes. By including these variables as covariates, we aimed to statistically reduce potential bias in estimating the effects of family history of substance use disorder and its interaction with sex on brain dynamics.<br><br>NCANDA Study:
Population characteristics	ABCD Study: We utilized data from the baseline assessment of a large sample of substance-naive youth (N = 1886 individuals, ages 10.02 ± 0.62, 53% female). We classify individuals as FH+ if they have at least one parent or two grandparents with a history of SUD and FH- if no parents or grandparents with a history. Individuals with just one grandparent with a history of SUD are classified as FH+/- and are included only in analyses of continuous associations (i.e., family history density). Demographic comparisons indicated no significant differences between family history groups in terms of sex, age, framewise displacement, or MRI scanner model distribution. However, FH+ individuals tended to have lower household income, greater racial/ethnic diversity, lower parental education, increased prenatal substance exposure, more parental mental health issues, and a more advanced pubertal stage.<br><br>NCANDA Study:
Recruitment	ABCD Study: Data Adult participants consented to participating, and minors provided written assent along with consent from a parent/legal guardian. Participants were compensated for completing all baseline sessions, and parents were compensated for completing the baseline interview, with total compensation ranging from \$200 to \$225 per family across sites. Participants discovered to be ineligible received partial compensation based on the measures completed before exclusion. used in the preparation of this article were obtained from the Adolescent Brain Cognitive Development (ABCD) Study (https://abcdstudy.org), held in the NIMH Data Archive (NDA). This is a multi-site, longitudinal study designed to recruit more than 10,000 children age 9-10 and follow them over 10 years into early adulthood. All parents or legal guardians provided written informed consent prior to participation in the study, and children provided verbal assent. Participants were recruited through the school systems and the school selection are informed by demographic characteristics to minimize selection bias. Participants are reimbursed and the reimbursement rates vary across sites based on the costs of living. Typical compensation

includes $200 for the parent/guardian and $100 worth of gifts for the child. See Garavan 2018 for more details.

NCANDA Study: Participants were recruited through announcements distributed to student populations at local schools and colleges, public notices, and targeted catchment-area calling. Interested participants and one biological parent completed a phone screen that assessed eligibility and preliminary characterization of risk factors. Adult participants consented to participating, and minors provided written assent along with consent from a parent/legal guardian. Participants were compensated for completing all baseline sessions, and parents were compensated for completing the baseline interview, with total compensation ranging from \$200 to \$225 per family across sites. Participants discovered to be ineligible received partial compensation based on the measures completed before exclusion. See Brown et al., 2015 for more information.

Ethics oversight

ABCD Study: All procedures were approved by site-level institution research ethics boards and all experimental procedures were in compliance with the Declaration of Helsinki and subsequent revisions (Pfefferbaum et al., 2017). The ABCD study was approved by the Institutional Review Board at University of California, San Diego. Parents or guardians provided written consent while the child provided written assent.

NCANDA Study: Each site provided independent IRB approval with parent approval and assent for youth participants under age 18 and participant consent for those over age 18. As of 20th December 2022, ethics approval was centralized to the UCSD site (#120915).

Note that full information on the approval of the study protocol must also be provided in the manuscript.

Field-specific reporting

Please select the one below that is the best fit for your research. If you are not sure, read the appropriate sections before making your selection.

☒ Life sciences ☐ Behavioural & social sciences ☐ Ecological, evolutionary & environmental sciences

For a reference copy of the document with all sections, see nature.com/documents/nr-reporting-summary-flat.pdf

Life sciences study design

All studies must disclose on these points even when the disclosure is negative.

Sample size

We utilized a large sample (N= 1866) which was chosen based on the number of subjects who had usable and availabile neuroimaging data after strict quality control and who met inclusion criteria based on demographic covariates as described in the main text.

Data exclusions

From the original ABCD cohort (N = 11,868), we used the following pre-defined exclusion criteria:

1. MRI Quality Control: Participants who did not meet stringent MRI quality control standards or failed other exclusion criteria as detailed in Chen et al. (2022) and Ooi et al. (2022) were excluded (N = 9506). Given the susceptibility of pediatric neuroimaging data to motion artifacts, rigorous quality control is essential to avoid biased findings.

2. Scanner Type: Participants scanned on Philips scanners were excluded in accordance with ABCD consortium recommendations (N = 2).

3. Missing Family History Data: Participants without sufficient information to determine family history of substance use disorder (SUD) group membership were excluded (N = 109).

4. Missing Maternal Substance Use Data: Participants missing information on maternal substance use, a key covariate due to its influence on neurodevelopment, were excluded (N = 59).

5. Adopted Participants: Adopted youth were excluded due to potential inaccuracies in reported family history data (N = 9).

6. Substance Use History: Participants who had already initiated substance use were excluded, as our focus was on pre-exposure differences related to family history (N = 54).

7. Sex: Participants with discrepancies between reported sex and sex assigned via salivary DNA were excluded due to potential clerical errors or gender diversity. The latter group was too small to analyze appropriately (N = 17).

8. Missing Household Income Data: Participants missing household income data were excluded, as socioeconomic status is a critical covariate per NIMH recommendations (N = 75).

9. Missing Parental Mental Health Data: Participants missing information on parental mental health were excluded, again in line with NIMH guidance for covariate inclusion (N = 79).

10. Missing Pubertal Status: Participants missing pubertal development data were excluded, as pubertal stage is a known moderator of neurodevelopment, as noted by Reviewer 2 (N = 14).

11. TE Outliers: Participants with mean global transition energy (TE) values identified as statistical outliers—defined as >1.5 times the interquartile range above the 75th percentile or below the 25th percentile—were excluded to prevent skewed results (N = 58). This group also exhibited higher in-scanner motion compared to the remaining sample.

For the NCANDA dataset,

The general exclusion criteria used in the NCANDA replication cohort included removing individuals with fewer than 7.8 minutes of usable resting-state fMRI frames after censoring for framewise displacement greater than 0.3 mm/TR, censoring individual frames and adjacent frames (one before and two after), removing uncensored segments shorter than five contiguous frames, excluding subjects who exceeded alcohol, tobacco, marijuana, or other drug usage thresholds as defined in prior work (N = 132), excluding individuals aged 16 years or older (N = 260) to better align with the younger ABCD sample and minimize substance exposure, and excluding additional outlier subjects whose mean global transition energy exceeded ±3 scaled median absolute deviations from the cohort median (N = 76), resulting in a final matched cohort of 64 individuals selected via 500 iterations of a matching algorithm that minimized differences in sex, age, and mean framewise displacement.

Replication

We replicated our main findings in several ways: (i) re-clustering with k=5 brain states, (ii) utilizing individual structural connectomes (SC) in a cortex-only parcellation, and (iii) in a cohort of sex, age, and in-scanner motion-matched subjects from an external dataset: the National Consortium on Alcohol and NeuroDevelopment in Adolescence (NCANDA). We also re-ran ANCOVA models by stratifying our cohort in various ways: (i) a single site with the largest number of subjects, (ii) within each MRI scanner model, and (iii) within each income category. Overall, these analyses confirmed the robustness and reliability of our findings across various conditions and datasets, but indicate a possible influence of socioeconomic and demographic factors.

We conducted each replication analysis once, using independent datasets or processing pipelines as outlined. Stratified ANCOVA models (e.g., by site, scanner model, or income) were each conducted once per subgroup. In all cases, these were independent replication analyses designed to test the generalizability and robustness of our findings across variations in data structure, sample composition, and acquisition parameters.

Randomization

Randomization was not applicable to this study, as we examined naturally occurring group differences between youth with and without a family history of substance use disorder (SUD) using data from a large, publicly available observational dataset.

Blinding

Blinding was not relevant to this dataset for the same reason as listed above.

Reporting for specific materials, systems and methods

We require information from authors about some types of materials, experimental systems and methods used in many studies. Here, indicate whether each material, system or method listed is relevant to your study. If you are not sure if a list item applies to your research, read the appropriate section before selecting a response.

Materials & experimental systems

n/a	Involved in the study
☒	☐ Antibodies
☒	☐ Eukaryotic cell lines
☒	☐ Palaeontology and archaeology
☒	☐ Animals and other organisms
☒	☐ Clinical data
☒	☐ Dual use research of concern
☒	☐ Plants

Methods

n/a	Involved in the study
☒	☐ ChIP-seq
☒	☐ Flow cytometry
☐	☒ MRI-based neuroimaging

Plants

Seed stocks

N/A

Novel plant genotypes

N/A

Authentication

N/A

Magnetic resonance imaging

Experimental design

Design type	Resting-state and diffusion MRI
Design specifications	Each subject was scanned 4 times for 5 minutes each for rsfMRI.
Behavioral performance measures	No behaviors (resting state)

Acquisition

Imaging type(s)	Functional MRI and diffusion MRI
Field strength	3T MRI
Sequence & imaging parameters	Multiple models of scanners were used in this dataset, so the sequences were variable, but "The fMRI acquisitions (2.4mm isotropic, TR=800ms) also use multiband EPI with slice acceleration factor 6. Each of the dMRI and fMRI acquisition blocks include fieldmap scans for B0 distortion correction. " (Hagler et al., 2019).
Area of acquisition	Whole brain

Diffusion MRI ☒ Used ☐ Not used

Parameters: From Hagler et al. (2019) describing ABCD study MRI acquisition: "The dMRI acquisition (1.7mm isotropic) uses multiband EPI (Moeller et al., 2010; Setsompop et al., 2012) with slice acceleration factor 3 and includes 96 diffusion directions, seven b=0 frames, and four b-values (6 directions with b=500s/mm2, 15 directions with b=1000s/mm2, 15 directions with b=2000s/mm2, and 60 directions with b=3000s/mm2). Each of the dMRI and fMRI acquisition blocks include fieldmap scans for B0 distortion correction. "

Preprocessing

Preprocessing software

As described in Chen et al. (2022): "FreeSurfer 5.3.0; FSL 5.0.8; Our preprocessing code can be found here: https://github.com/ThomasYeoLab/CBIG/tree/master/stable_projects/preprocessing/CBIG_fMRI_Preproc2016."

Normalization

We analyzed rsfMRI collected during the baseline assessment of the multi-site ABCD study. Minimally processed rsfMRI data underwent pre-processing and quality control as described in Ooi et al. (2022) and Chen et al. (2022). As described in Chen et al. (2022): "We aligned the fMRI data to the T1 images using boundary-based registration (Greve and Fischl 2009, NeuroImage) with FsFast. Cortical surfaces were extracted from the T1 images and aligned to FreeSurfer fsaverage space using FreeSurfer." Upon receiving these pre-processed rsfMRI data, we subsequently removed censored volumes from the rsfMRI time series and normalized the BOLD time series by the mean gray matter BOLD signal (pre-spatial and temporal filtering).

Diffusion MRI (dMRI) was collected at the baseline assessment (Casey et al., 2018). The preprocessed dMRI was further processed by deterministic tractography with SIFT2 global streamline weighting and regional volume normalization. Structural connectomes (SC) were extracted in the FreeSurfer-86 (FS86; cortical and subcortical) for 149 subjects and Desikan-Kiliany 68 (DK68; cortical only) for 2080 subjects. The SC matrices are symmetric with the diagonal (self-connections) set equal to zero.

Normalization template

As described in Chen et al. (2022): "FreeSurfer fsaverage6 surface space"

Noise and artifact removal

Nuisance covariates (global signal, motion correction parameters, average ventricular signal, average white matter signal, and their temporal derivatives) were regressed out of the BOLD rsfMRI timeseries.

Volume censoring

Volumes (as well as one volume before and two volumes after) were marked as outliers if they had framewise displacement (FD) > 0.3 mm or DVARS > 50. Uncensored segments of data containing fewer than five contiguous volumes were also censored. Functional runs were excluded if over half of the volumes were censored or BBR costs were >0.6.

Statistical modeling & inference

Model type and settings

We conducted a series of analyses of covariance (ANCOVA) to examine the effects of family history of SUD and its interaction with sex on mean and pairwise TE at global, network, and regional levels. All models included the following independent variables: sex, age, family history of SUD (FH+ vs. FH-), race/ethnicity, household income, parental education, parental mental health issues, prenatal substance exposure, MRI scanner model, in-scanner motion (mean framewise displacement), and puberty status. Additionally, three interaction terms were included: puberty and sex, family history of SUD and sex, and family history of SUD and income. ANCOVA models were run on mean global network transition energy (TE), 16 pairwise global TEs, 9 mean network TEs, 3x16 pairwise network TEs, 86 mean regional TEs and 86x16 pairwise regional TEs. The direction of effects was determined using post hoc unpaired t-tests on TE values that showed significant differences in ANCOVA models.

Effect(s) tested

We tested whether youth with a family history had altered brain dynamics (i.e. transition energy) and whether this was modulated by sex. We applied k-means clustering to regional rsfMRI time series data to identify k recurring patterns of brain

activity, termed "brain states". For each subject, we assigned each individual frame to a brain state and calculated subject-specific brain state centroids. We then applied NCT to calculate the global, network, and region-level TE required to complete brain state transitions. For this, we utilized a group-average structural connectome derived from dMRI.

Specify type of analysis: ☒ Whole brain ☐ ROI-based ☐ Both

Statistic type for inference (See Eklund et al. 2016): *Specify voxel-wise or cluster-wise and report all relevant parameters for cluster-wise methods.*

Correction: Benjamini-Hochberg FDR

Models & analysis

n/a	Involved in the study
☒	☐ Functional and/or effective connectivity
☒	☐ Graph analysis
☒	☐ Multivariate modeling or predictive analysis
